# Kinetic Studies of Acetyl Group Migration between the Saccharide Units in an Oligomannoside Trisaccharide Model Compound and a Native Galactoglucomannan Polysaccharide

**DOI:** 10.1002/cbic.202100374

**Published:** 2021-09-02

**Authors:** Robert Lassfolk, Sara Bertuzzi, Ana Ardá, Johan Wärnå, Jesús Jiménez‐Barbero, Reko Leino

**Affiliations:** ^1^ Laboratory of Molecular Science and Engineering Åbo Akademi University 20500 Turku Finland; ^2^ Chemical Glycobiology Laboratory CIC bioGUNE Bizkaia Technology Park, Building 800 48160 Derio Spain; ^3^ Ikerbasque, Basque Foundation for Science Plaza Euskadi 5 48009 Bilbao Spain; ^4^ Laboratory of Industrial Chemistry and Reaction Engineering Åbo Akademi University 20500 Turku Finland; ^5^ Department of Organic & Inorganic Chemistry University of the Basque Country, UPV/EHU 48940 Leioa Bizkaia Spain

**Keywords:** acyl migration, C-type lectin, carbohydrates, DC-SIGN, galactoglucomannan

## Abstract

Acyl group migration is a fundamental phenomenon in carbohydrate chemistry, recently shown to take place also between two non‐adjacent hydroxyl groups, across the glycosidic bond, in a β‐(1→4)‐linked mannan trisaccharide model compound. With the central mannoside unit containing acetyl groups at the O2 and O3 positions, the O2‐acetyl was in the earlier study shown to migrate to O6 of the reducing end. Potential implications of the general acyl migration process on cell signaling events and plant growth in nature are intriguing open questions. In the present work, migration kinetics in this original trisaccharide model system were studied in more detail together with potential interactions of the model compound and the migration products with DC‐SIGN lectin. Furthermore, we demonstrate here for the first time that similar migration may also take place in native polysaccharides, here represented by galactoglucomannan from Norway spruce.

## Graphical abstract

**Kinetic calculations** on the migration between saccharide units in a mannan trisaccharide model compounds and a galactoglucomannan polysaccharide were performed. Potential interactions of the model compounds with a lectin were also investigated.

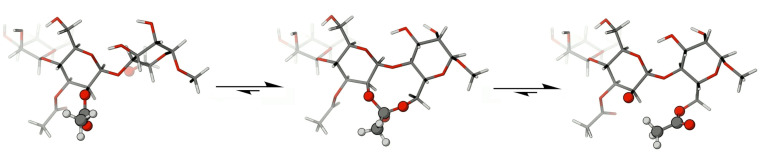

## Introduction

**Scheme 1:**
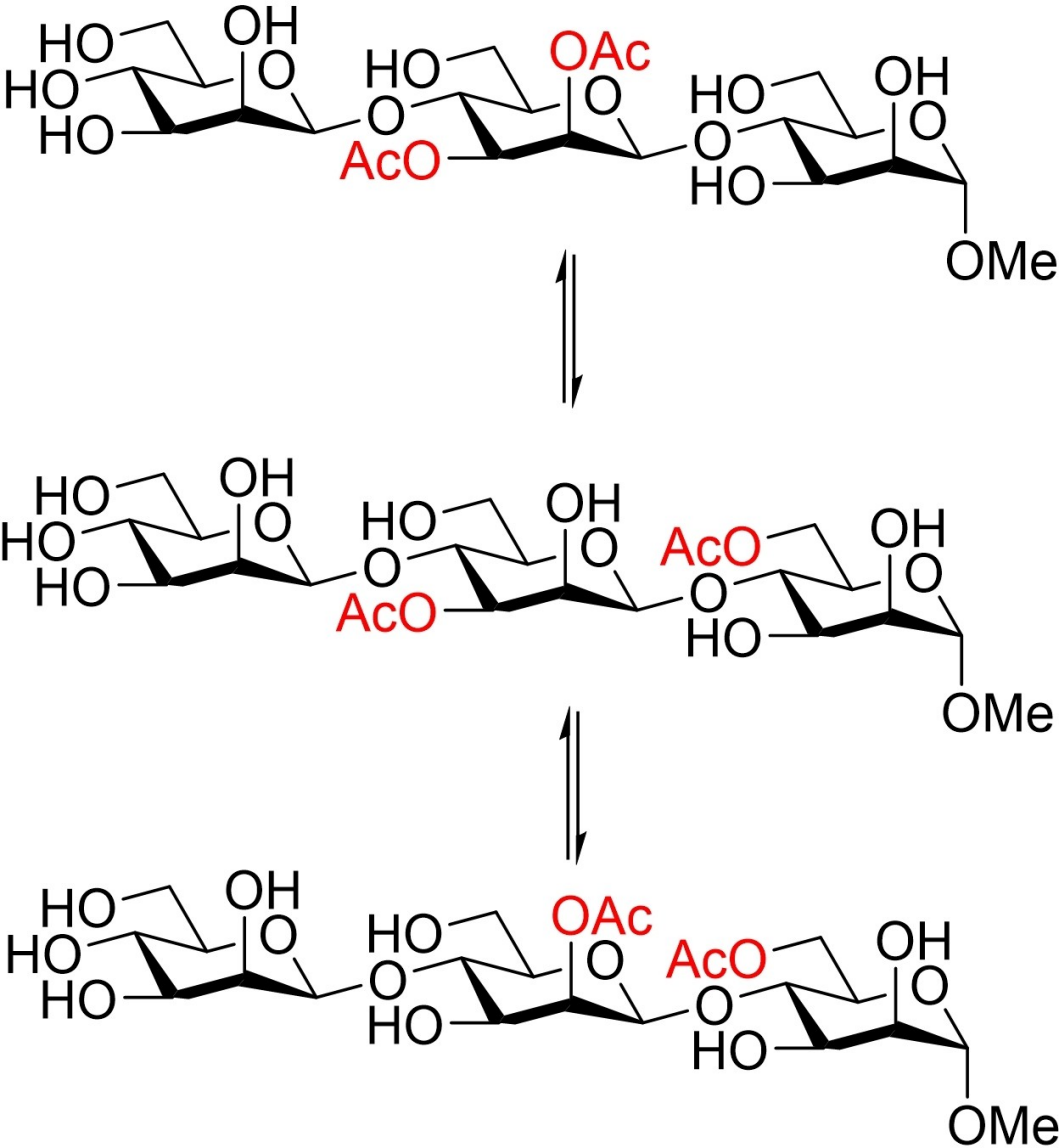
Acetyl migration between the saccharide units in an oligomannoside model compound.

It is well known that acyl groups in polyhydroxyl compounds are prone to migration, as demonstrated for carbohydrates already in 1920 by Fischer.^[1]^ Since then, several studies have been performed, investigating the acyl group migration in different monosaccharide molecules.[^2^, ^3^, ^4^, ^5^, ^6^, ^7^, ^8^, ^9^] One of the key conclusions from the earlier studies is that the migration rate is significantly influenced by the pH of the buffers used.[^2^, ^8^] Between pH=7–8, the rate is linear with respect to the concentration of [OH^−^].^[8]^ Intriguingly, this coincides with the cytoplasmic pH=7–8 in plant cells, which further depends on the type of the plant and its growth environment.^[10]^ Furthermore, it has been observed that the cytoplasmic pH increases during cell growth and development.[^10^, ^11^] In light of these observations, the regulation of biologically active compounds by acetyl groups,^[12]^ and our recent discovery of acetyl group migration taking place between two different saccharide units in an oligosaccharide (Scheme 1),^[13]^ it is tempting to speculate on the possible role of acyl group migration in biological regulation on a more general level. Many of the naturally occurring polysaccharides, including glucans,[^14^, ^15^, ^16^, ^17^] xylans[^18^, ^19^, ^20^] and mannans,[^21^, ^22^, ^23^, ^24^, ^25^, ^26^] contain partially acetylated hydroxyl groups. Both the degree of acetylation and the positions of the acetyl groups depend, besides on the plant itself, also on the development stage of the plant's life cycle.[^27^, ^28^] Evidently, the increase in pH during cell growth and development in a plant should also increase the rate of acetyl group migration in the constituent polysaccharides. This implies that the acetyl groups also would play an important role in the biological activity of polysaccharides. Consequently, more detailed studies on the migration phenomenon could provide insights into the regulation of cell signaling by partially acetylated polysaccharides.

Acemannan, glucomannan, galactomannan and galactoglucomannan (GGM) belong to the class of β‐(1→4)‐linked mannans, found in a variety of plants.[^21^, ^22^, ^23^, ^24^, ^25^, ^26^] Typically, the acetyl groups are located at either the O2 or O3 positions of the mannose units.[^21^, ^22^, ^23^, ^24^] It has also been shown that the O6 positions of the mannose units can, to a minor degree, be acetylated in some mannans.^[26]^ In plants, mannans have various different roles, including acting as cell signaling molecules for plant growth and development.^[29]^ Aside from the role in the host plants, mannans also exhibit other types of biological activity, such as inhibiting the growth of cancer tumors,[^21^, ^30^, ^31^] displaying antioxidant activities,[^32^, ^33^] anti‐inflammatory activity,[^33^, ^34^] immunomodulatory activities,[^35^, ^36^, ^37^] wound healing effects[^38^, ^39^] and more.^[40]^ For many mannans the acetyl groups are crucial for their biological activity.[^40^, ^41^, ^42^, ^43^] Elucidating how these activities are regulated or influenced through acetyl group migration processes could contribute, besides to better utilization of the biological activity of the polysaccharides in therapeutical use, also to understanding the potential internal regulation mechanisms within the plant.

As demonstrated by us in earlier work, conformational flexibility of small oligosaccharides allows them to attain energetically favorable orientations for acetyl group migration even between the different saccharide units.^[13]^ Larger, branched polysaccharides are conformationally more rigid, which may hinder the intramolecular acetyl group migration across the glycosidic bonds, while still being possible between two adjacent hydroxyl groups within the same saccharide unit. Notably, the β‐(1→4)‐linked mannans uptake a two‐fold screw conformation, similar to cellulose, placing the O6 close in space to O2, but due to the axial 2‐OH the polysaccharide chain is more flexible compared to cellulose.[^44^, ^45^, ^46^] The presence of glucose in the backbone does not induce any major conformational effect.^[47]^ The largest effect on the conformational dynamics is inferred by the side chains, particularly the galactose units.^[48]^ The Man:Gal ratio and the type of polymer (block, alternating or random) further influences the rigidity of the polysaccharide in question.^[48]^

Considering that migration across the saccharide units is possible in a trisaccharide model compound,^[13]^ and the close proximity of the O2 and O6 of neighboring saccharide units in mannan polysaccharides, it could be hypothesized that O6 acetylation of the mannose units in glucomannan results from acetyl group migration.^[26]^ The biosynthesis of polysaccharides takes place in the Golgi lumen, where the acetyl groups are also added.^[28]^ After deposition in the cell walls, the degree and pattern of acetylation may change.^[28]^ Potential evidence of acetyl group migration in native polysaccharide molecules could have fundamental significance for our understanding of the regulation of biological activity of carbohydrates and the overall plant glycobiology. In the present work, we provide further insights into the kinetics of acetyl migration in our earlier described oligosaccharide model compounds, and also present the first evidence on migration of acetyl groups in native GGM polysaccharide under similar experimental conditions. Furthermore, we provide the first insights into how the acetyl migration of the trisaccharide model can be modulated in the presence of DC‐SIGN, a model lectin known to bind d‐mannose.

## Results and Discussion

**Figure 1:**
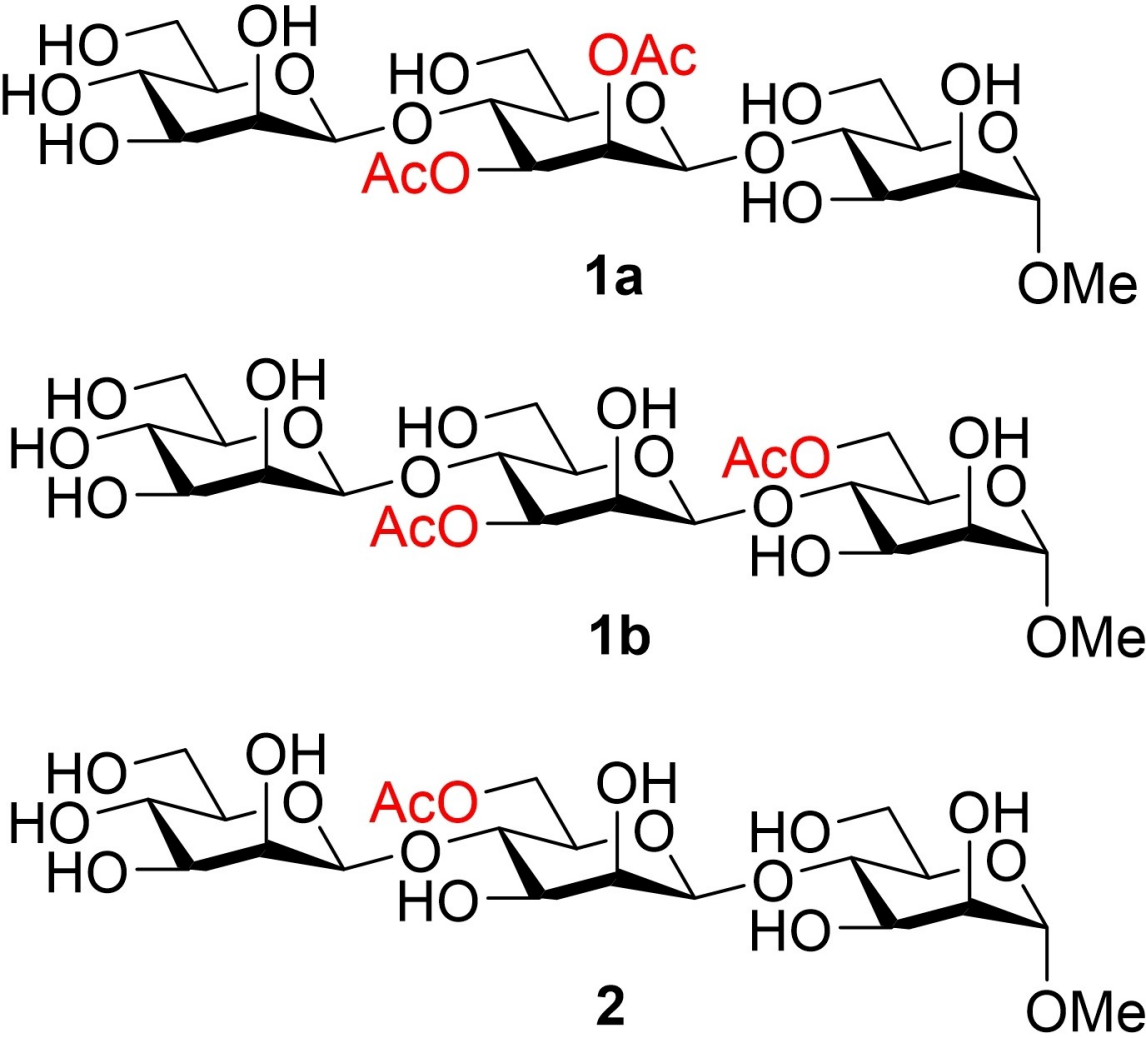
Trisaccharide model compounds investigated in this work.

Acetyl group migration in oligosaccharides was studied in more detail by using the trisaccharide model compounds **1** **a**, **1** **b** and **2** (Figure 1). The synthesis of **1** **a** has been described in our earlier work.^[13]^ Synthesis of the migration product **1** **b** and compound **2** were carried out by similar methods and are described in detail in the supporting information. The trisaccharides **1** **a** and **1** **b** were here used to investigate the kinetics of the acetyl group migration, while the trisaccharide **2** was prepared to investigate the possibility of further migration along the oligosaccharide chain.

### Migration in the trisaccharide model compounds

**Scheme 2:**
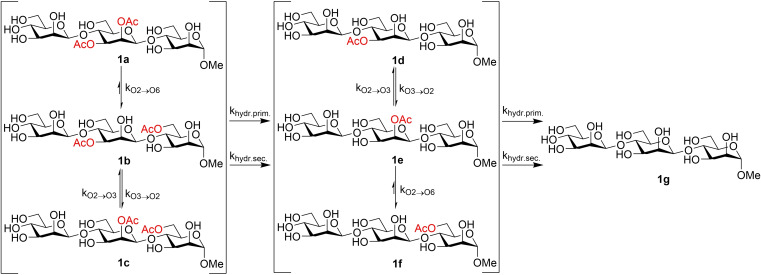
The proposed acetyl group migration and hydrolysis pathway.

In our previous work,^[13]^ the rate constants for intramolecular migration across the saccharide units in the model compound (**1** **a**→**1** **b**) could not be determined under the employed experimental setting due to the change in pH over the long migration time. Here, instead of D_2_O buffer used in the earlier study, the migration was investigated in H_2_O based buffer where the migration is faster, but the hydrolysis is not affected as shown in our earlier study. Two starting points **1** **a** and **1** **b** were selected for following the migration process in order to facilitate the accurate calculation of the rate constants. The migration was followed by water suppressed ^1^H NMR spectroscopy and the ratios were based on the acetyl peaks, being sufficiently separated in the spectrum. Due to complexity of the system, similar types of migration, such as hydrolysis of acetyl groups from the primary positions and the O2→O6 migrations, were set to have equal rate constants (Scheme 2).

It has been established previously that the pH of the buffer significantly influences the acyl group migration rates.[^3^, ^8^] Consequently, also the pH of the solutions was monitored by pH‐meter. When the trisaccharides were initially dissolved in the buffer, the pH changed to 7.95. At the end of the migration (after four weeks), the pH had decreased to 7.70 and 7.75, when starting from compounds **1** **b** and **1** **a**, respectively (See Figure S1 in the supporting information). In an earlier study, Mortensen and coworkers demonstrated a linear correlation between the rate constants and the [OH^−^] concentration, i. e., the pH, indicating that a decrease in pH from 7.95 to 7.75 decreases the rate constants by 37 %.^[8]^ Therefore, for calculating the rate constants in the present work, a correction factor correlating with the pH change was included. This factor is calculated as C(OH)/C(OH)_start_ and is 1 at pH=8, after which it decreases with decreasing pH. The pH did not change significantly at the end of the migration, possibly due to slower hydrolysis of the acetyl groups.

**Figure 2:**
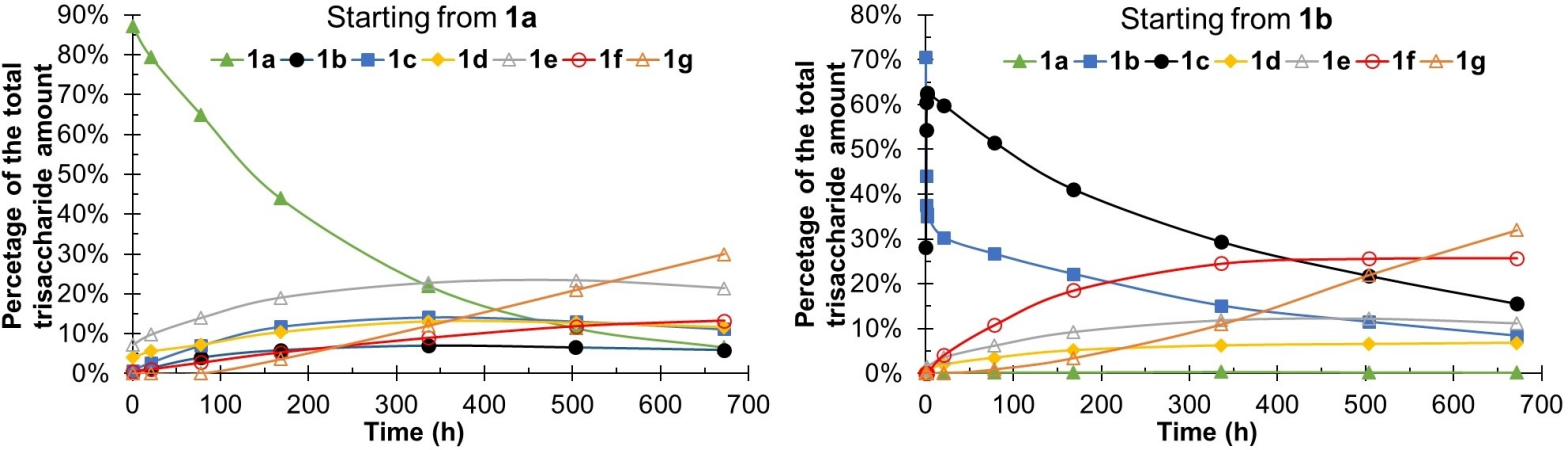
Acetyl group migration starting from the model compounds **1** **a** and **1** **b**. Conditions: 100 mM phosphate solution with 10 % D_2_O, pH=8, 25 °C.

The rapid increase in the concentration of **1** **d** and **1** **e** cannot be explained by the **1** **a**→**1b** migration alone, followed by hydrolysis from the primary position (Figure 2). Similarly, when starting from **1** **b**, the concentration of **1** **f** is increasing faster than those of **1** **d** and **1** **e**. Evidently, when calculating the rate constants, hydrolysis from the secondary hydroxyl groups should also be considered. Such hydrolysis is almost as fast as the hydrolysis from the primary positions, estimated to be only 20 % slower in the trisaccharide model compounds (Table 1). In most of the earlier migration studies on monosaccharides containing a free primary hydroxyl group, it has been assumed that the acyl group is hydrolyzed from the primary position.[^2^, ^3^, ^13^] Negligence of the hydrolysis from secondary positions is likely due to, besides the significant preference of the acyl groups for the primary position, also the fast migrations; the experimental times in earlier studies have typically been shorter than in our present work. It is clear, however, that in future investigations, the possible hydrolysis of the acetyl groups also from the secondary hydroxyls of carbohydrate moieties should be taken into account.

**Table 1:** The rate constants at pH=8 calculated for the acetyl group migration in Scheme 2.^[a]^

	Rate constants [h^−1^]
k_O2→O6_	2.06E‐03±1.85E‐04
k_O3→O2_	1.88E+00±2.96E‐01
k_O2→O3_	1.01E+00±1.84E‐01
k_prim.hydr_	1.93E‐03±1.29E‐04
k_sec.hydr_	1.56E‐03±7.56E‐05

[a] Conditions: 100 mM phosphate solution with 10 % D_2_O at 25 °C, starting pH=8.

The migration between the O2 and O3 in the trisaccharides is approximately 1000 times faster than any other migration, as expected based on the previous study on acetyl group migration in monoacetylated mannopyranoside.^[13]^ The ratio between the acetylated O2 and O3 positions is ca. 65 : 35. This indicates a slightly higher preference for the O2 position compared to the earlier studied β‐(1→4)‐mannan disaccharide, where ca. 62 % of the acetyl groups were located at O2.^[13]^ The migration seems to be faster in the 100 mM phosphate solution used here than in the 10 mM phosphate solution used for the monosaccharide in our earlier work.^[13]^ It appears that increasing the ion strength of the solution speeds up the migration, but since different compounds are compared, the differences in the migration rate could also be due to intrinsic differences in the compounds studied.

The **1** **a**→**1b** migration is slow and the **1** **b**→**1a** back‐migration is negligible in the mannan trisaccharide. When starting from **1** **b**, a maximum 0.4 % concentration of the trisaccharide **1** **a** is formed and, consequently, the rate constant could not be calculated. This was expected, as the most stable position for the acyl groups in monosaccharides is indeed the primary hydroxyl position, in accordance with earlier studies.[^2^, ^3^, ^13^] It can also be observed that the rate constant for the O6→O4 migration is ca. 5—10 times lower than the O4→O6 migration in monosaccharides.[^2^, ^13^] This could also be seen in compound **2**, where no or minimal migration and mostly hydrolysis only took place. Any compounds potentially formed through migration had concentrations too low to be observed and characterized by NMR spectroscopy. The conformational freedom of the primary position of the mannan trisaccharides could prevent the acetyl group from obtaining a suitable conformation for the O6→O2 migration to take place. The migration between the saccharide units is slightly faster than the hydrolysis from the primary position, indicating that the concentration of products with O6‐Ac will not be significant. Starting from compound **1** **b**, **1** **f** reached a maximum concentration of 26 %, but this was partially due to hydrolysis from the secondary hydroxyl positions. When starting form **1** **a**, no product except the final product **1** **g** had a higher concentration than 25 % throughout the study, even though some hydrolysis, around 10 %, had taken place already at the first measurement point.

The slow O2→O6 migration is most likely due to the flexibility of the trisaccharide having several conformational energy minima,^[13]^ making it difficult to attain the required conformation. Consequently, also the temperature may influence the migration rate. Since one of the limiting factors under the studied conditions, besides pH, is the conformation of the trisaccharide,^[13]^ it is possible that with increasing temperature the rate of conformational interchange would increase more than the rate of hydrolysis. This would mean that at higher temperatures the O2→O6 migration could increase more than the hydrolysis from the primary positions. From a biological point of view, the build‐up of the Ac‐O6 in polysaccharides at higher temperatures could increase the cell signalling and, therefore, increase the rate at which the plant grows. Further studies regarding this are, however, required to estimate more precisely how the temperature affects the rate of migration and hydrolysis of acetyl groups in nature.

### Migration in GGM

**Figure 3:**
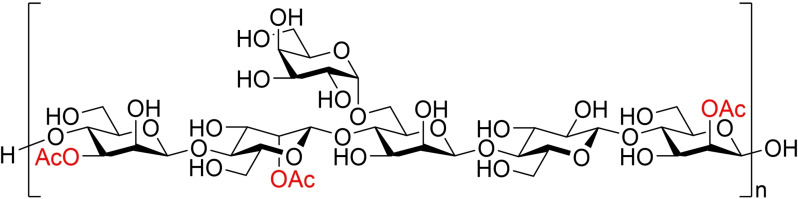
Generic structure of galactoglucomannan.

To investigate the possible migration in native polysaccharides, a GGM sample from Norway spruce (*Picea abies*) was used.^[23]^ GGM (Figure 3) consists of both glucose units in the backbone, as well as galactose in the α‐(1→6)‐sidechains linked to the mannoside units.^[49]^ In Norway spruce, the ratio between Man:Glc:Gal is typically approximately 4 : 1 : 0.1.[^50^, ^51^] The rather low galactose content should not have a significant influence on the linearity of GGM, allowing the O6 and O2 positions of the neighboring mannose units to be sufficiently close in space to allow for migration of acetyl groups to take place. The degree of acetylation in isolated GGM is approximately 65 % in the mannose units, which should be sufficient for observing the migration, if present.^[23]^

**Figure 4:**
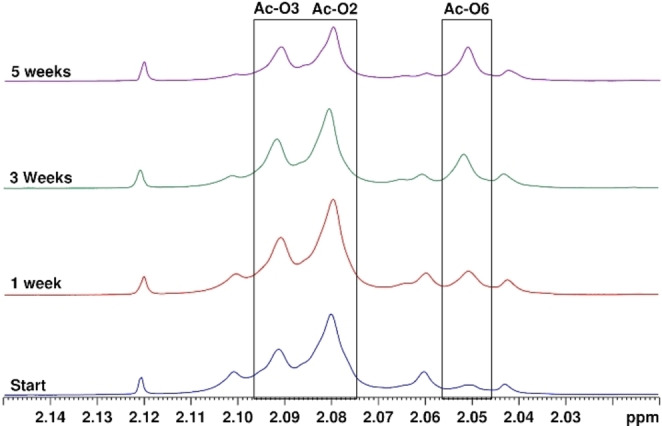
^1^H‐NMR spectra of the acetyl group migration process in GGM. The areas where the corresponding acetyl groups in the trisaccharides were located are marked with boxes. Conditions: 100 mM phosphate solution with 10 % D_2_O at 25 °C, starting pH=8.

The migration in GGM was followed by water suppressed ^1^H NMR spectroscopy in the same buffer as used for the trisaccharide model compound, as even here the major acetyl peaks are separated to sufficient degree in the spectrum. Due to the slow migration, several weeks were needed to analyze the possible migration result. It could be observed that in the area where the O6 acetyl groups in the model compounds resonate, a peak increased in intensity, while the other acetyl peaks decreased (Figure 4). By using water‐suppressed HSQC and 1D HMBC NMR spectroscopic measurements, it could be observed that more acetylated O6 signals in the mannose units are present after 5 weeks of migration than at the start. In the HSQC, the acetylated O6 protons in GGM are exactly matching the same protons in model compound **2** and these signals could not be seen at the starting point of the migration. As shown earlier for the more flexible trisaccharide that O2→O6 migration takes place intramolecularly between two different saccharide units, the same could be expected for the conformationally more rigid polysaccharide. Also, because the conformation of GGM resembles that of cellulose, the O3 and O6 hydroxyls in the neighboring saccharide units are likely positioned too far away in space for migration to take place. To our knowledge, this is the first evidence of acetyl migration taking place also between the saccharide units in a native polysaccharide.

**Scheme 3:**
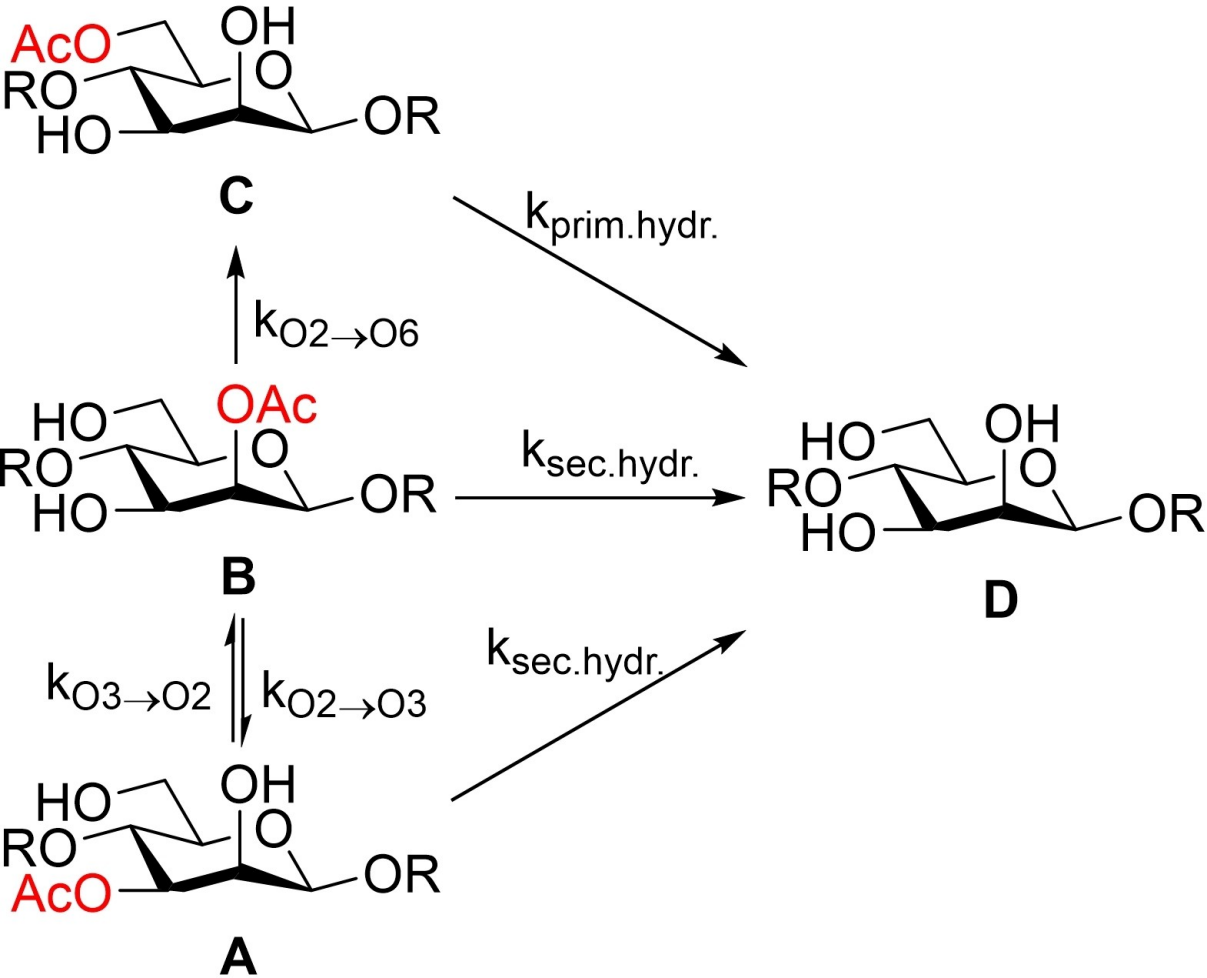
The kinetic model used to determine the rate constants for acetyl group migration in GGM.

**Figure 5:**
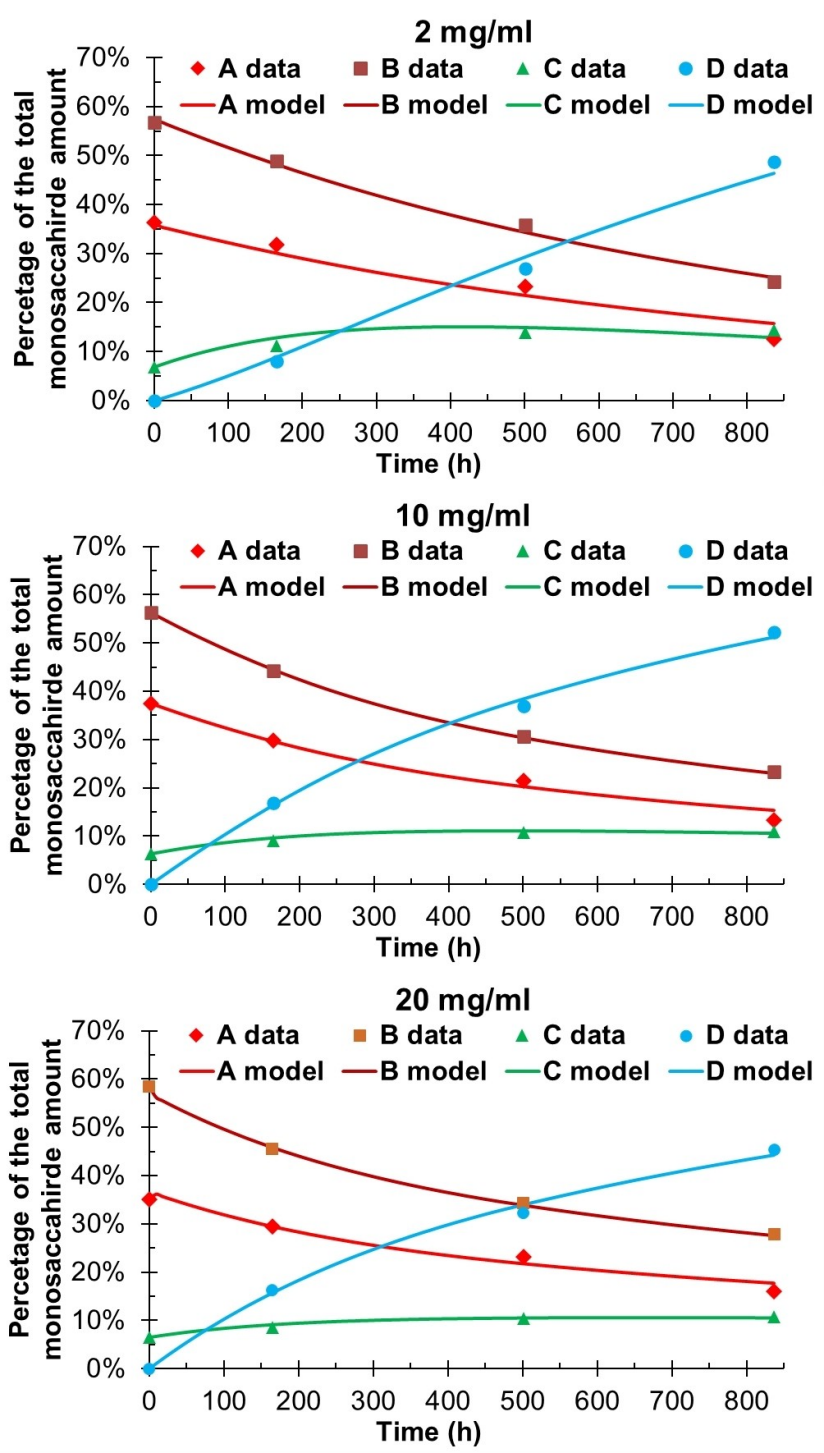
The migration in GGM at different concentrations. Conditions: 100 mM phosphate solution with 10 % D_2_O, pH=8, 25 °C. Degree of explanations: 2 mg/ml: 98.25 %; 10 mg/ml: 99.55 %; 20 mg/ml: 99.43 %.

The rate constants were then calculated for the migration and hydrolysis in GGM to shed further light on the migration process. Due to the structural complexity of GGM, a simplified model had to be used. The model, broken down to monosaccharide units, is illustrated in Scheme 3. The model is based on the acetyl group peaks in the ^1^H NMR spectra in GGM being located at the same positions as the corresponding peaks in the trisaccharides, in accordance with Figure 4. As GGM already has an equilibrium between the acetylated O2 and O3 positions, one of the rates had to be based on the corresponding migration in the trisaccharide. Thus, *k*_O2→O3_ was set to 1 h^−1^. Furthermore, due to the possibility that the polysaccharide chains could interact with each other through hydrogen bonding, more than between the model trisaccharide chains, concentrations of 2, 10 and 20 mg/ml were used (Figure 5). The pH of the migration studies was also monitored (See Figure S2 in the supporting information).

At the concentration of 2 mg/ml, the O2→O6 migration rate in GGM seems to be closer to that of the trisaccharide model compounds, while by increasing the concentration to 10 and 20 mg/ml the migration becomes slower (Table 2). The ratio between acetylated O2 and O3 in GGM is ca 61 : 39. This is similar to many of the reported values for the ratio between acetylated O2 and O3 in isolated GGM.[^23^, ^52^] The rate of hydrolysis from the primary hydroxyl groups decreases with increasing concentration, while the hydrolysis from the secondary hydroxyl groups appears to increase with increasing concentration. This is also seen in Figure 5, where the concentration of unit **D** is increasing slower in the beginning and faster towards the end for the 2 mg/ml sample, while for both 10 and 20 mg/ml samples the hydrolysis is noticeably higher at the start. This implies that at 2 mg/ml sample concentration the acetyl groups are more prone to O2→O6 migration, while at 10 and 20 mg/ml the migration slows down significantly. At the concentration of 2 mg/ml, it appears that unit **C** reaches a constant concentration of 15 %, while at 10 and 20 mg/ml the concentration of unit **C** remains at around 11 %. The reason for both the observed hydrolysis and the difference in the migration rate could be due to interactions between the polysaccharide chains at higher concentrations. These interactions could possibly not allow GGM to attain the necessary conformation for O2→O6 migration. Consequently, the O2→O6 migration is not favored and the hydrolysis from the secondary hydroxyl groups becomes more prominent at higher GGM concentrations. How exactly the rate difference at different concentrations could be expressed in plant cells is uncertain at present. Perhaps it could play a role during cell division, when the cell wall between the two cells is forming and not yet well defined. The rate constants for migration and hydrolysis should, however, be taken as an approximation due to the simplified nature of the model. This also becomes evident in the significant calculated errors, especially in the hydrolysis constants, but nevertheless provides a preliminary estimation of the rate constant range.

**Table 2:** The rate constants at pH=8 for the kinetic model in Scheme 3 at concentrations of 2, 10 and 20 mg/ml.^[a]^

	2 mg/ml [h^−1^]	10 mg/ml [h^−1^]	20 mg/ml [h^−1^]
k_O2→O6_	1.50E‐03±8.36E‐04	8.41E‐04±4.54E‐04	7.08E‐04±5.88E‐04
k_O3→O2_	1.60E+00±1.65E‐01	1.51E+00±8.28E‐02	1.56E+00±8.42E‐02
k_O2→O3_	1.00E+00^[b]^	1.00E+00^[b]^	1.00E+00^[b]^
k_prim.hydr_	3.72E‐03±2.46E‐03	2.35E‐03±1.72E‐03	2.06E‐03±2.58E‐03
k_sec.hydr_	2.60E‐04±5.22E‐04	1.16E‐03±2.76E‐04	1.24E‐03±3.60E‐04

[a] Conditions: 100 mM phosphate solution with 10 % D_2_O at 25 °C, starting pH=8. [b] Locked because GGM already have equilibrium.

Due to the low galactose content in GGM and the glucose units in the backbone not having any major conformational effects, it can be expected that the migration between the saccharide units also takes place in other β‐(1→4)‐linked mannans. The acetyl group migration in polysaccharides could very well be an internal way to regulate their biological activity. Considering that the pH increases during the cell growth and development, the formation of the acetylated O6 units in polysaccharides should subsequently increase, which could be a way to increase the cell signaling for plant growth and development. The acetyl groups are crucial for many of the biological activities possessed by mannans in medicinal use,[^40^, ^41^, ^42^, ^43^] and, therefore, it should become important to investigate the possible connection between the acetyl group migration and biological activity. The migration could potentially form the active compound from the prodrug, but could also deactivate the biologically active form. Deeper understanding of the role and process of acetyl migration could enable the optimization and tailoring of the biological activity of polysaccharides by enhancing or suppressing the migration. Also, for this, further studies are needed.

### Influence of DC‐SIGN lectin on the migration of the trisaccharide model compounds

In order to investigate if the acetyl group migration observed for the trisaccharides alone could be affected by the presence of a lectin, DC‐SIGN EDC (extracellular domain) was employed. DC‐SIGN is one of the most widely studied members of the human C‐type lectin family, with the primary Ca^2+^ ion, which directly coordinates the bound‐sugar, located in a solvent‐exposed surface of the carbohydrate recognition domain (CRD).

This lectin is expressed on the surface of dendritic cells and it has been found to be involved in crucial biological processes, mainly related with immune response.[^53^, ^54^, ^55^] Its binding preference for d‐mannose and l‐fucose sugar units has been extensively studied.[^56^, ^57^, ^58^, ^59^] Herein, the evolution of compounds **1** **a**, **1** **b** and **2** in the presence of DC‐SIGN was followed over time by acquiring ^1^H NMR and ^1^H‐^13^C HSQC spectra, and the interaction with the lectin was investigated through Saturation Transfer Difference‐NMR (STD‐NMR) experiments.

The STD‐NMR experiment is a powerful ligand‐based NMR tool largely applied for the study of low affinity ligand‐protein systems. With this technique, it is possible to obtain information on the molecular basis of the interaction between a receptor and a ligand. The STD effect is the result of the transfer of magnetization from the protons of the protein to the protons of the studied ligand in close contact to the protein surface. Thus, the STD spectra report on the ligand's protons nearest to the protein surface, the ligand binding epitope, providing exquisite structural information about the molecular recognition event.[^60^, ^61^, ^62^, ^63^] For all the compounds the same set of experiments were acquired at t_0_ and after 30 days (t_1_).

**Figure 6:**
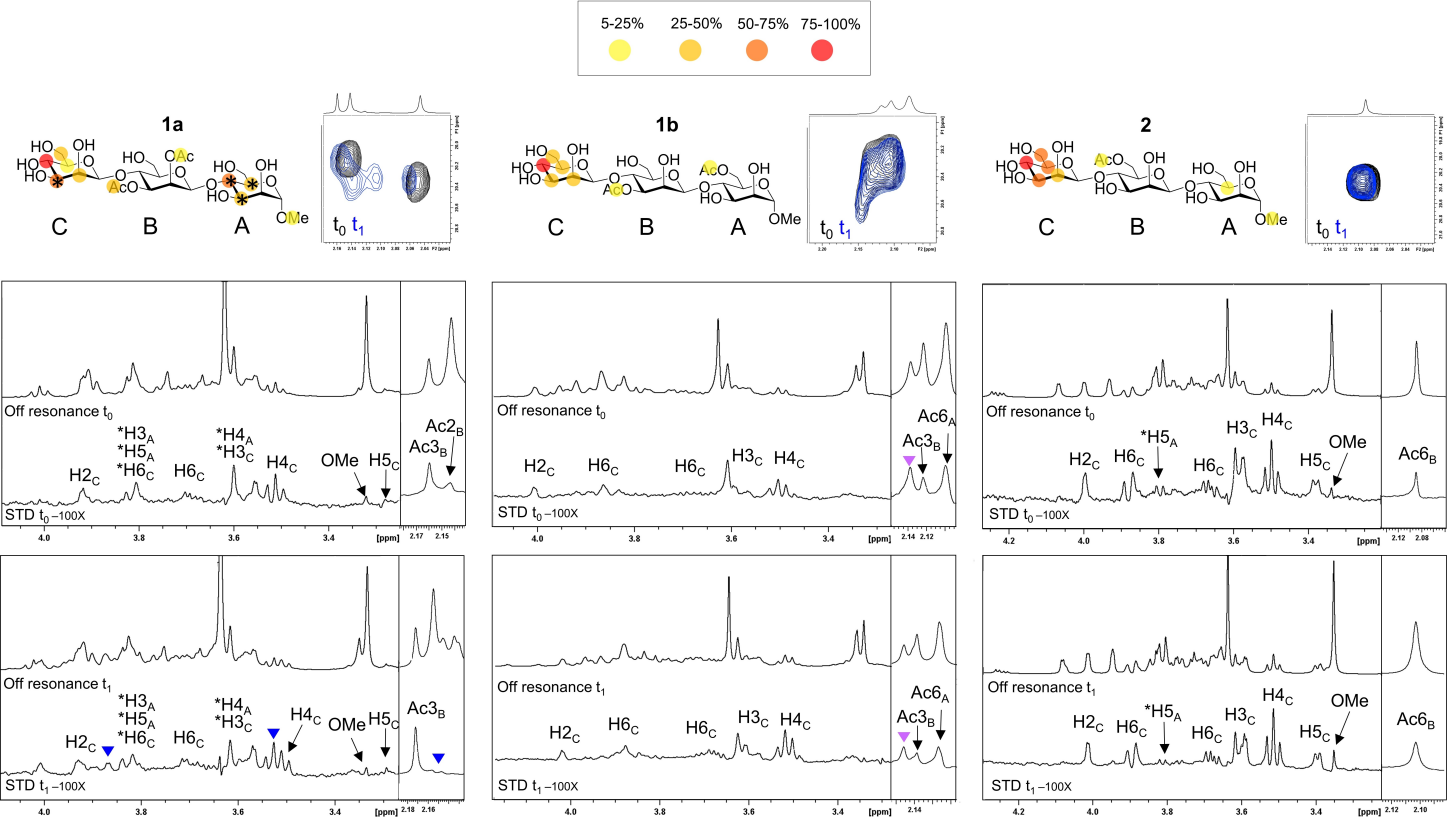
NMR experiments of compounds **1** **a**, **1** **b** and **2** with DC‐SIGN ECD. Top figure: epitope mapping of compound **1** **a** (left), **1** **b** (centre) and **2** (right) with colour legend reported above and ^1^H‐^13^C HSQC expansion of the respective acetyl‐region at t_0_ (black) and t_1_ (blue); STD‐NMR spectra section: off‐resonance spectrum and STD spectrum with irradiation at δ 0.8 ppm and compounds **1** **a** (left), **1** **b** (centre) and **2** (right) at t_0_ (upper panels) and t_1_ (lower panels). Annotations of the main ^1^H‐STD NMR signal are reported in the STD spectrum. The lectin:ligand molar ratio was 1 : 100 for all the samples (being DC‐SIGN ECD at a concentration of 5 μM for the tetramer) and the STD experiments were acquired with 2 seconds of saturation time. The asterisk indicates signals that cannot be unequivocally assigned due to signal overlapping. Blue triangles represent new signals in the STD spectrum appeared at t_1_ and absent at t_0_. Purple triangles represent signals appeared at t_0_ as a result of fast migration in solution.

In all cases, the binding epitopes obtained from STD‐NMR experiments are in agreement with the mannose‐recognition mode described for DC‐SIGN and binding occurs through the non‐reducing end of the trisaccharide (residue C) with hydroxyl groups O3 and O4 simultaneously coordinating the primary Ca^2+^ ion (Figure 6).[^64^, ^65^] The possibility of a different recognition mode involving hydroxyl groups O2 and O3 of the reducing‐end residue (A) was investigated for **1** **a**, since some signals in the STD NMR spectrum could not be unequivocally assigned due to signal overlapping (indicated with an asterisk in Figure 6). Nevertheless, docking analysis excluded this possibility because the interaction through O2 and O3 calcium coordination generated steric clashes.

For compound **1** **b**, acetyl group migration between O2 and O3 occurred immediately after sample preparation, while for compound **1** **a** only partial acetyl migration was observed after one month. This is slower than in the absence of the lectin. After this period, the obtained STD NMR spectrum was essentially the same as at t_0_. For compound **2**, minor migration and hydrolysis was observed, which again did not affect the interaction with the lectin.

Overall, from the analysis of these NMR experiments, the migration of the acetyl groups appears to be much slower in the presence of the model lectin. Thus, the presence of DC‐SIGN modifies the acetyl migration event, slowing it down considerably.

## Conclusions

The aim of this study was to investigate the migration between saccharide units in oligomannoside model compounds and native galactoglucomannan model polysaccharide in more detail. Kinetic calculations were performed on the complex migration pathway to provide better understanding of the rates at which the migration between β‐(1→4)‐linked mannose units takes place. We could also demonstrate, for the first time, that migration between saccharide units is possible also in natural polysaccharides. With the help of more detailed future studies, this could impact our understanding of how biological activity is regulated in polysaccharides, both within the plant and in therapeutical use.

Acyl migration across the glycosidic bond, between two non‐adjacent hydroxyl groups in oligo‐ and polysaccharides is a newly discovered phenomenon. Understanding of its biological significance requires further in‐depth studies. Also, investigations of other types of polysaccharides are required to gain fundamental knowledge on the different migration processes. Open questions at present include, for example, whether the migration between the saccharide units, across a glycosidic bond, could also take place in the absence of primary hydroxyl groups, e. g., in xylans, or how the configurational and conformational orientation of the secondary O2 influences the migration processes.

## Experimental Section

**General**: For following the migration process and identification and characterization of the new compounds a Bruker Avance‐III spectrometer operating at 500.20 MHz (^1^H) and 125.78 MHz (^13^C) equipped with a Prodigy BBO CryoProbe or a Bruker Avance‐III spectrometer operating at 600.16 MHz (^1^H) and 150.91 MHz (^13^C) equipped with a Prodigy TCI CryoProbe was used. The characterization was performed using a standard set of 1D and 2D NMR spectroscopic techniques: ^1^H, ^13^C, 1D‐TOCSY, DQF‐COSY, Multiplicity edited HSQC (CH and CH_3_ positive, CH_2_ negative, both coupled and decoupled), and HMBC. The migration was followed with water suppressed ^1^H, water suppressed Multiplicity edited HSQC and 1D HMBC.

**Preparation of migration samples**: For monitoring the acetyl group migration by NMR spectroscopy, a phosphate solution was used. First, a 100 mM phosphate buffer with 10 % D_2_O and the pH=8 was prepared. A concentration of 2 mg/ml was used for the trisaccharide migration studies.

**Migration Kinetics and Modeling**: The reaction kinetics for the acetyl group migration for the trisaccharides **1** **a** and **1** **b** was described with a reversible (O2⇌O3 migration) and irreversible (hydrolysis and O2→O6 migration) first order reaction scheme as follows

$$r_{1}=k_{O2\to O6}\times c\left(1a\right)\times c\left(OH\right)$$

$$r_{2}=k_{sec.hydr.}\times c\left(1a\right)\times c\left(OH\right)$$

$$r_{3}=k_{O3\to O2}\times c\left(1b\right)\times c\left(OH\right)-k_{O2\to O3}\times c\left(1c\right)\times c\left(OH\right)$$

$$r_{4}=k_{sec.hydr.}\times c\left(1b\right)\times c\left(OH\right)$$

$$r_{5}=k_{prim.hydr.}\times c\left(1b\right)\times c\left(OH\right)$$

$$r_{6}=k_{sec.hydr.}\times c\left(1c\right)\times c\left(OH\right)$$

$$r_{7}=k_{prim.hydr.}\times c\left(1c\right)\times c\left(OH\right)$$

$$r_{8}=k_{O3\to O2}\times c\left(1d\right)\times c\left(OH\right)-k_{O2\to O3}\times c\left(1e\right)\times c\left(OH\right)$$

$$r_{9}=k_{O2\to O6}\times c\left(1e\right)\times c\left(OH\right)$$

$$r_{10}=k_{sec.hydr.}\times c\left(1d\right)\times c\left(OH\right)$$

$$r_{11}=k_{sec.hydr.}\times c\left(1e\right)\times c\left(OH\right)$$

$$r_{12}=k_{prim.hydr.}\times c\left(1f\right)\times c\left(OH\right),$$

where

$$k_{O2\to O6}=k_{1a\to 1b}=k_{1e\to 1f}$$

$$k_{O3\to O2}=k_{1b\to 1c}=k_{1d\to 1e}$$

$$k_{O2\to O3}=k_{1c\to 1b}=k_{1e\to 1d}$$

$$k_{prim.hydr.}=k_{1b\to 1d}=k_{1c\to 1e}=k_{1e\to 1f}$$

$$k_{sec.hydr.}=k_{1a\to 1d}=k_{1a\to 1e}=k_{1b\to 1f}=k_{1c\to 1f}=k_{1d\to 1g}=k_{1e\to 1g}$$

and

$$c\left(OH\right)=\frac{{c\left(OH\right)}_{t}}{10^{-6}},$$

where c(OH)_t_ is the concentration of [OH^−^] at the time t based of pH and 10^−6^ is the concentration of [OH^−^] at pH=8. The mass balances become

$$\frac{{dc}_{1a}}{dt}=-r_{1}-2\times r_{2}$$

$$\frac{{dc}_{1b}}{dt}=r_{1}-r_{3}-r_{4}-r_{5}$$

$$\frac{{dc}_{1c}}{dt}=r_{3}-r_{6}-r_{7}$$

$$\frac{{dc}_{1d}}{dt}=r_{2}+r_{5}-r_{8}-r_{10}$$

$$\frac{{dc}_{1e}}{dt}=r_{2}+r_{7}+r_{8}-r_{9}-r_{11}$$

$$\frac{{dc}_{1f}}{dt}=r_{4}+r_{6}+r_{9}-r_{12}$$

$$\frac{{dc}_{1g}}{dt}=r_{10}+r_{11}+r_{12}.$$

The model for GGM was described in the same way, and the reaction scheme was as follows

$$r_{1}=k_{O3\to O2}\times c\left(A\right)\times c\left(OH\right)-k_{O2\to O3}\times c\left(B\right)\times c\left(OH\right)$$

$$r_{2}=k_{O2\to O6}\times c\left(B\right)\times c\left(OH\right)$$

$$r_{3}=k_{sec.hydr.}\times c\left(A\right)\times c\left(OH\right)$$

$$r_{4}=k_{sec.hydr.}\times c\left(B\right)\times c\left(OH\right)$$

$$r_{5}=k_{prim.hydr.}\times c\left(C\right)\times c\left(OH\right),$$

where

$$c\left(OH\right)=\frac{{c\left(OH\right)}_{t}}{10^{-6}},$$

where c(OH)_t_ is the concentration of [OH^−^]at a certain time t based of pH and 10^−6^ is the concentration of [OH^−^] at pH=8. The mass balances become

$$\frac{{dc}_{A}}{dt}=-r_{1}-r_{3}$$

$$\frac{{dc}_{B}}{dt}=r_{1}-r_{2}-r_{4}$$

$$\frac{{dc}_{C}}{dt}=r_{2}-r_{5}$$

$$\frac{{dc}_{D}}{dt}=r_{3}+r_{4}+r_{5}.$$

The differential equations are solved with the backward difference method as a subtask to the optimizing methods (Simplex and/or Levenberg‐Marquardt) with the software Modest.^[66]^ As objective function, the sum of square function was used:

$$SSQ=\sum_{t}\sum_{i}{\left(c_{i,t,model}-c_{i,t,experiment}\right)}^{2}$$

The fit of model to experimental data was good, and the fit is displayed in the Supporting Information.

**DC‐SIGN lectin expression and purification**: The DNA fragment coding the sequence of DC‐SIGN (ECD, residues 70‐404) was inserted into a pET15b expression vector. The vector was used for the transformation of BL21 (DE3) E. coli competent cells using heat shock method as follow: 42 °C for 90 secs and 5 minutes in ice. The culture was incubated overnight at 37 °C on agar plate in the presence of 100 μg/ml^−1^ of antibiotic ampicillin. One colony harboring the expression vector was selected on the plate and subsequently inoculated overnight in 200 ml of Luria Broth (LB) medium with 100 μg/ml^−1^ of ampicillin. A calculated amount of this culture was then added to 2 l of fresh LB medium containing 100 μg/ml^−1^ of ampicillin in order to obtain a final OD_600_ of 0.1. The 2 l culture was grown at 37 °C and when the OD_600_ has reached 0.6, the protein expression was induced adding 1 mM Isopropyl β‐d‐1‐thio‐galactopyranoside (IPTG). The culture was allowed to grow for 6 hours and then harvested by centrifugation at 4500 rpm for 30 minutes. The pellet obtained was resuspended in 20 ml of lysis buffer (10 mM Tris‐HCl pH 8) and sonicated at 4 °C. The inclusion bodies were isolated by ultracentrifugation (30000 rpm for 30 minutes at 4 °C) and the pellet obtained was dissolved in Tris‐HCl 100 mM (pH 8) with 6 M urea. The sample was incubated overnight with rotation at 4 °C. The mixture was centrifuged at 35000 rpm for 2 hours at 4 °C and the supernatant containing the soluble protein was collected. The protein was refolded through subsequent dialysis against 100 mM Tris‐HCl, 100 mM NaCl, 10 mM CaCl_2_, pH 8 containing 4 M, 2 M and at the end no urea. Unfolded residual proteins were eliminated by ultracentrifugation (30000 rpm for 30 minutes at 4 °C) and the supernatant containing folded DC‐SIGN was purified using affinity chromatography of mannose‐Sepharose (Sigma‐Aldrich). The loading buffer used was 20 mM Tris‐HCl, 150 mM NaCl, 10 mM CaCl_2_, pH 8. The sample was eluted with the 20 mM Tris‐HCl, 150 mM NaCl and 10 mM EDTA. A subsequent additional purification by size exclusion chromatography was performed using HiLoad 26/600 Superdex 200 pg column eluting with 25 mM Tris‐HCl, 150 mM NaCl, 1 mM EDTA, pH 8. The fractions containing the purified protein were concentrated and the buffer was changed to 25 mM Tris, 4 mM CaCl_2,_ 150 mM NaCl at pH 8.0. The presence of the protein was checked by 4–12 % SDS‐PAGE. The concentration of the protein was quantified using NanoDrop UV‐Vis spectrophotometer (absorbance at 280 nm). For STD‐NMR experiments, the buffer was changed to 25 mM Tris‐d11,150 mM NaCl, 4 mM CaCl_2_ in D_2_O. The tetrameric state of the lectin was confirmed by LC‐MS and by TEM (Jeol JEM‐1230, Tokyo, Japan) using negative staining.

**NMR experiments with DC‐SIGN ECD**: The NMR experiments were acquired using Bruker AVANCE 2 600 MHz spectrometer equipped with a 5 mm QCI cryo‐probe. The samples were prepared in 350 μl of total volume and transferred in 5 mm shigemi NMR tubes. DC‐SIGN was used at a concentration of 5 μM of tetramer in 25 mM Tris‐d11,150 mM NaCl, 4 mM CaCl_2_, 2 mM DTT‐d10 in D_2_O. The standard ratio lectin/ligand was set at 1 : 100 for all the ligands tested (**1** **a**, **1** **b** and **2**).

*STD‐NMR experiments*: The STD sequence *stddiffesgp* was selected from Bruker library and include excitation sculpting. The temperature during the acquisition was 298 K. The off‐resonance frequency of the experiment was set at δ 100 ppm and the on‐resonance at δ 0.8 ppm. 2 sec of relaxation delay and 2592 scans were used. The STD spectra presented were obtained by subtracting the on‐resonance spectrum from the off‐resonance spectrum. The STD Amplification Factor (STD‐AF) was calculated on the basis of the comparison between the signals of the STD spectrum and those of the off‐resonance spectrum. The STD% (reported in the epitope map) was calculated by normalization of the whole set of STD factors against the highest value for each ligand (100 % of STD effect). The same STD experiment was repeated for each sample after 1 month. During the month, the samples were conserved at 4 °C and checked for presence of precipitate before the use.

^*1*^ *H‐^13^C HSQC experiments*: The ^1^H‐^13^C HSQC experiments were performed using standard Bruker pulse sequence, with 416 (T1) and 1024 (T2) complex data points and 16 scans.

## Conflict of interest

The authors declare no conflict of interest.

## Supporting information

As a service to our authors and readers, this journal provides supporting information supplied by the authors. Such materials are peer reviewed and may be re‐organized for online delivery, but are not copy‐edited or typeset. Technical support issues arising from supporting information (other than missing files) should be addressed to the authors.

Supporting Information
