# Supplementary material for: Kinetic Studies of Acetyl Group Migration between the Saccharide Units in an Oligomannoside Trisaccharide Model Compound and a Native Galactoglucomannan Polysaccharide
Source: Chembiochem. 2021 Sep 2;22(20):2986–95. doi: 10.1002/cbic.202100374 (PMC8597014; doi:10.1002/cbic.202100374)

ChemBioChem

Supporting Information

Kinetic Studies of Acetyl Group Migration between the Saccharide Units in an Oligomannoside Trisaccharide Model Compound and a Native Galactoglucomannan Polysaccharide

Robert Lassfolk, Sara Bertuzzi, Ana Ardá, Johan Wärnå, Jesús Jiménez-Barbero, and Reko Leino*

TABLE OF CONTENTS

General information S3

Synthesis of model compounds S3

Synthesis of compound **1b** S3

Synthesis of comound **2** S4

Migration study S5

pH profiles S5

Kinetic modeling S5

Kinetic modeling of the trisaccharide model compounds S5

Starting from **1a** S5

Starting from **1b** S5

Kinetic modeling of GGM S7

2 mg/ml S7

20 mg/ml S7

10 mg/ml S7

Experimental procedures S8

References S16

NMR spectra S18

General information

For following the migration process and for identification and characterization of the new compounds, a Bruker Avance-III spectrometer operating at 500.20 MHz (^{1}H) and 125.78 MHz (^{13}C) equipped with a Prodigy BBO CryoProbe was used. The characterization was performed using a standard set of 1D and 2D NMR spectroscopic techniques: ^{1}H, ^{13}C, 1D-TOCSY, DQF-COSY, Multiplicity edited HSQC (CH and CH_3 positive, CH_2 negative, both coupled and decoupled), and HMBC. The reported signals are referenced to an internal standard (TMS δ_H = 0.0 ppm, δ_C = 0.0 ppm) or residual solvent signal (MeOH δ_H = 3.31 ppm, δ_C = 49.00 ppm, $CDCl_3$ δ_H = 7.26 ppm, δ_C = 77.16 ppm). Chemical shifts are reported with two decimals for ^{1}H and one decimal for ^{13}C, where this is not sufficient for distinguishing two signals an additional decimal is given. Coupling constants are reported in Hz with one decimal and mentioned only the first time they are encountered. Accurate coupling constants and shifts were extracted from the ^{1}H spectra using the NMR simulation software ChemAdder/SpinAdder[1]. HRMS was recorded on a Bruker daltonics micro-ToF with ESI in positive mode as ionization source. TLC analysis was performed on Merck silica gel 60 F254 plates and the spots were visualized with UV light and charring with H_2SO_4/MeOH (1:4) and heating. All reactions were monitored by TLC. Column chromatography was carried out using silica gel 60 (0.040 – 0.060 mm) as stationary phase and as eluents hexane:EtOAc or toluene:EtOAc were used. All chemicals were purchased from Sigma-Aldrich and used as such. Dry dichloromethane was obtained by distillation from a suspension of CaH_2 under argon. Dry MeOH and DMF were purchased and used as such. Reactions sensitive towards moisture and air were carried out under argon atmosphere.

Synthesis of model compounds

Compound **1a** was synthesized according to an earlier reported method.[2]

Synthesis of compound 1b

The synthesis of **1b** started by making building block **4** from **3**.[3] The selective ring opening of **3** was done using I_2 and $NaBH_3CN$ in acetonitrile.[4] The yield of **5** was fair using this selective ring opening method. The rest of the synthesis followed the same synthesis pattern as has previously been done for **1a**.[2] A β-mannosylation according to Crich protocol[5,6]with donor **5** and acceptor **4** yielded **6** in a fair yield. The removal of PMB groups and the following benzoylation gave a good yield of **7**. The selective ring opening using $BF_3{\cdot}OEt_2$ and Et_3SiH also gave a good yield of **8**.

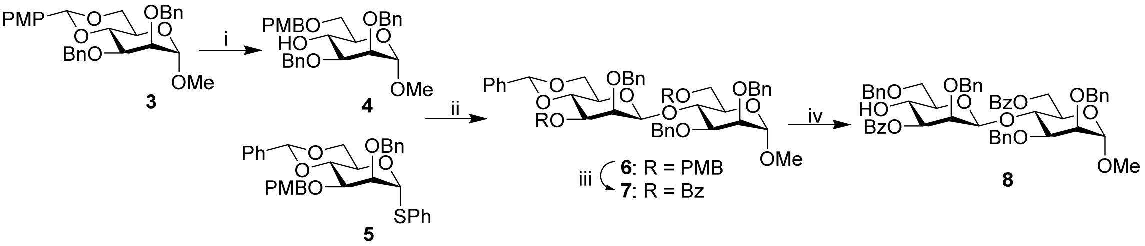

Scheme S1. Synthesis of intermediate **8** from **3** and **5**. (i) I_2, $NaCNBH_3$, AcCN, 0 °C, 0.5 h, 46%; (ii) 1) **5**, BSP, TTBP, Tf_2O, CH_2Cl_2, –60 °C, 0.5 h, 2) –78 °C, **4**, 2 h (45%); (iii) 1) DDQ, CH_2Cl_2:MeOH:H_2O 92:4:4, 3 h, 2) BzCl, pyridine, 1 h, 54%; (iv) Et_3SiH, $BF_3{\cdot}OEt_2$, CH_2Cl_2, 0 °C→r.t., 2 h, 76%.

A β-mannosylation with donor **9** and acceptor **8** yielded **10** in a fair yield. This was then followed by the removal of the benzoyl groups under Zemplén conditions[7] with NaOMe and an acetylation using Ac_2O in pyridine, which gave **11** in fair a yield. The removal of benzyl and benzylidene groups was done using hydrogenolysis with Pd/C in MeOH:AcOH 2:1 to give **1b** in an excellent yield.

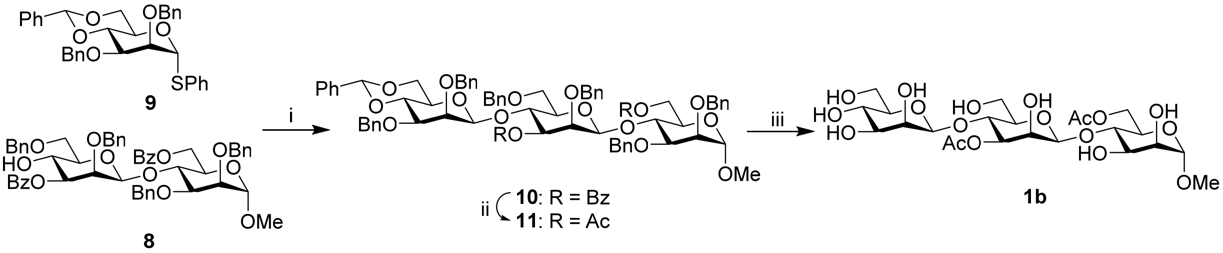

Scheme S2. Synthesis of target compound **1b**. (i) 1) **9**, BSP, TTBP, Tf_2O, CH_2Cl_2, –60 °C, 0.5 h, 2) –78 °C, **8**, 2 h (60%); (ii) 1) NaOMe, MeOH, 24 h, 2) Ac_2O, pyridine, 45 h, 52%; (iii) Pd/C, MeOH:AcOH 2:1, 4 h (91%).

Synthesis of compound 2

The synthesis of trisaccharide **2** started from compound **12**,[8] which was selectively acetylated on the free O6 position using AcCl in pyridine. Then a β-mannosylation was performed with donor **9** and acceptor **13** to get **14**. This glycosylation did not give a good yield, probably due to the instability of the acetyl group. From there hydrogenolysis gave the target compound **2** in excellent yield.

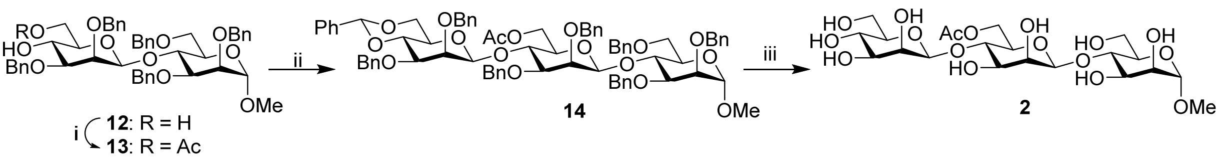

Scheme S3. Synthesis of model compound **2**. (i) AcCl, pyridine, 0 °C→r.t., 76 h, 85%; (ii)) 1) **9**, BSP, TTBP, Tf_2O, CH_2Cl_2, –60 °C, 0.5 h, 2) –78 °C, **13**, 2 h (26%); (iii) Pd/C, MeOH:AcOH 2:1, 4 h (91%).

Migration study

pH profiles

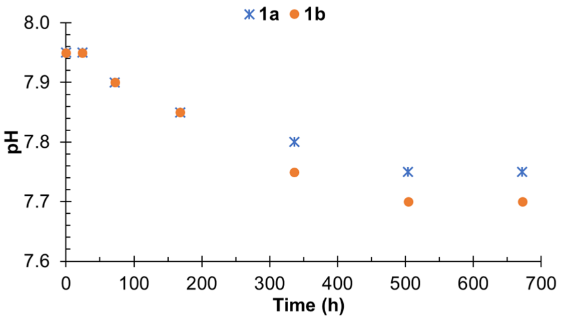

Figure S1. The pH profile starting from **1a** and **1b**.

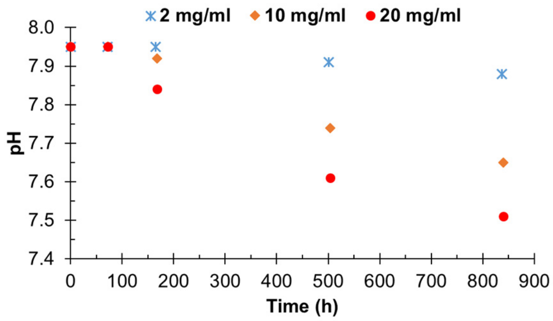

Figure S2. The pH profile of the migration study in GGM.

Kinetic modeling

Kinetic modeling of the trisaccharide model compounds

In figures S3-S16 the kinetic model and the experimental data is shown for migration in the trisaccharide model compounds. The kinetic model explains the experimental data with 99.20%.

*Starting from **1a***

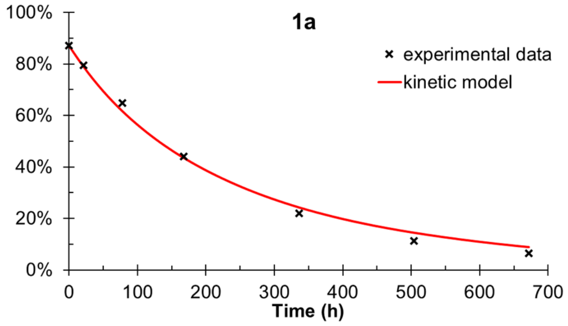

Figure S3. Experimental data vs the kinetic model of **1a** starting from **1a** in buffered H_2O at 25 °C and starting pH = 8.

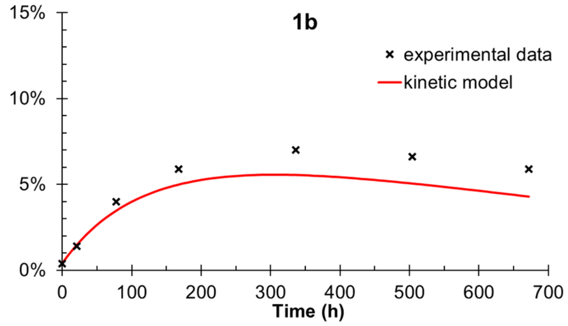

Figure S5. Experimental data vs the kinetic model of **1b** starting from **1a** in buffered H_2O at 25 °C and starting pH = 8.

*Starting from **1b***

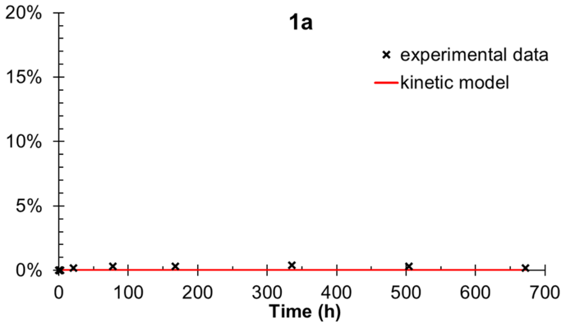

Figure S4. Experimental data vs the kinetic model of **1a** starting from **1b** in buffered H_2O at 25 °C and starting pH = 8.

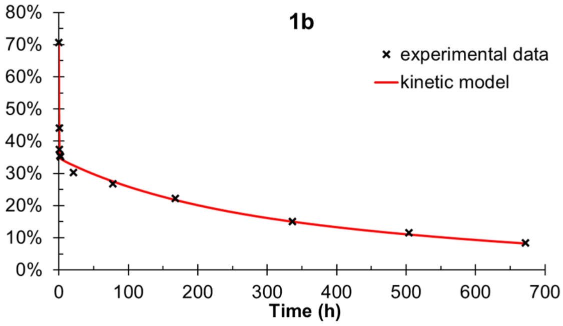

Figure S6. Experimental data vs the kinetic model of **1b** starting from **1b** in buffered H_2O at 25 °C and starting pH = 8.

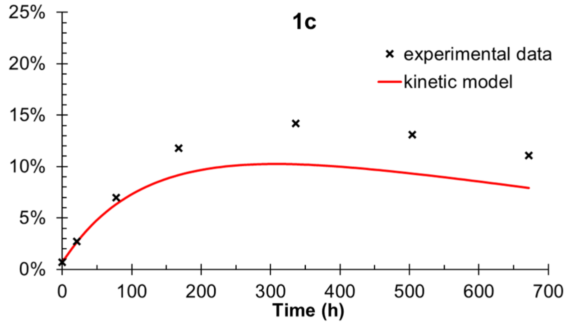

Figure S7. Experimental data vs the kinetic model of **1c** starting from **1a** in buffered H_2O at 25 °C and starting pH = 8.

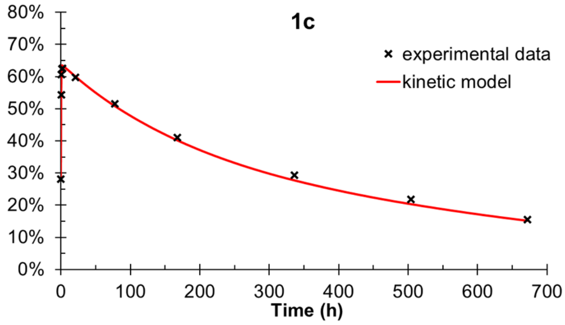

Figure S8. Experimental data vs the kinetic model of **1c** starting from **1b** in buffered H_2O at 25 °C and starting pH = 8.

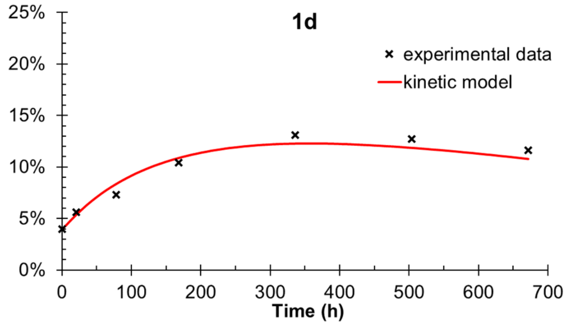

Figure S9. Experimental data vs the kinetic model of **1d** starting from **1a** in buffered H_2O at 25 °C and starting pH = 8.

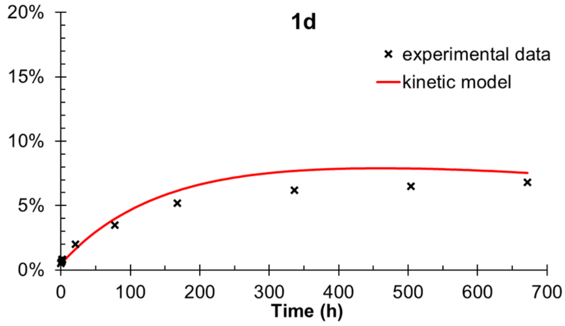

Figure S10. Experimental data vs the kinetic model of **1d** starting from **1b** in buffered H_2O at 25 °C and starting pH = 8.

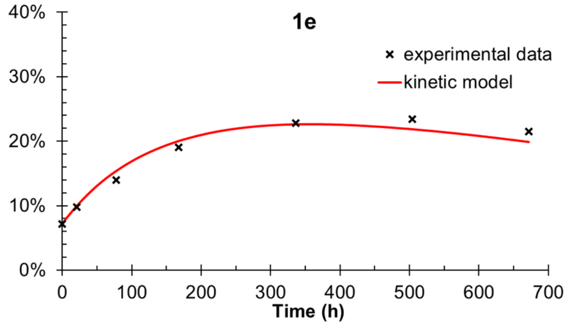

Figure S11. Experimental data vs the kinetic model of **1e** starting from **1a** in buffered H_2O at 25 °C and starting pH = 8.

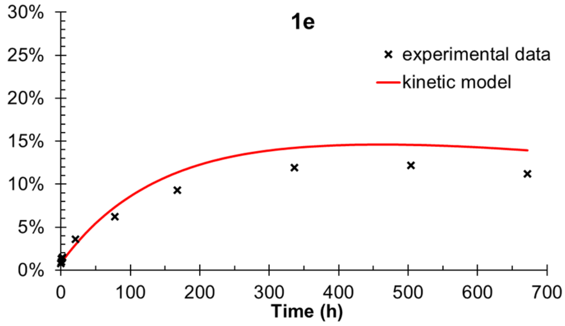

Figure S12. Experimental data vs the kinetic model of **1e** starting from **1b** in buffered H_2O at 25 °C and starting pH = 8.

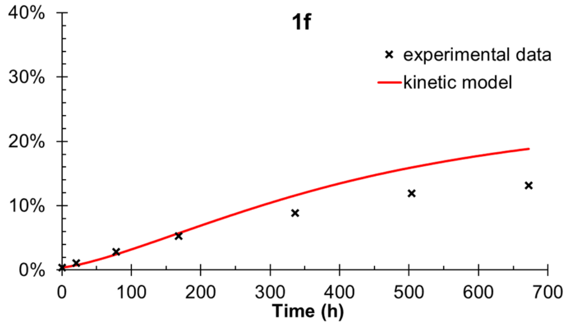

Figure S13. Experimental data vs the kinetic model of **1f** starting from **1a** in buffered H_2O at 25 °C and starting pH = 8.

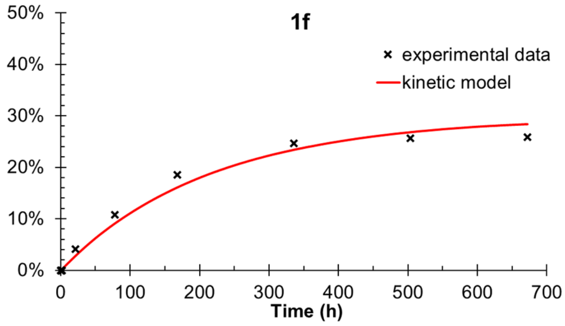

Figure S14. Experimental data vs the kinetic model of **1f** starting from **1b** in buffered H_2O at 25 °C and starting pH = 8.

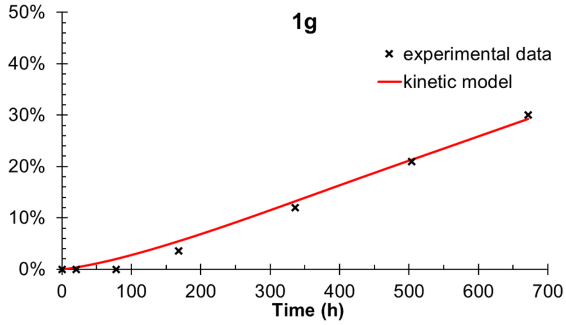

Figure S15. Experimental data vs the kinetic model of **1g** starting from **1a** in buffered H_2O at 25 °C and starting pH = 8.

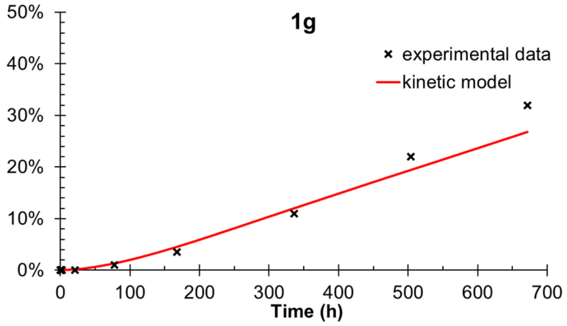

Figure S16. Experimental data vs the kinetic model of **1g** starting from **1b** in buffered H_2O at 25 °C and starting pH = 8.

Kinetic modeling of GGM

In figures S17-S19 the kinetic model and the experimental data is shown for migration in the GGM.

2 mg/ml

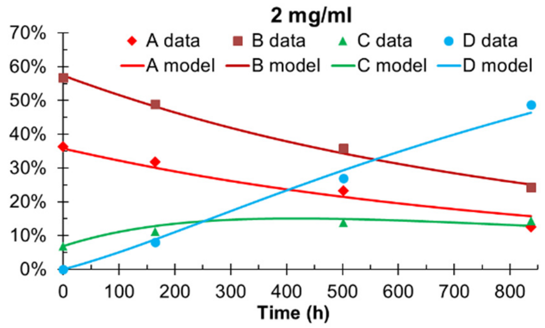

Figure S17. Experimental data vs the kinetic model of GGM with a concentration of 2 mg/ml in buffered H_2O at 25 °C and starting pH = 8 (degree of explanation 98.25%).

10 mg/ml

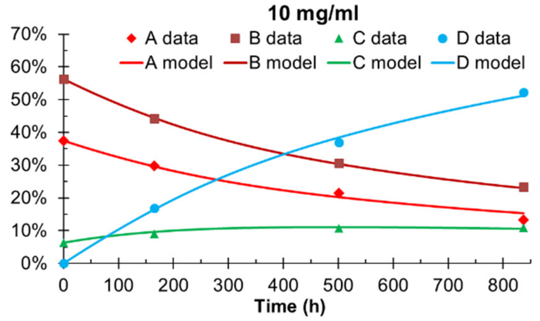

Figure S18. Experimental data vs the kinetic model of GGM with a concentration of 10 mg/ml in buffered H_2O at 25 °C starting and pH = 8 (degree of explanation 99.55%).

20 mg/ml

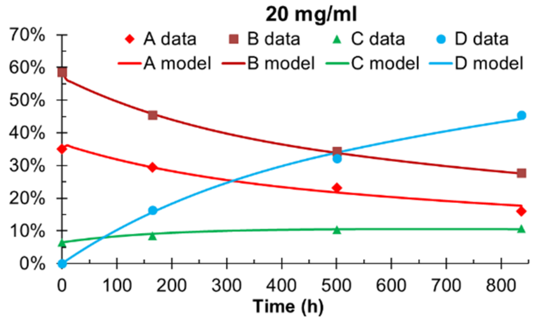

Figure S19. Experimental data vs the kinetic model of GGM with a concentration of 20 mg/ml in buffered H_2O at 25 °C and starting pH= 8 (degree of explanation 99.43%).

Experimental procedures

Standard reaction procedure for β-mannosylation. To a solution of the donor (1 equivalent) in dry CH_2Cl_2 (1 ml/50 mg donor) at –60 °C (acetone + dry ice), 4 Å molecular sieves, BSP (1.2 equivalents), TTBP (1.5 equivalents) and Tf_2O (1.3 equivalents) were added. The reaction mixture was stirred for 0.5 h (until activation of the donor was complete). The reaction mixture was then cooled to -78 °C and the acceptor (0.7 equivalents), dissolved in CH_2Cl_2 (1 ml/70 mg acceptor), was added dropwise. The reaction was stirred at –78 °C for 1 – 3 h (until completion) and then quenched with Et_3N and stirred for 0.5 h. The reaction mixture was then warmed to room temperature and diluted with CH_2Cl_2. Next, the mixture was washed with saturated $NaHCO_3$ solution and saturated NaCl solution. The organic phase was dried over Na_2SO_4 and the solvent evaporated. The product was purified using column chromatography.

Standard reaction procedures for hydrogenolysis of benzyl- and benzylidene protecting groups. To a solution of the substrate in MeOH:AcOH 2:1 (1.5 ml/20 mg substrate) was Pd/C 10% w/w (2 weight equivalents) was added. The reaction mixture was stirred under 3 bar H_2-gas in an autoclave reactor for 4 h, after which the mixture was filtered and the solvent evaporated.

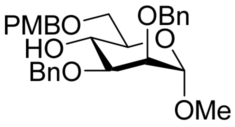

Methyl 2,3-di-*O*-benzyl-6-*O*-*p*-methoxybenzyl-α-D-mannopyranoside (4). To a solution of **3** (1320 mg, 1 equivalent) in acetonitrile (12 ml) was 3 Å molecular sieves added under stirring. After 15 min $NaBH_3CN$ was added and the reaction mixture was cooled to 0 °C. I_2 was dissolved in acetonitrile (60 ml) and added dropwise to the reaction mixture. After 30 min the reaction was diluted with CH_2Cl_2 (100 ml) and the mixture was filtered through celite. The solution was the washed with 10% Na_2CO_3 solution (2 × 80 ml) and saturated NaCl solution (80 ml). The organic phase was dried with Na_2SO_4 and the solvent was evaporated. The crude product was purified by column chromatography (hexane:EtOAc 2:1) to provide **4** as a clear oil. Yield: 620 mg (46%), R_f = 0.28.

^{1}H NMR (500.20 MHz, $CDCl_3$, 25 °C): δ = 7.38 – 6.82 (m, 14 H, aromatic H), 4.77 (d, 1 H, $J_{H\text{-}1,H\text{-}2}$ = 1.8 Hz, H-1), 4.69 (d, 1 H, *J* = -12.3, 2-OC*H₂*Ph), 4.65 (d, 1 H, 2-OC*H₂*Ph), 4.59 (d, 1 H, *J* = -11.8, 3-OC*H₂*Ph), 4.56 (d, 1 H, *J* = -11.6, 6-OC*H₂*Ph-*p*-OCH₃), 4.52 (d, 1 H, 6-OC*H₂*Ph- *p*-OCH₃), 4.50 (d, 1 H, 3-OC*H₂*Ph), 4.03 (ddd, 1 H, $J_{H\text{-}3,H\text{-}4}$ = 9.5 Hz, $J_{H\text{-}4,H\text{-}5}$ = 9.4 Hz, $J_{H\text{-}4,4\text{-}OH}$ = 2.1 Hz, H-4), 3.79 (s, 3 H, 6-OCH₂Ph-*p*-OC*H₃*), 3.78 (dd, 1 H, $J_{H\text{-}2,H\text{-}3}$ = 3.1 Hz, H-2), 3.76 (dd, 1 H, $J_{H\text{-}5,H\text{-}6a}$ = 3.5 Hz, $J_{H\text{-}6a,H\text{-}6b}$ = – 10.4 Hz, H-6a), 3.73 (dd, 1 H, $J_{H\text{-}5,H\text{-}6b}$ = 5.8 Hz, H-6b), 3.70 (ddd, 1 H, H-5), 3.69 (dd, 1 H, H-3), 3.34 (s, 3 H, 1-OC*H₃*), 2.52 (d, 1H, 4-OH) ppm.

^{13}C NMR (125.78 MHz, $CDCl_3$, 25 °C): δ = 138.2 – 113.7 (aromatic C), 99.1 (C-1), 79.6 (C-3), 73.8 (C-2), 73.2 (6-*C*H_2Ph-*p*-OCH_3), 72.6 (2-*C*H_2Ph), 71.8 (3-*C*H_2Ph), 71.2 (C-5), 70.2 (C-6), 68.0 (C-4), 55.3 (6-CH_2Ph-*p*-O*C*H_3), 54.9 (1-O*C*H_3) ppm.

HRMS: calculated for $C_{29}H_{34}O_7Na$, $[M + Na]^+$ 517.2197, measured 517.2218.

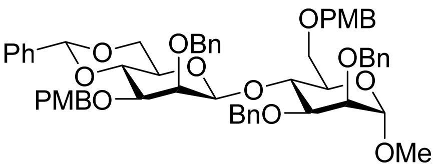

Methyl *O*-(4,6-*O*-benzylidene-2-*O*-benzyl-3-*O*-*p*-methoxybenzyl-β-D-mannopyranosyl)-(1→4)-2,3-di-*O*-benzyl-6-*O*-*p*-methoxybenzyl-α-D-mannopyranoside (6). Prepared from donor **5** (940 mg, 1.65 mmol) and acceptor **4** (570 mg, 1.16 mmol) according to the standard reaction procedure for β-mannosylation. The crude product was purified by column chromatography (toluene:EtOAc 7:1) to provide **6** as a clear oil. Yield: 700 mg (45%), R_f = 0.40.

^1H NMR (500.20 MHz, $CDCl_3$, 25 °C): δ = 7.48 – 6.79 (m, 28 H, aromatic H), 5.50 (s, 1 H, 4′,6′-OC*H*Ph), 4.84 (d, 1 H, *J* = –12.0 Hz, 3-OCH_2Ph), 4.761 (d, 1 H, *J* = –12.1 Hz, 2′-OCH_2Ph), 4.759 (d, 1 H, 2′-OCH_2Ph), 4.74 (d, 1 H, $J_{H\text{-}1,H\text{-}2}$ = 2.3 Hz, H-1), 4.73, (d, 1 H, *J* = –12.3 Hz, 2-OCH_2Ph), 4.67 (d, 1 H, 2-OCH_2Ph), 4.62 (d, 1 H, *J* = –12.0 Hz, 3′-OCH_2Ph), 4.60 (d, 1 H, *J* = –12.0 Hz, 6-OCH_2Ph), 4.56 (d, 1 H, 3-OCH_2Ph), 4.49 (d, 1 H, 3′-OCH_2Ph), 4.48 (d, 1 H, $J_{H\text{-}1',H\text{-}'2}$ = 1.0 Hz, H-1′), 4.44 (d, 1 H, 6-OCH_2Ph), 4.19 (dd, 1 H, $J_{H\text{-}3,H\text{-}4}$ = 8.5 Hz, $J_{H\text{-}4,H\text{-}5}$ = 9.6 Hz, H-4), 4.044 (dd, 1 H, $J_{H\text{-}3',H\text{-}4'}$ = 9.7 Hz, $J_{H\text{-}4',H\text{-}5'}$ = 9.6 Hz, H-4′), 4.039 (dd, 1 H, $J_{H\text{-}5',H\text{-}6'a}$ = 4.8 Hz, $J_{H\text{-}6'a,H\text{-}6'b}$ = –10.5 Hz, H-6′a), 3.84 (dd, 1 H, $J_{H\text{-}2,H\text{-}3}$ = 3.2 Hz, H-3), 3.78 (s, 3 H, OCH_2Ph-*p*-OCH_3), 3.74 (dd, 1 H, H-2), 3.70 (dd, 1 H, $J_{H\text{-}2',H\text{-}3'}$ = 2.8 Hz, H-2′), 3.69 (s, 3 H, OCH_2Ph-*p*-OCH_3), 3.65 (ddd, 1 H, $J_{H\text{-}5,H\text{-}6a}$ = 4.9 Hz, $J_{H\text{-}5,H\text{-}6b}$ = 2.3 Hz, H-5), 3.6105 (dd, 1 H, $J_{H\text{-}5',H\text{-}6'b}$ = 9.6 Hz, H-6′b), 3.6101 (dd, 1 H, $J_{H\text{-}6a,H\text{-}6b}$ = –11.8 Hz, H-6a), 3.60 (dd, 1 H, H-6a), 3.37 (dd, 1 H, H-3′), 3.35 (s, 3 H, 1-OCH_3), 3.07 (ddd, 1 H, H-5′) ppm.

^{13}C NMR (125.8 MHz, $CDCl_3$, 25 °C): δ = 159.3 – 113.8 (aromatic C), 102.2 (C-1′), 101.4 (4′6′-O*C*HPh), 99.6 (C-1), 78.7 (C-4′), 78.24 (C-3′), 78.20 (C-3), 77.3 (C-2′), 76.3 (C-4), 75.9 (C-2), 75.0 (2′-O*C*H_2Ph), 73.2 (6-O*C*H_2Ph-*p*-OCH_3), 72.9 (2-O*C*H_2Ph), 72.8 (3-O*C*H_2Ph), 72.3 (3′-O*C*H_2Ph-*p*-OCH_3), 71.3 (C-5), 69.0 (C-6), 68.7 (C-6′), 67.4 (C-5′), 55.4 (OCH_2Ph-O*C*H_3), 55.3(OCH_2Ph-O*C*H_3), 55.0 (1-O*C*H_3) ppm.

$^1J_{C\text{-}1,H\text{-}1}$ = 165 Hz (α), $^1J_{C\text{-}1',H\text{-}1'}$ = 157 Hz (β).

HRMS: calculated for $C_{57}H_{62}O_{13}Na$, $[M + Na]^+$ 977.4083, measured 977.4074.

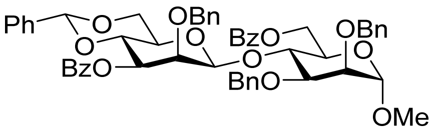

Methyl *O*-(3-*O*-benzoyl-2-O-benzyl-4,6-*O*-benzylidene-β-D-mannopyranosyl)-(1→4)-2,3-di-*O*-benzyl-6-*O*-benzoyl-α-D-mannopyranoside (7). To a solution of **6** (644 mg, 1 equivalent) in CH_2Cl_2:MeOH:H_2O 92:4:4 (22.8 ml) at 0 °C was DDQ (460 mg, 3 equivalents) dissolved in CH_2Cl_2 (15 ml) added. The mixture was slowly brought to RT over 3 h. The reaction mixture was diluted with CH_2Cl_2 (150 ml) and washed with saturated $NaHCO_3$ solution (150 ml), water (100 ml), and saturated NaCl solution (100 ml). The organic phase was dried over Na_2SO_4, filtered and the solvent was evaporated. The product was filtered through silica gel (hexane:EtOAc 3:1 → 1:1). The product (300 mg, 1 equivalent) was dissolved in pyridine (3 ml) and BzCl (170 µl, 3.6 equivalents) was added. After 1 h, the reaction mixture was quenched with DMAPA and diluted with CH_2Cl_2 (100 ml) and washed with saturated $NaHCO_3$ solution (80 ml), 1 M HCl (80 ml) and saturated NaCl solution (80 ml). The organic phase was dried over Na_2SO_4, filtered and evaporated. The crude product was purified by column chromatography (hexane:EtOAc 2:1) to provide **7** as a white foam. Yield: 320 mg (54%), R_f = 0.41.

^{1}H NMR (500.20 MHz, $CDCl_3$, 25 °C): δ = 8.07 – 7.11 (m, 30 H, aromatic H), 5.49 (s, 1 H, 4′,6′-OC*H*Ph), 5.16 (dd, 1 H, $J_{H-3',H-4'}$ = 10.4 Hz, H-3′), 4.87 (d, 1 H, $J_{H-1',H-2'}$ = 1.0 Hz, H-1′), 4.83 (d, 1 H, *J* = –11.8 Hz, 2′-OCH_2Ph), 4.80 (d, 1 H, *J* = –11.7 Hz, 3-OCH_2Ph), 4.79 (d, 1 H, $J_{H-1,H-2}$ = 2.4 Hz, H-1), 4.73 (d, 1 H, *J* = –12.1 Hz, 2-OCH_2Ph), 4.66 (d, 1 H, 2-OCH_2Ph), 4.652 (d, 1 H, 3-OCH_2Ph), 4.648 (d, 1 H, 2′-OCH_2Ph), 4.59 (dd, 1 H, $J_{H-5,H-6a}$ = 2.3 Hz, $J_{H-6a,H-6b}$ = –11.9 Hz, H-6a), 4.53 (dd, 1 H, $J_{H-5,H-6b}$ = 4.9 Hz, H-6b), 4.32 (dd, 1 H, $J_{H-3,H-4}$ = 8.6 Hz, $J_{H-4,H-5}$ = 9.9 Hz, H-4), 4.20 (dd, 1 H, $J_{H-4',H-5'}$ = 9.4 Hz, H-4′), 4.19 (dd, 1 H, $J_{H-2',H-3'}$ = 3.3 Hz, H-2′), 4.05 (dd, 1 H, $J_{H-5'a,H-6'a}$ = 4.8 Hz, $J_{H-6'a,H-6'b}$ = –10.6 Hz, H-6′a), 3.99 (dd, 1 H, $J_{H-2,H-3}$ = 3.2 Hz, H-3), 3.92 (ddd, 1 H, H-5), 3.82 (dd, 1 H, H-2), 3.69 (dd, 1 H, $J_{H-5',H-6'b}$ = 9.9 Hz, H-6′b), 3.38 (s, 3 H, 1-OCH_3), 3.22 (ddd, 1 H, H-5′) ppm.

^{13}C NMR (125.78 MHz, $CDCl_3$, 25 °C): δ = 166.3 (6-O*C*OPh), 165.7 (3′-O*C*OPh), 138.7 – 126.1 (aromatic C), 101.6 (4′6′-O*C*HPh), 101.2 (C-1′), 99.1 (C-1), 77.5 (C-3), 77.3 (C-2′), 75.9 (C-4′), 75.8 (2′-O*C*H_2Ph), 75.7(C-4), 75.3 (C-2), 72.9 (2-O*C*H_2Ph), 72.8 (C-3′), 72.2 (3-O*C*H_2Ph), 69.8 (C-5), 68.5 (C-6′), 67.3 (C-5′), 63.7 (C-6), 55.0 (1-O*C*H_3) ppm.

HRMS: calculated for $C_{55}H_{54}O_{13}Na$, $[M + Na]^+$ 945.3457, measured 945.3482.

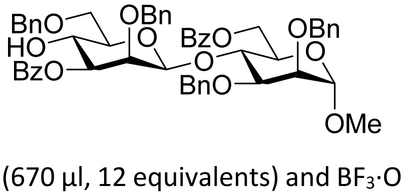

Methyl *O*-(3-*O*-benzoyl-2,6-di-*O*-benzyl-β-D-mannopyranosyl)-(1→4)-6-*O*-benzoyl-2,3-di-*O*-benzyl-α-D-mannopyranoside (8). To a solution of **7** (320 mg, 1 equivalent) in CH_2Cl_2 (3 ml) at 0 °C, Et_3SiH (670 µl, 12 equivalents) and BF_3·OEt_2 (70 µl, 1.6 equivalents) were added and the reaction mixture was slowly brought to RT over 2 h. The reaction mixture was then diluted with CH_2Cl_2 (100 ml) and washed

with saturated $NaHCO_3$ solution (100 ml) and saturated NaCl solution (100 ml). The organic phase was dried over Na_2SO_4, filtered and evaporated. The crude product was purified by column chromatography (hexane:EtOAc 2:1) to provide **8** as a white foam. Yield: 245 mg (76%), R_f = 0.30.

^{1}H NMR (500.20 MHz, $CDCl_3$, 25 °C): δ = 8.06 – 7.11 (m, 30 H, aromatic H), 4.96 (dd, 1 H, $J_{H-2',H-3'}$ = 3.1 Hz, $J_{H-3',H-4'}$ = 9.8 Hz, H-3′), 4.84 (d, 1 H, *J* = –11.9 Hz, 2′-OC*H*$_2$Ph), 4.81 (d, 1 H, $J_{H-1',H-2'}$ = 0.7 Hz, H-1′), 4.79 (d, 1 H, *J* = –12.1 Hz, 3-OC*H*$_2$Ph), 4.78 (d, 1 H, $J_{H-1,H-2}$ = 2.3 Hz, H-1), 4.73 (d, 1 H, *J* = –12.2 Hz, 2-OC*H*$_2$Ph), 4.66 (d, 1 H, 2′-OC*H*$_2$Ph), 4.65 (d, 1 H, 2-OC*H*$_2$Ph), 4.62 (d, 1 H, 3-OC*H*$_2$Ph), 4.60 (dd, 1 H, $J_{H-5,H-6a}$ = 2.3 Hz, $J_{H-6a,H-6b}$ = –12.1 Hz, H-6a), 4.53 (dd, 1 H, $J_{H-5,H-6b}$ = 4.9 Hz, H-6b), 4.45 (d, 1 H, *J* = –11.8 Hz, 6′-OC*H*$_2$Ph), 4.42 (d, 1 H, 6′-OC*H*$_2$Ph), 4.33 (dd, 1 H, $J_{H-3,H-4}$ = 8.7 Hz, $J_{H-4,H-5}$ = 9.7 Hz, H-4), 4.16 (ddd, 1 H, $J_{H-4',H-5'}$ = 9.3 Hz, $J_{H-4',4'-OH}$ = 2.9 Hz, H-4′), 4.04 (dd, 1 H, H-2′), 4.00 (dd, 1 H, $J_{H-2,H-3}$ = 3.1 Hz, H-3), 3.96 (ddd, 1 H, H-5), 3.81 (dd, 1 H, H-2), 3.63 (dd, 1 H, $J_{H-5'a,H-6'a}$ = 5.6 Hz, $J_{H-6'a,H-6'b}$ = –10.2 Hz, H-6′a), 3.61 (dd, 1 H, $J_{H-5',H-6'b}$ = 5.1 Hz, H-6′b), 3.35 (s, 3 H, 1-OC*H$_3$*), 3.28 (ddd, 1 H, H-5′), 2.81 (d, 1 H, 4-OH) ppm.

^{13}C NMR (125.78 MHz, $CDCl_3$, 25 °C): δ = 166.3 (6-O*C*OPh), 166.2 (3′-O*C*OPh), 138.8 – 127.3 (aromatic C), 100.5 (C-1′), 99.1 (C-1), 77.5 (C-3), 76.6 (C-2′), 76.2 (C-3′), 75.22 (2′-O*C*H$_2$Ph), 75.16 (C-2, C-4), 74.0 (C-5′), 73.8 (6′-O*C*H$_2$Ph), 72.8 (2-O*C*H$_2$Ph), 72.0 (3-O*C*H$_2$Ph), 71.3 (C-6′), 69.8 (C-5), 68.2 (C-4′), 63.8 (C-6), 54.9 (1-O*C*H$_3$) ppm.

HRMS: calculated for $C_{55}H_{56}O_{13}Na$, $[M + Na]^+$ 947.3613, measured: 947.3655.

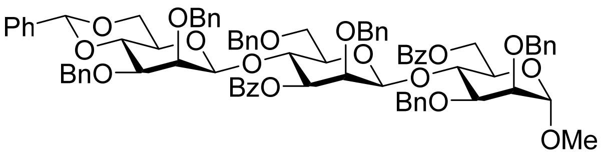

Methyl *O*-(2,3-di-*O*-benzyl-4,6-*O*-benzylidene-β-D-mannopyranosyl)-(1→4)-*O*-(3-*O*-benzoyl-2,6-di-*O*-benzyl-β-D-mannopyranosyl)-(1→4)-6-*O*-benzoyl-2,3-di-*O*-benzyl-α-D-mannopyranoside (10). Prepared from donor **9** (200 mg, 0.37 mmol) and acceptor **8** (235 mg, 0.25 mmol) according to the standard reaction procedure for β-mannosylation. The crude product was purified by column chromatography (toulene:EtOAc 10:1) to provide **10** as a white foam. Yield: 208 mg (60%), R_f = 0.21.

^{1}H NMR (500.20 MHz, $CDCl_3$, 25 °C): δ = 8.07 – 7.10 (m, 45 H, aromatic H), 5.36 (s, 1 H, 4′′,6′′-OC*H*Ph), 5.12 (dd, 1 H, $J_{H-2',H-3'}$ = 3.3 Hz, $J_{H-3',H-4'}$ = 10.0, Hz, H-3′), 4.83 (d, 1 H, *J* = –11.8 Hz, 2′-OC*H*$_2$Ph), 4.81 (d, 1 H, *J* = –12.0 Hz, 3-OC*H*$_2$Ph), 4.791 (d, 1 H, $J_{H-1',H-2'}$ = 0.9 Hz, H-1′), 4.785 (d, 1 H, $J_{H-1,H-2}$ = 2.1 Hz, H-1), 4.71 (d, 1 H, *J* = –11.9 Hz, 2′′-OC*H*$_2$Ph), 4.70 (d, 1 H, *J* = –12.0 Hz, 2-OC*H*$_2$Ph), 4.67 (d, 1 H, 2′-OC*H*$_2$Ph), 4.66 (d, 1 H, 2-OC*H*$_2$Ph), 4.65 (d, 1 H, 3-OC*H*$_2$Ph), 4.62 (dd, 1 H, $J_{H-5,H-6a}$ = 2.3 Hz, $J_{H-6a,H-6b}$ = –12.0 Hz, H-6a), 4.603 (d, 1 H, *J* = –12.4 Hz, 3′′-OC*H*$_2$Ph), 4.601 (d, 1 H, 2′′-OC*H*$_2$Ph), 4.53 (dd, 1 H, $J_{H-5,H-6b}$ = 5.0 Hz, H-6b), 4.52 (d, 1 H, *J* = –11.7 Hz, 6′-OC*H*$_2$Ph), 4.46 (d, 1 H, 3′′-OC*H*$_2$Ph), 4.39 (d, 1 H, $J_{H-1'',H-2''}$ = 0.9 Hz, H-1′′), 4.34 (dd, 1 H, $J_{H-3,H-4}$ = 8.5 Hz, $J_{H-4,H-5}$ = 9.7 Hz, H-4), 4.29 (d, 1 H, 6′-OC*H*$_2$Ph), 4.27 (dd, 1 H, $J_{H-4',H-5'}$ = 9.5

Hz, H-4′), 4.05 (dd, 1 H, H-2′), 4.01 (dd, 1 H, $J_{H-2,H-3}$ = 3.2 Hz, H-3), 3.97 (ddd, 1 H, H-5), 3.88 (dd, 1 H, $J_{H-3'',H-4''}$ = 9.7 Hz, $J_{H-4'',H-5''}$ = 9.5 Hz, H-4′′), 3.82 (dd, 1 H, H-2), 3.68 (dd, 1 H, $J_{H-5'',H-6''a}$ = 4.8 Hz, $J_{H-6''a,H-6''b}$ = –10.6 Hz, H-6′′a), 3.60 (dd, 1 H, $J_{H-2'',H-3''}$ = 3.2 Hz, H-2′′), 3.46 (dd, 1 H, $J_{H-5',H-6'a}$ = 2.0 Hz, $J_{H-6'a,H-6'b}$ = –11.9 Hz, H-6′a), 3.43 (dd, 1 H, $J_{H-5',H-6'b}$ = 4.0 Hz, H-6′b), 3.36 (s, 3 H, 1-OC*H*$_3$), 3.32 (dd, 1 H, H-3′′), 3.28 (dd, 1 H, $J_{H-5'',H-6''b}$ = 9.8 Hz, H-6′′b), 3.20 (ddd, 1 H, H-5′), 2.97 (ddd, 1 H, H-5′′) ppm.

^{13}C NMR (125.8 MHz, $CDCl_3$, 25 °C): δ = 166.3 (6-O*C*OPh), 165.4 (3′-O*C*OPh), 138.8 – 126.0 (aromatic C), 102.5 (C-1′′), 101.2 (4′′,6′′-O*C*HPh), 100.6 (C-1′), 99.1 (C-1), 78.4 (C-4′′), 78.0 (C-3′′), 77.6 (C-3), 76.9 (C-2′), 76.6 (C-2′′), 75.2 (C-5′), 75.10 (C-2, C-4), 75.06 (2′-O*C*H$_2$Ph), 74.6 (2′′-O*C*H$_2$Ph), 74.3 (C-4′), 74.0 (C-3′), 73.5 (6′-O*C*H$_2$Ph), 72.8 (2-O*C*H$_2$Ph), 72.3 (3′′-O*C*H$_2$Ph), 72.0 (3-O*C*H$_2$Ph), 69.8 (C-5), 69.0 (C-6′), 68.3 (C-6′′), 67.3 (C-5′′), 63.8 (C-6), 54.9 (1-O*C*H$_3$) ppm.

$^1J_{C-1,H-1}$ = 172 Hz (α), $^1J_{C-1',H-1'}$ = 157 Hz (β), $^1J_{C-1'',H-1''}$ = 156 Hz (β).

HRMS: calculated for $C_{82}H_{82}O_{18}Na$, $[M + Na]^+$ 1377.5393 and measured 1377.5368.

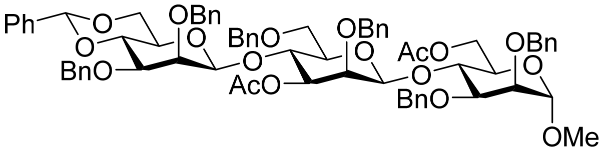

Methyl *O*-(2,3-di-*O*-benzyl-4,6-*O*-benzylidene-β-D-mannopyranosyl)-(1→4)-*O*-(3-*O*-acetyl-2,6-di-*O*-benzyl-β-D-mannopyranosyl)-(1→4)-6-*O*-acetyl-2,3-di-*O*-benzyl-α-D-mannopyranoside (11). To a solution of **10** (88 mg, 1 equivalent) in MeOH (2 ml) and THF (2 ml) was 5.4 M NaOMe in MeOH (72 µg, 6 equivalents) added. The reaction mixture was stirred for 24 h and then neutralized with DOWEX 50WX8 H^+ form. The mixture was filtered and the solvent evaporated. The product was dissolved in pyridine (3 ml) and Ac_2O (110 µl, 18 equivalents) was added. After 45 h, the reaction mixture was diluted with CH_2Cl_2 (80 ml) and washed with saturated $NaHCO_3$ solution (80 ml), 1 M HCl (80 ml) and saturated NaCl solution (50 ml). The organic phase was dried over Na_2SO_4, filtered and evaporated. The crude product was purified by column chromatography (hexane:EtOAc 2:1 and toluene:EtOAc 4:1) to provide **11** as a clear oil. Yield: 42 mg (52%), R_f = 0.20 (toluene:EtOAc 4:1).

^{1}H NMR (500.20 MHz, $CDCl_3$, 25 °C): δ = 7.49 – 7.13 (m, 35 H, aromatic H), 5.56 (s, 1 H, 4′′,6′′-OC*H*Ph), 4.89 (dd, 1 H, $J_{H-2',H-3'}$ = 3.3 Hz, $J_{H-3',H-4'}$ = 9.6, Hz, H-3′), 4.84 (d, 1 H, *J* = –11.9 Hz, 2′-OC*H*$_2$Ph), 4.75 (d, 1 H, *J* = –12.3 Hz, 3-OC*H*$_2$Ph), 4.74 (d, 1 H, $J_{H-1,H-2}$ = 2.3 Hz, H-1), 4.73 (d, 1 H, *J* = –12.1 Hz, 2′′-OC*H*$_2$Ph), 4.71 (d, 1 H, $J_{H-1',H-2'}$ = 0.8 Hz, H-1′), 4.66 (d, 1 H, *J* = –10.0 Hz, 2-OC*H*$_2$Ph), 4.648 (d, 1 H, 2′-OC*H*$_2$Ph), 4.647 (d, 1 H, 2-OC*H*$_2$Ph), 4.647 (d, 1 H, *J* = –12.4 Hz, 3′′-OC*H*$_2$Ph), 4.63 (d, 1 H, 2′′-OC*H*$_2$Ph), 4.59 (d, 1 H, 3-OC*H*$_2$Ph), 4.51 (d, 1 H, 3′′-OC*H*$_2$Ph), 4.50 (d, 1 H, *J* = –11.9 Hz, 6′-OC*H*$_2$Ph), 4.37 (d, 1 H, $J_{H-1'',H-2''}$ = 0.8 Hz, H-1′′), 4.36 (dd, 1 H, $J_{H-5,H-6a}$ = 2.2 Hz, $J_{H-6a,H-6b}$ = –12.5 Hz, H-6a), 4.30 (dd, 1 H, $J_{H-5,H-6b}$ = 5.3 Hz, H-6b), 4.26 (d, 1 H, 6′-OC*H*$_2$Ph), 4.24 (dd, 1 H, $J_{H-5'',H-6''a}$ = 5.0 Hz, $J_{H-6''a,H-6''b}$ = –10.4 Hz, H-6′′a), 4.13 (dd, 1 H, J_{H-}

$_{3,H-4}$ = 8.9 Hz, $J_{H-4,H-5}$ = 9.4 Hz, H-4), 4.11 (dd, 1 H, $J_{H-4',H-5'}$ = 9.6 Hz, H-4′), 4.07 (dd, 1 H, $J_{H-3'',H-4''}$ = 9.7 Hz, $J_{H-4'',H-5''}$ = 9.4 Hz, H-4′′), 3.94 (dd, 1 H, $J_{H-2,H-3}$ = 3.2 Hz, H-3), 3.90 (dd, 1 H, H-2′), 3.83 (ddd, 1 H, H-5), 3.81 (dd, 1 H, $J_{H-5'',H-6''b}$ = 10.1 Hz, H-6′′b), 3.77 (dd, 1 H, H-2), 3.63 (dd, 1 H, $J_{H-2'',H-3''}$ = 3.1 Hz, H-2′′), 3.41 (dd, 1 H, $J_{H-5',H-6'a}$ = 2.5 Hz, $J_{H-6'a,H-6'b}$ = –10.9 Hz, H-6′a), 3.40 (dd, 1 H, $J_{H-5',H-6'b}$ = 4.0 Hz, H-6′b), 3.38 (dd, 1 H, H-3′′), 3.33 (s, 3 H, 1-OC*H₃*), 3.17 (ddd, 1 H, H-5′), 3.15 (ddd, 1 H, H-5′′), 2.07 (s, 3 H, 3′-OCOC*H₃*), 1.94 (s, 3 H, 6-OCOC*H₃*) ppm.

^{13}C NMR (125.8 MHz, $CDCl_3$, 25 °C): δ = 170.9 (6-O*C*OCH₃), 170.2 (3′-O*C*OCH₃), 140.0 – 125.4 (aromatic C), 102.5 (C-1′′), 101.5 (4′′,6′′-O*C*HPh), 100.9 (C-1′), 99.3 (C-1), 78.8 (C-4′′), 78.3 (C-3′′), 77.8 (C-3), 77.0 (C-2′), 76.8 (C-2′′), 75.5 (C-4), 75.2 (C-5′), 75.1 (2′-O*C*H₂Ph), 74.9 (C-2), 74.9 (2′′-O*C*H₂Ph), 74.9 (6′-O*C*H₂Ph), 74.3 (C-4′), 73.6 (C-3′), 72.9 (2-O*C*H₂Ph), 72.5 (3′′-O*C*H₂Ph), 71.9 (3-O*C*H₂Ph), 69.6 (C-5), 69.1 (C-6′), 68.8 (C-6′′), 67.5 (C-5′′), 63.6 (C-6), 55.0 (1-O*C*H₃), 21.2 (3′-OCO*C*H₃), 21.0 (6-OCO*C*H₃) ppm.

HRMS: calculated for $C_{72}H_{78}O_{18}Na$, $[M + Na]^+$ 1253.5080 and measured 1253.5113.

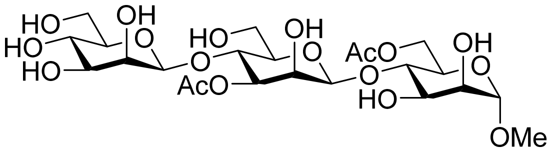

Methyl *O*-(β-D-mannopyranosyl)-(1→4)-*O*-(3-*O*-acetyl-β-D-mannopyranosyl)-(1→4)-6-*O*-acetyl-α-D-mannopyrano-side (1b). Prepared from **11** (41 mg, 0.03 mmol) according to the general method for hydrogenolysis of benzyl and benzylidene protecting groups. Yield: 18.2 mg (91%).

^{1}H NMR (500.20 MHz, MeOD, 25 °C): δ = 5.02 (dd, 1 H, $J_{H-2',H-3'}$ = 3.2 Hz, $J_{H-3',H-4'}$ = 9.9 Hz, H-3′), 4.69 (d, 1 H, $J_{H-1',H-2'}$ = 0.6 Hz, H-1′), 4.619 (d, 1 H, $J_{H-1,H-2}$ = 2.0 Hz, H-1), 4.616 (d, 1 H, $J_{H-1'',H-2''}$ = 0.6 Hz, H-1′′), 4.35 (dd, 1 H, $J_{H-5,H-6a}$ = 2.4 Hz, $J_{H-6a,H-6b}$ = –11.9 Hz, H-6a), 4.28 (dd, 1 H, $J_{H-5,H-6b}$ = 5.5 Hz, H-6b), 4.09 (dd, 1 H, $J_{H-4',H-5'}$ = 9.8 Hz, H-4′), 4.06 (dd, 1 H, H-2′), 3.92 (dd, 1 H, $J_{H-5',H-6'a}$ = 1.9 Hz, $J_{H-6'a,H-6'b}$ = –12.2 Hz, H-6′a), 3.87 (dd, 1 H, $J_{H-2,H-3}$ = 3.3 Hz, H-2), 3.869 (dd, 1 H, $J_{H-3,H-4}$ = 9.0 Hz, $J_{H-4,H-5}$ = 9.9 Hz, H-4), 3.866 (dd, 1 H, $J_{H-5'',H-6''a}$ = 2.6 Hz, $J_{H-6''a,H-6''b}$ = –11.7 Hz, H-6′′a), 3.83 (dd, 1 H, $J_{H-2'',H-3''}$ = 3.2 Hz, H-2′′), 3.81 (dd, 1 H, H-3), 3.76 (ddd, 1 H, H-5), 3.72 (dd, 1 H, $J_{H-5',H-6'a}$ = 4.8 Hz, H-6′b), 3.71 (dd, 1 H, $J_{H-5''b,H-6''}$ = 6.0 Hz, H-6′′b), 3.53 (ddd, 1 H, H-5′), 3.52 (dd, 1 H, $J_{H-3'',H-4''}$ = 9.5 Hz, $J_{H-4'',H-5''}$ = 9.7 Hz, H-4′′), 3.44 (dd, 1 H, H-3′′), 3.36 (s, 3 H, 1-OC*H₃*), 3.22 (ddd, 1 H, H-5′′), 2.14 (s, 3 H, 3′-OCOC*H₃*), 2.08 (s, 3 H, 6-OCOC*H₃*) ppm.

^{13}C NMR (125.8 MHz, MeOD, 25 °C): δ = 171.3 (6-O*C*OCH₃), 171.1 (3′-O*C*OCH₃), 101.2 (C-1), 99.9 (C-1′), 99.9 (C-1′′), 77.2 (C-4), 77.0 (C-5′′), 76.1 (C-5′), 73.7 (C-3′′), 73.1 (C-3′), 72.3 (C-4′), 71.1 (C-2′′), 70.0 (C-2), 69.6 (C-3), 69.0 (C-2′), 68.9 (C-5), 67.0 (C-4′′), 63.0 (C-6), 61.6 (C-6′′), 60.7 (C-6′), 55.9 (1-O*C*H₃), 19.8 (3′-OCO*C*H₃), 19.4 (6-OCO*C*H₃) ppm.

HRMS: calculated for $C_{23}H_{38}O_{18}Na$, $[M + Na]^+$ 625.1950 and measured 625.2022.

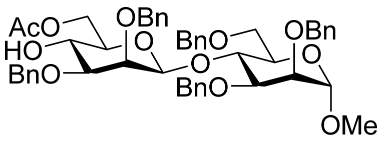

Methyl *O*-(6-*O*-acetyl-2,3-di-*O*-benzyl-β-D-mannopyranosyl)-(1→4)-2,3,6-tri-*O*-benzyl-α-D-mannopyranoside (13). To a solution of **12** (160 mg, 1 equivalent) in pyridine (3 ml) at 0 °C was AcCl (14 µl, 1 equivalents) added. After 76 h, the reaction mixture was diluted with CH_2Cl_2 (80 ml) and washed with saturated $NaHCO_3$ solution (80 ml), 1 M HCl (80 ml) and saturated NaCl solution (50 ml). The organic phase was dried over Na_2SO_4, filtered and evaporated. The crude product was purified by column chromatography (hexane:EtOAc 1:2) to provide **13** as a clear oil. Yield: 140 mg (85%), R_f = 0.40.

^{1}H NMR (500.20 MHz, $CDCl_3$, 25 °C): δ = 7.29 – 7.13 (m, 25 H, aromatic H), 4.77 (d, 1 H, *J* = –12.1 Hz, 3-OC*H*$_2$Ph), 4.71 (d, 1 H, *J* = –12.0 Hz, 2′-OC*H*$_2$Ph), 4.68 (d, 1 H, $J_{H-1,H-2}$ = 2.3 Hz, H-1), 4.64 (d, 1 H, *J* = –12.0 Hz, 2-OC*H*$_2$Ph), 4.62 (d, 1 H, *J* = –12.0 Hz, 6-OC*H*$_2$Ph), 4.602 (d, 1 H, 2′-OC*H*$_2$Ph), 4.596 (d, 1 H, 2-OC*H*$_2$Ph), 4.51 (d, 1 H, 3-OC*H*$_2$Ph), 4.48 (d, 1 H, $J_{H-1',H-2'}$ = 0.8 Hz, H-1′), 4.39 (d, 1 H, 6-OC*H*$_2$Ph), 4.38 (d, 1 H, *J* = –11.8 Hz, 3′-OC*H*$_2$Ph), 4.24 (d, 1 H, 3′-OC*H*$_2$Ph), 4.16 (dd, 1 H, $J_{H-3,H-4}$ = 8.5 Hz, $J_{H-4,H-5}$ = 9.4 Hz, H-4), 4.15 (dd, 1 H, $J_{H-5'a,H-6'a}$ = 5.4 Hz, $J_{H-6'a,H-6'b}$ = –12.0 Hz, H-6′a), 4.12 (dd, 1 H, $J_{H-5',H-6'b}$ = 2.2 Hz, H-6′b),3.86 (dd, 1 H, $J_{H-2,H-3}$ = 3.1 Hz, H-3), 3.74 (dd, 1 H, $J_{H-3',H-4'}$ = 9.4 Hz, $J_{H-4',H-5'}$ = 9.8 Hz, $J_{H-4',4'-OH}$ = 2.6 Hz, H-4′), 3.70 (ddd, 1 H, $J_{H-5,H-6a}$ = 4.9 Hz, $J_{H-5,H-6b}$ = 4.9 Hz, H-5), 3.682 (dd, 1 H, $J_{H-2',H-3'}$ = 2.9 Hz, H-2′), 3.679 (dd, 1 H, H-2), 3.678 (dd, 1 H, $J_{H-6a,H-6b}$ = –10.4 Hz, H-6a), 3.61 (dd, 1 H, H-6b), 3.28 (s, 3 H, 1-OC*H*$_3$), 3.05 (dd, 1 H, H-3′), 3.11 (ddd, 1 H, H-5′), 2.35 (d, 1 H, 4-O*H*), 1.84 (s, 3 H, 6-OCOC*H*$_3$) ppm.

^{13}C NMR (125.78 MHz, $CDCl_3$, 25 °C): δ = 171.6 (6′-O*C*OCH$_3$), 139.3 – 127.5 (aromatic C), 101.7 (C-1′), 99.5 (C-1), 81.7 (C-3′), 78.2 (C-3), 75.8(C-4), 75.7 (C-2), 74.4 (C-2′), 74.3 (C-5′),74.2 (2′-O*C*H$_2$Ph), 73.6 (6-O*C*H$_2$Ph), 72.9 (2-O*C*H$_2$Ph), 72.6 (3-O*C*H$_2$Ph), 71.5 (3′-O*C*H$_2$Ph), 71.3 (C-5), 69.6 (C-6), 66.6 (C-4′), 63.8 (C-6′), 55.1 (1-O*C*H$_3$), 20.9 (6′-OCO*C*H$_3$) ppm.

HRMS: calculated for $C_{50}H_{56}O_{12}Na$, $[M + Na]^+$ 871.3664, measured 871.3660.

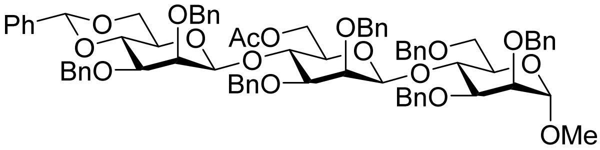

Methyl *O*-(2,3-di-*O*-benzyl-4,6-*O*-benzylidene-β-D-mannopyranosyl)-(1→4)-*O*-(6-*O*-acetyl-2,3-di-*O*-benzyl-β-D-mannopyranosyl)-(1→4)-2,3,6-tri-*O*-benzyl-α-D-mannopyranoside (14). Prepared from donor **9** (66 mg, 0.12 mmol) and acceptor **13** (84 mg, 0.10 mmol) according to the standard reaction procedure for β-mannosylation. The crude product was purified by column chromatography (toulene:EtOAc 7:1) to provide **14** as a white foam. Yield: 33 mg (26%), R_f = 0.24.

^{1}H NMR (500.20 MHz, $CDCl_3$, 25 °C): δ = 7.39 – 7.06 (m, 40 H, aromatic H), 5.43 (s, 1 H, 4″,6″-OC*H*Ph), 4.79 (d, 1 H, *J* = –12.0 Hz, 3-OC*H*$_2$Ph), 4.78 (d, 1 H, *J* = –11.8 Hz, 2″-OC*H*$_2$Ph), 4.72 (d, 1 H, 2″-OC*H*$_2$Ph), 4.67 (d, 1 H, $J_{H-1,H-2}$ = 2.2 Hz, H-1), 4.64 (d, 1 H, *J* = –12.2 Hz, 2-OC*H*$_2$Ph), 4.654 (d, 1 H, *J* = –12.4 Hz, 3″-

OCH_2Ph), 4.647 (d, 1 H, J = –12.2 Hz, 2′-OCH_2Ph), 4.63 (d, 1 H, 2′-OCH_2Ph), 4.59 (d, 1 H, 2-OCH_2Ph), 4.585 (d, 1 H, J = –12.1 Hz, 6-OCH_2Ph), 4.578 (d, 1 H, J = –11.9 Hz, 3′-OCH_2Ph), 4.54 (d, 1 H, 3″-OCH_2Ph), 4.51 (d, 1 H, $J_{\text{H-1′,H-2′}}$ = 0.8 Hz, H-1′), 4.50 (d, 1 H, 3-OCH_2Ph), 4.53 (d, 1 H, $J_{\text{H-1″,H-2″}}$ = 0.8 Hz, H-1″), 4.3637 (d, 1 H, 3′-OCH_2Ph), 4.3636 (d, 1 H, 6-OCH_2Ph), 4.13 (dd, 1 H, $J_{\text{H-3,H-4}}$ = 8.9 Hz, $J_{\text{H-4,H-5}}$ = 9.0 Hz, H-4), 4.023 (dd, 1 H, $J_{\text{H-5′,H-6′a}}$ = 3.4 Hz, $J_{\text{H-6′a,H-6′b}}$ = –11.9 Hz, H-6′a), 4.021 (dd, 1 H, $J_{\text{H-5′,H-6′b}}$ = 3.8 Hz, H-6′b), 4.00 (dd, 1 H, $J_{\text{H-3″,H-4″}}$ = 9.8 Hz, $J_{\text{H-4″,H-5″}}$ = 9.5 Hz, H-4″), 3.88 (dd, 1 H, $J_{\text{H-3′,H-4′}}$ = 9.3 Hz, $J_{\text{H-4′,H-5′}}$ = 9.3 Hz, H-4′), 3.85 (dd, 1 H, $J_{\text{H-5″,H-6″a}}$ = 4.7 Hz, $J_{\text{H-6″a,H-6″b}}$ = –10.5 Hz, H-6″a), 3.82 (dd, 1 H, $J_{\text{H-2,H-3}}$ = 3.1 Hz, H-3), 3.80 (dd, 1 H, $J_{\text{H-2″,H-3″}}$ = 2.8 Hz, H-2″), 3.66 (dd, 1 H, H-2), 3.65 (ddd, 1 H, $J_{\text{H-5,H-6a}}$ = 4.2 Hz, $J_{\text{H-5,H-6b}}$ = 1.9 Hz, H-5), 3.62 (dd, 1 H, $J_{\text{H-6a,H-6b}}$ = –10.3 Hz, H-6a), 3.63 (dd, 1 H, $J_{\text{H-2′,H-3′}}$ = 3.0 Hz, H-2′), 3.58 (dd, 1 H, H-6b), 3.51 (dd, 1 H, $J_{\text{H-5″,H-6″b}}$ = 10.0 Hz, H-6″b), 3.44 (dd, 1 H, H-3″), 3.2673 (dd, 1 H, H-3′), 3.2671 (s, 3 H, 1-OCH_3), 3.20 (ddd, 1 H, H-5′), 2.99 (ddd, 1 H, H-5″), 1.79 (s, 3 H, 6′-OCOCH_3) ppm.

^{13}C NMR (125.8 MHz, $CDCl_3$, 25 °C): δ = 169.8 (6′- O*C*OCH_3), 138.2 – 124.3 (aromatic C), 101.0 (C-1″), 100.4 (C-1′), 100.3 (4″,6″-O*C*HPh), 98.3 (C-1), 79.3 (C-3′), 77.5 (C-4″), 77.34 (C-3), 77.28 (C-3″), 76.3 (C-2″), 74.79 (C-4), 74.77 (C-4′),74.55 (C-2′), 74.53 (C-2), 74.2 (2″-OCH_2Ph), 73.1 (2′-OCH_2Ph), 72.4 (6-OCH_2Ph), 72.2 (C-5′), 71.7 (2-OCH_2Ph), 71.48 (3″-OCH_2Ph), 71.46 (3-OCH_2Ph), 70.9 (3′-OCH_2Ph), 70.1 (C-5), 68.4 (C-6), 67.4 (C-6″), 66.4 (C-5″), 62.2 (C-6′), 53.9 (1-OCH_3), 19.7 (6′- OCOCH_3) ppm.

$^{1}J_{\text{C-1,H-1}}$ = 165 Hz (α), $^{1}J_{\text{C-1′,H-1′}}$ = 157 Hz (β), $^{1}J_{\text{C-1″,H-1″}}$ = 156 Hz (β).

HRMS: calculated for $C_{77}H_{82}O_{17}Na$, $[M + Na]^+$ 1301.5444, measured 1301.5429.

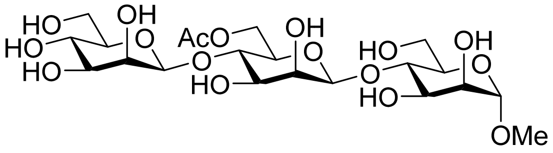

Methyl *O*-(β-D-mannopyranosyl)-(1→4)-*O*-(6-*O*-acetyl-β-D-mannopyranosyl)-(1→4)-α-D-mannopyranoside (2): Prepared from **14** (41 mg, 0.03 mmol) according to the general method for hydrogenolysis of benzyl and benzylidene protecting groups. Yield: 18.2 mg (91%).

^{1}H NMR (500.20 MHz, MeOD, 25 °C): δ = 4.66 (d, 1 H, $J_{\text{H-1′,H-2′}}$ = 0.8 Hz, H-1′), 4.65 (d, 1 H, $J_{\text{H-1,H-2}}$ = 1.7 Hz, H-1), 4.58 (d, 1 H, $J_{\text{H-1″,H-2″}}$ = 0.8 Hz, H-1″), 4.46 (dd, 1 H, $J_{\text{H-5′,H-6′a}}$ = 2.3 Hz, $J_{\text{H-6′a,H-6′b}}$ = –11.9 Hz, H-6′a), 4.30 (dd, 1 H, $J_{\text{H-5′,H-6′a}}$ = 5.7 Hz, H-6′b), 4.01 (dd, 1 H, $J_{\text{H-2′,H-3′}}$ = 3.1 Hz, H-2′), 3.92 (dd, 1 H, $J_{\text{H-2″,H-3″}}$ = 3.1 Hz, H-2″), 3.91 (dd, 1 H, $J_{\text{H-5″,H-6″a}}$ = 2.3 Hz, $J_{\text{H-6″a,H-6″b}}$ = –11.9 Hz, H-6″a), 3.89 (dd, 1 H, $J_{\text{H-3,H-4}}$ = 9.5 Hz, $J_{\text{H-4,H-5}}$ = 9.5 Hz, H-4), 3.87 (dd, 1 H, $J_{\text{H-2,H-3}}$ = 3.4 Hz, H-2), 3.84 (dd, 1 H, $J_{\text{H-3′,H-4′}}$ = 9.2 Hz, $J_{\text{H-4′,H-5′}}$ = 9.5 Hz, H-4′), 3.81 (dd, 1 H, H-3), 3.78 (dd, 1 H, $J_{\text{H-5,H-6a}}$ = 2.1 Hz, $J_{\text{H-6a,H-6b}}$ = –12.1 Hz, H-6a), 3.73 (dd, 1 H, $J_{\text{H-5,H-6b}}$ = 4.3 Hz, H-6b), 3.69 (dd, 1 H, $J_{\text{H-5″b,H-6″}}$ = 6.6 Hz, H-6″b), 3.68 (dd, 1 H, H-3′), 3.63 (ddd, 1 H, H-5′), 3.58 (ddd, 1 H, H-5), 3.52 (dd, 1 H, $J_{\text{H-3″,H-4″}}$ = 9.2 Hz, $J_{\text{H-4″,H-5″}}$ = 9.9 Hz, H-4″), 3.46 (dd, 1 H, H-3″), 3.36 (s, 3 H, 1-OCH_3), 3.29 (ddd, 1 H, H-5″), 2.10 (s, 3 H, 6′-OCOCH_3) ppm.

^{13}C NMR (125.8 MHz, MeOD, 25 °C): δ = 172.9 (6′-OCOCH$_3$), 102.6 (C-1), 102.2 (C-1′), 101.9 (C-1′′), 78.9 (C-5′′), 78.6 (C-4), 78.0 (C-4′), 75.1 (C-3′′), 74.4 (C-5′), 73.4 (C-3′), 72.7 (C-5), 72.4 (C-2′′), 71.7 (C-2′), 71.5 (C-2), 71.1 (C-3), 68.4 (C-4′′), 64.0 (C-6′), 62.8 (C-6′′), 62.1 (C-6), 55.4 (1-OCH$_3$), 20.9 (6′-OCOCH$_3$) ppm.

HRMS: calculated for $C_{21}H_{36}O_{17}Na$, $[M + Na]^+$ 583.1845 and measured 583.1835.

References

(1) ChemAdder/SpinAdder. Spin Discoveries Ltd (http://www.chemadder.com).

(2) Lassfolk, R.; Rahkila, J.; Johansson, M. P.; Ekholm, F. S.; Wärnå, J.; Leino, R. Acetyl Group Migration across the Saccharide Units in Oligomannoside Model Compound. *J. Am. Chem. Soc.* **2019**, *141*, 1646–1654.

(3) Ono, F.; Watanabe, H.; Shinkai, S. Structural Optimization of Super-Gelators Derived from Naturally-Occurring Mannose and Their Morphological Diversity. *RSC Adv.* **2014**, *4*, 25940–25947.

(4) Rao, K. V.; Patil, P. R.; Atmakuri, S.; Kartha, K. P. R. Iodine-Sodium Cyanoborohydride-Mediated Reductive Ring Opening of 4,6-O-Benzylidene Acetals of Hexopyranosides. *Carbohydr. Res.* **2010**, *345*, 2709–2713.

(5) Crich, D.; Smith, M. 1-Benzenesulfinyl Piperidine/Trifluoromethanesulfonic Anhydride: A Potent Combination of Shelf-Stable Reagents for the Low-Temperature Conversion of Thioglycosides to Glycosyl Triflates and for the Formation of Diverse Glycosidic Linkages. *J. Am. Chem. Soc.* **2001**, *123*, 9015–9020.

(6) Crich, D.; Chandrasekera, N. S. Mechanism of 4,6-O-Benzylidene-Directed β-Mannosylation as Determined by α-Deuterium Kinetic Isotope Effects. *Angew. Chemie - Int. Ed.* **2004**, *43*, 5386–5389.

(7) Zemplén, G.; Kunz, A. Studien Über Amygdalin, IV: Synthese Des Natürlichen l-Amygdalins. *Ber. Dtsch. Chem. Ges.* **1924**, *57*, 1357–1359.

(8) Kim, K. S.; Fulse, D. B.; Baek, J. Y.; Lee, B.-Y.; Jeon, H. B. Stereoselective Direct Glycosylation with Anomeric Hydroxy Sugars by Activation with Phthalic Anhydride and Trifluoromethanesulfonic Anhydride Involving Glycosyl Phthalate Intermediates. *J. Am. Chem. Soc.* **2008**, *130*, 8537–8547.

NMR spectra

Methyl 2,3-di-*O*-benzyl-6-*O*-*p*-methoxybenzyl-α-D-mannopyranoside (4)

^{1}H NMR (500.20 MHz, $CDCl_3$, 25°C):

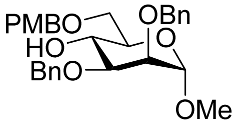

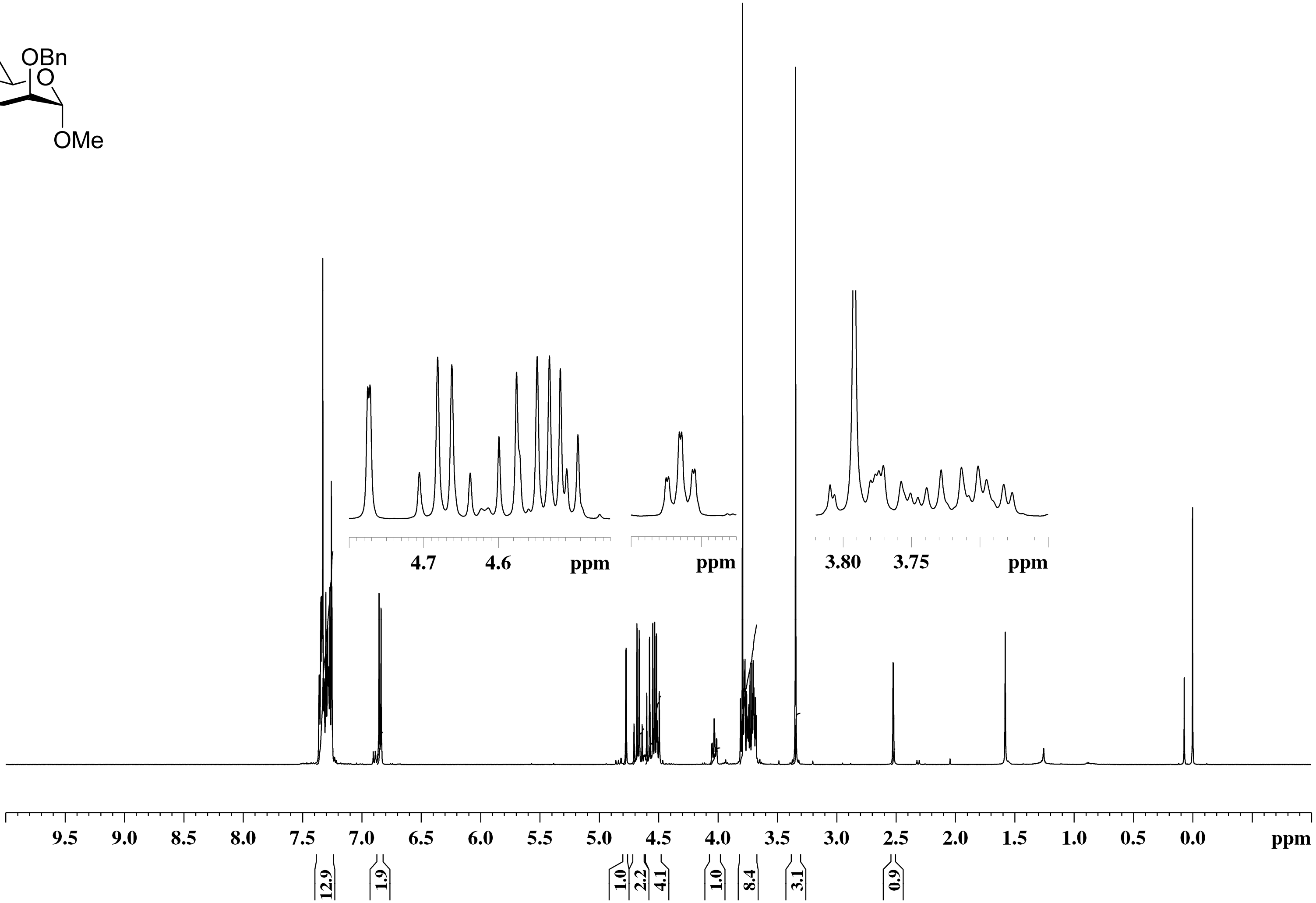

^{13}C NMR (125.8 MHz, $CDCl_3$, 25°C):

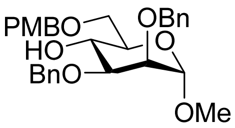

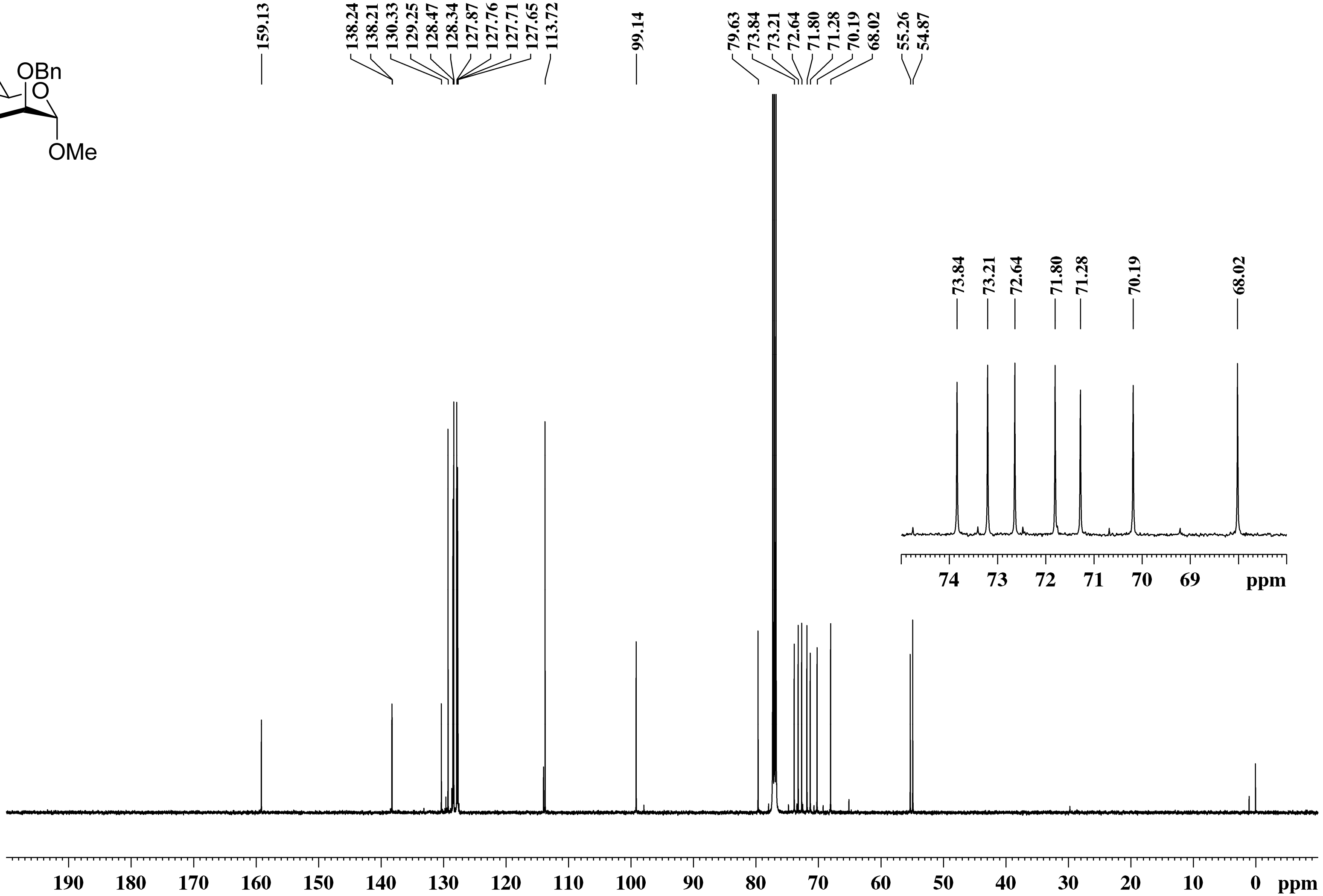

Methyl *O*-(4,6-*O*-benzylidene-2-*O*-benzyl-3-*O*-*p*-methoxybenzyl-β-D-mannopyranosyl)-(1→4)-2,3-di-*O*-benzyl-6-*O*-*p*-methoxybenzyl-α-D-mannopyranoside (6)

^{1}H NMR (500.20 MHz, $CDCl_3$, 25°C):

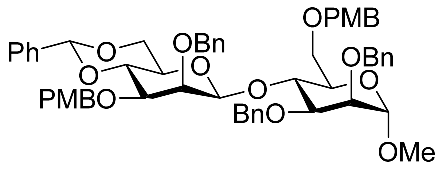

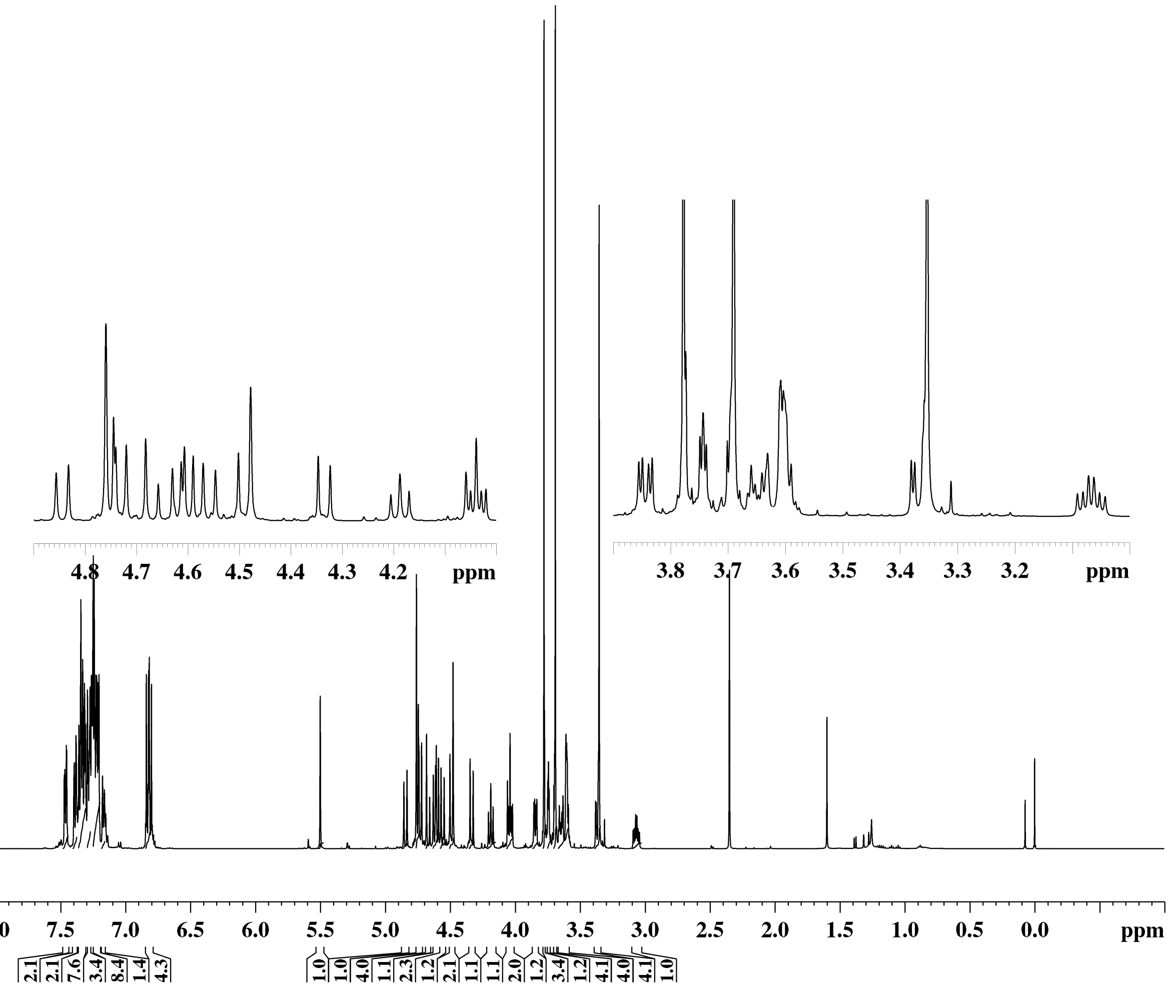

^{13}C NMR (125.8 MHz, $CDCl_3$, 25°C):

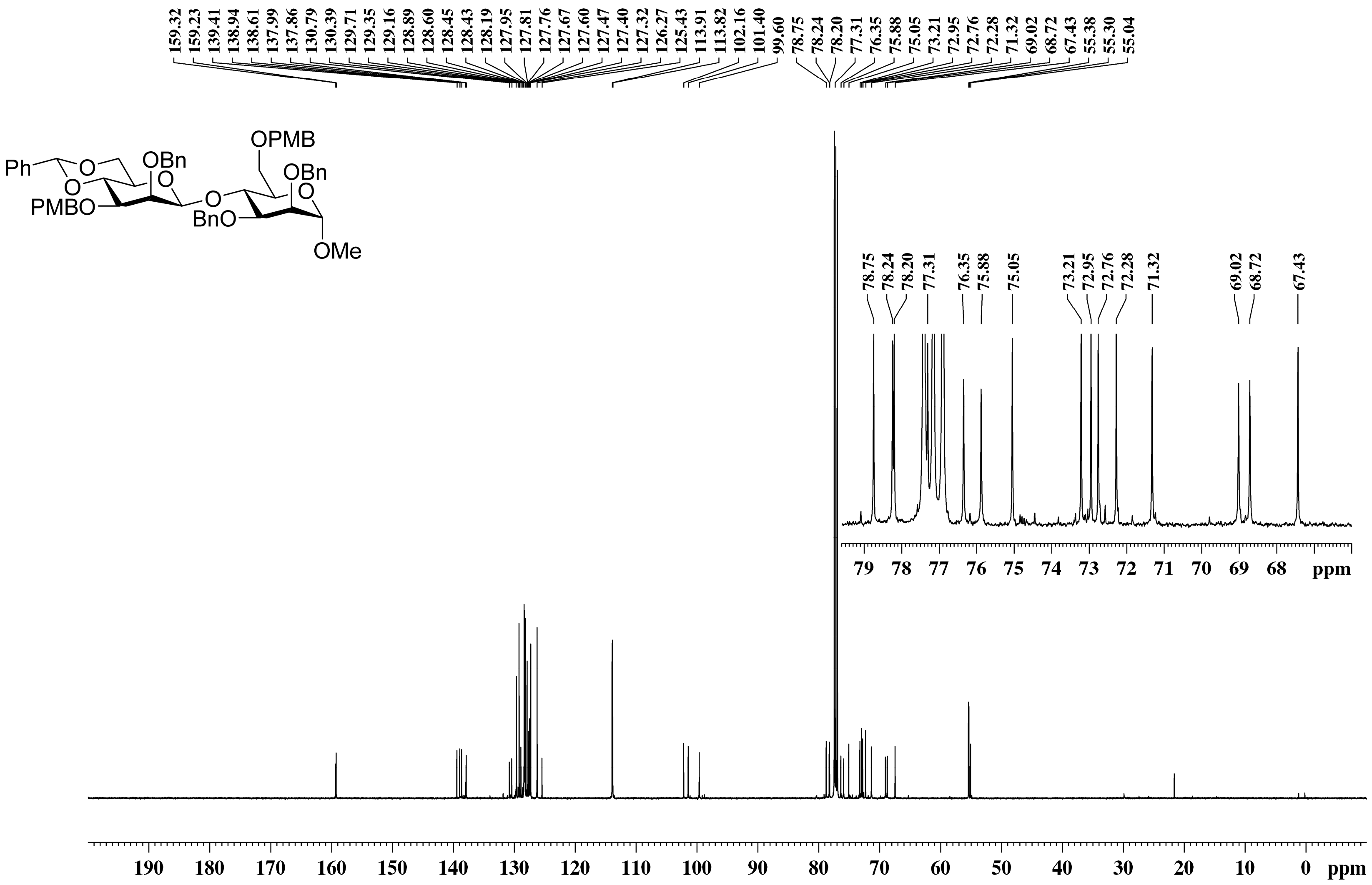

Methyl *O*-(3-*O*-benzoyl-2-O-benzyl-4,6-*O*-benzylidene-β-D-mannopyranosyl)-(1→4)-2,3-di-*O*-benzyl-6-*O*-benzoyl-α-D-mannopyranoside (7)

^{1}H NMR (500.20 MHz, CDCl$_3$, 25°C):

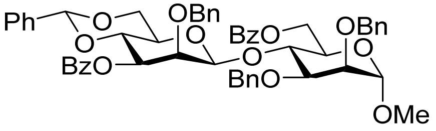

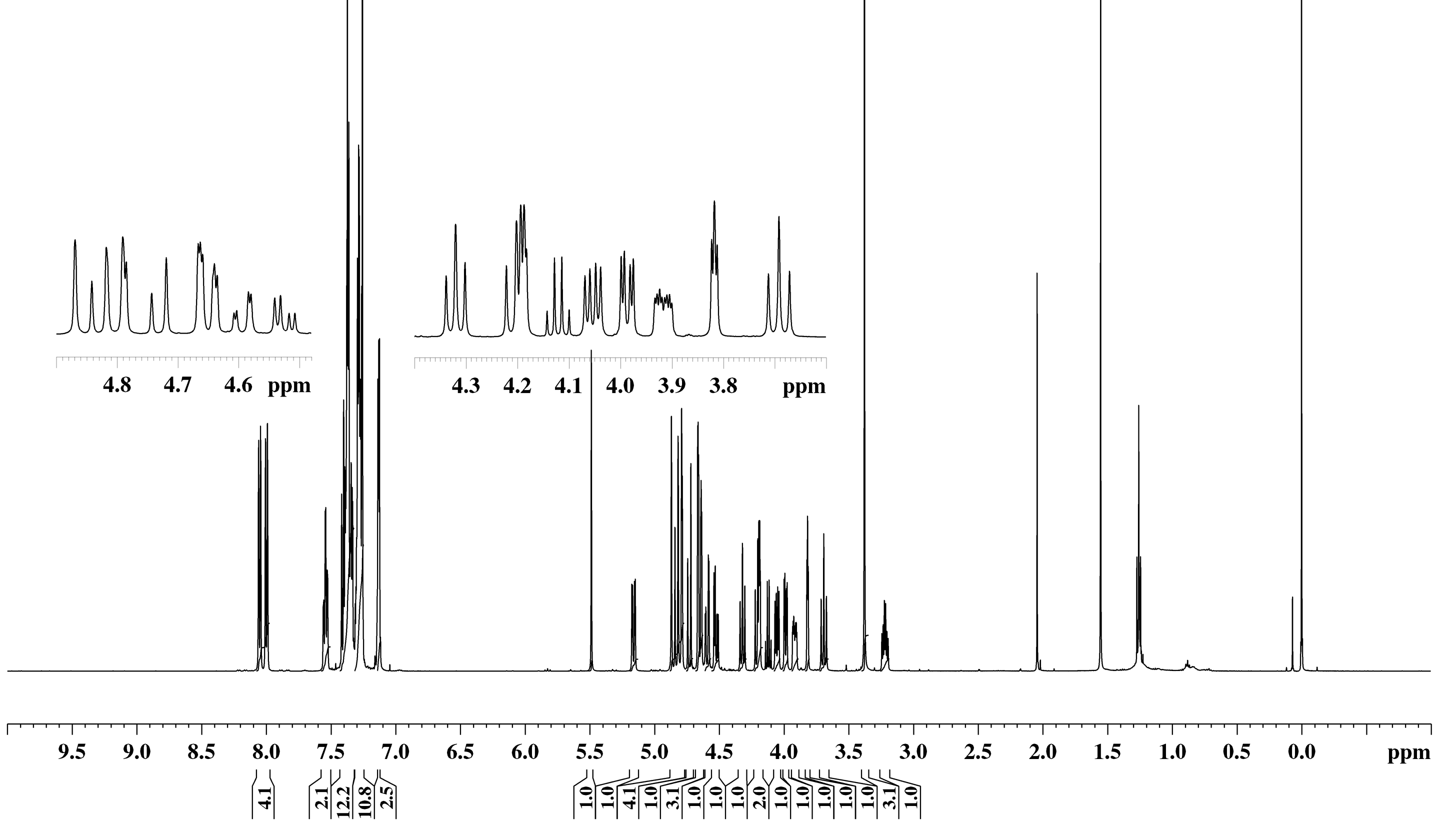

^{13}C NMR (125.8 MHz, $CDCl_3$, 25°C):

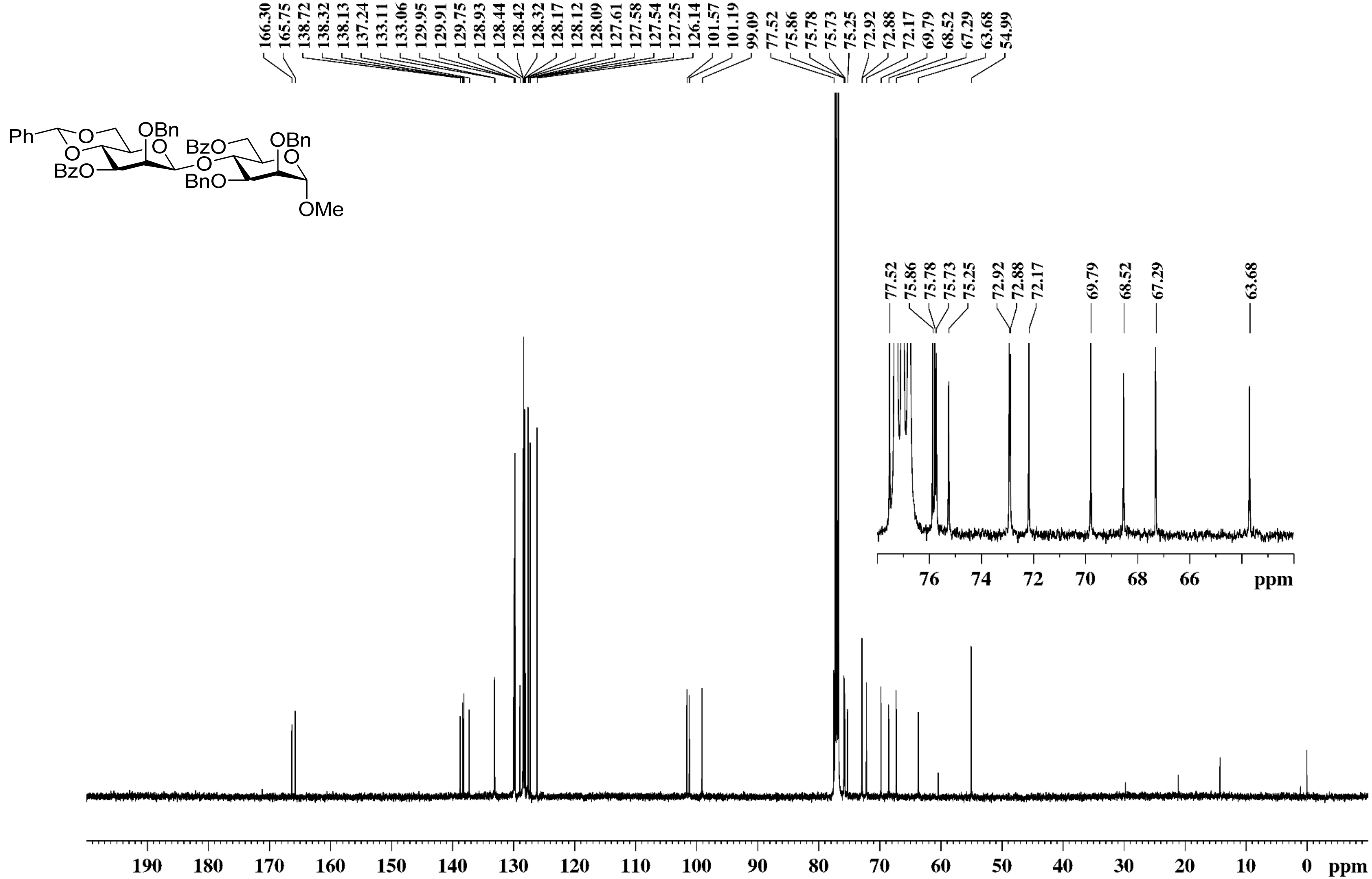

Methyl *O*-(3-*O*-benzoyl-2,6-di-*O*-benzyl-β-D-mannopyranosyl)-(1→4)-6-*O*-benzoyl-2,3-di-*O*-benzyl-α-D-mannopyranoside (8)

^{1}H NMR (500.20 MHz, $CDCl_3$, 25°C):

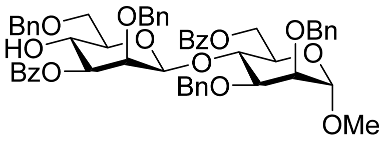

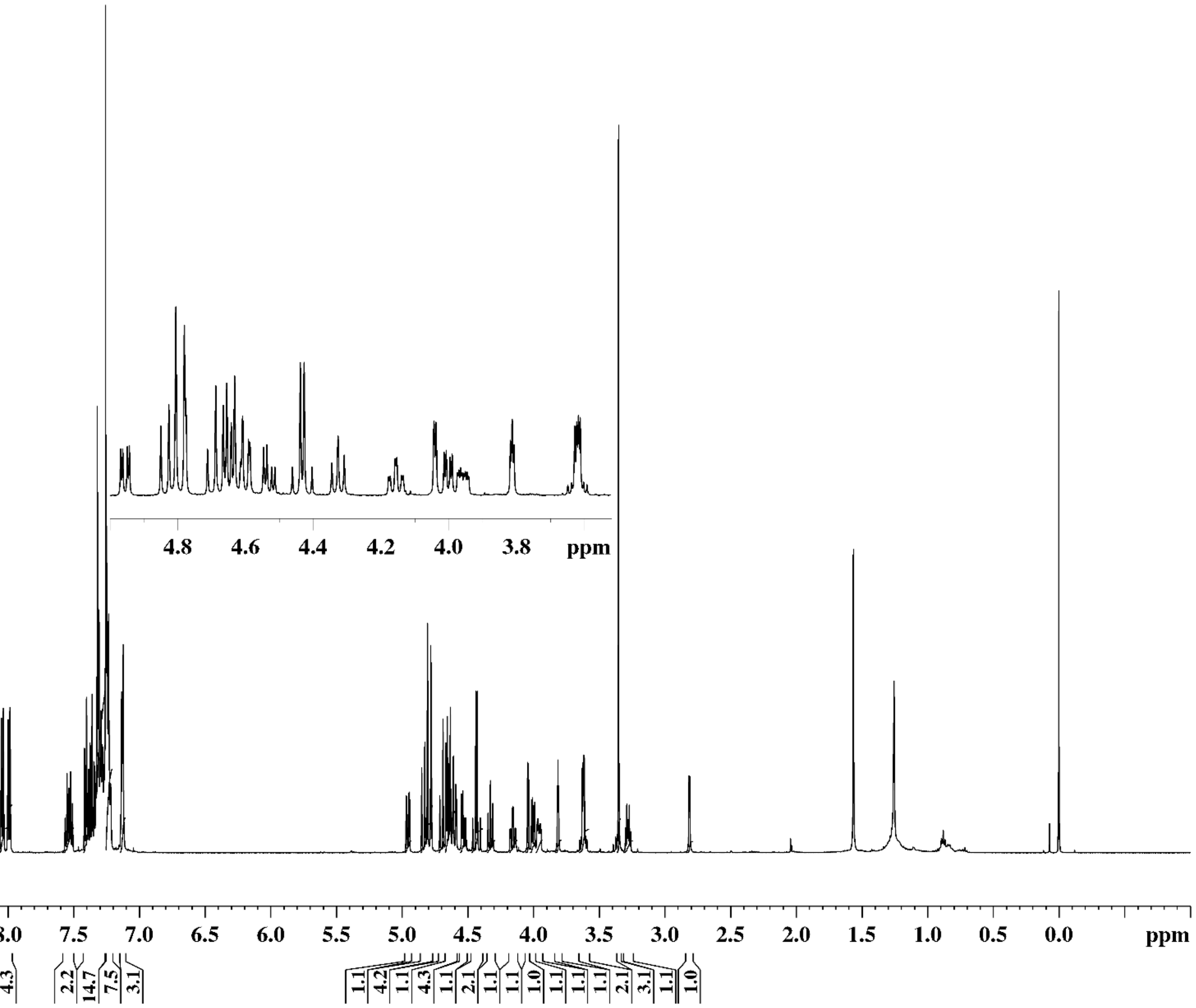

^{13}C NMR (125.8 MHz, $CDCl_3$, 25°C):

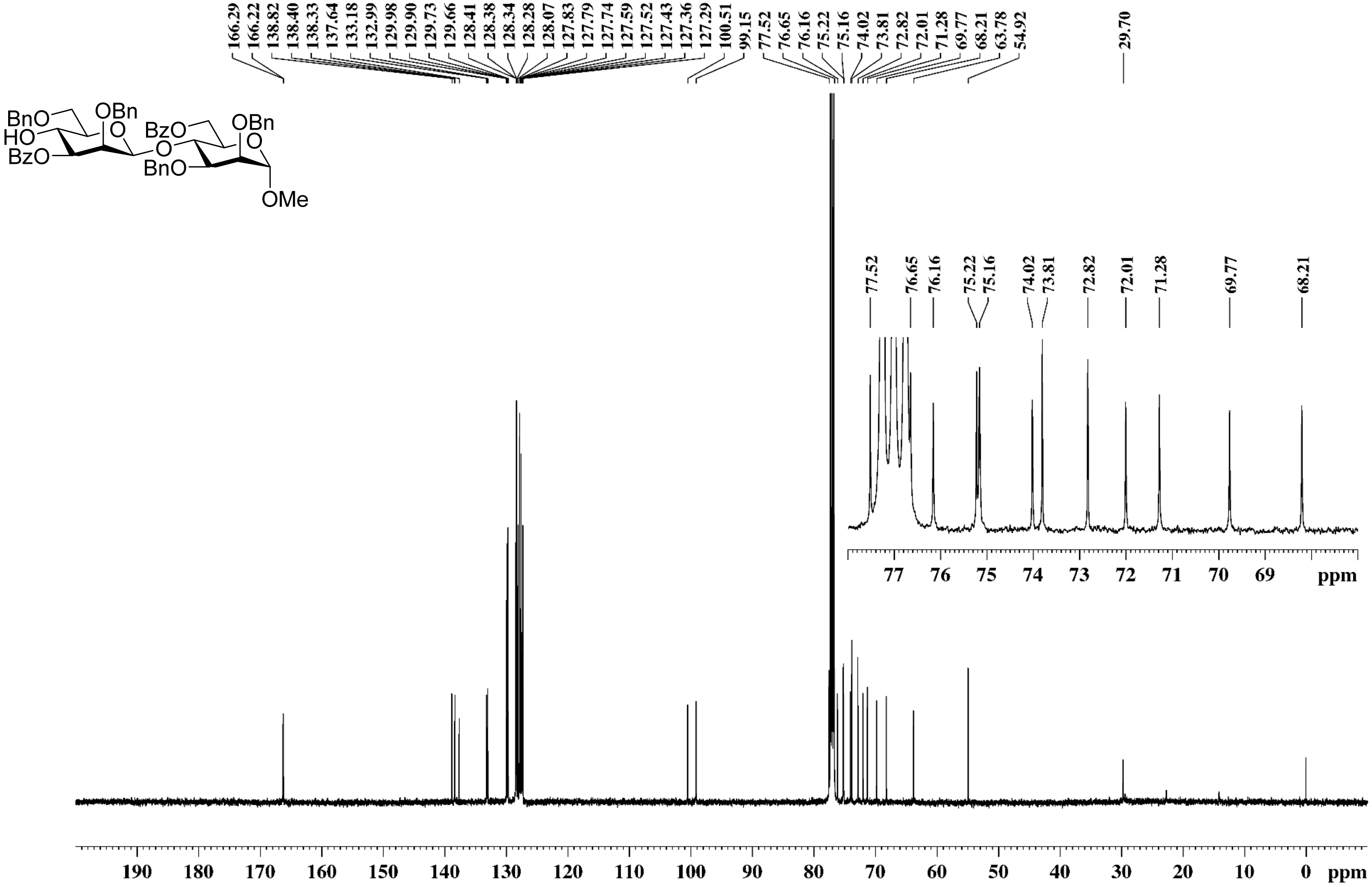

Methyl *O*-(2,3-di-*O*-benzyl-4,6-*O*-benzylidene-β-D-mannopyranosyl)-(1→4)-*O*-(3-*O*-benzoyl-2,6-di-*O*-benzyl-β-D-mannopyranosyl)-(1→4)-6-*O*-benzoyl-2,3-di-*O*-benzyl-α-D-mannopyranoside (10)

^{1}H NMR (500.20 MHz, $CDCl_3$, 25°C):

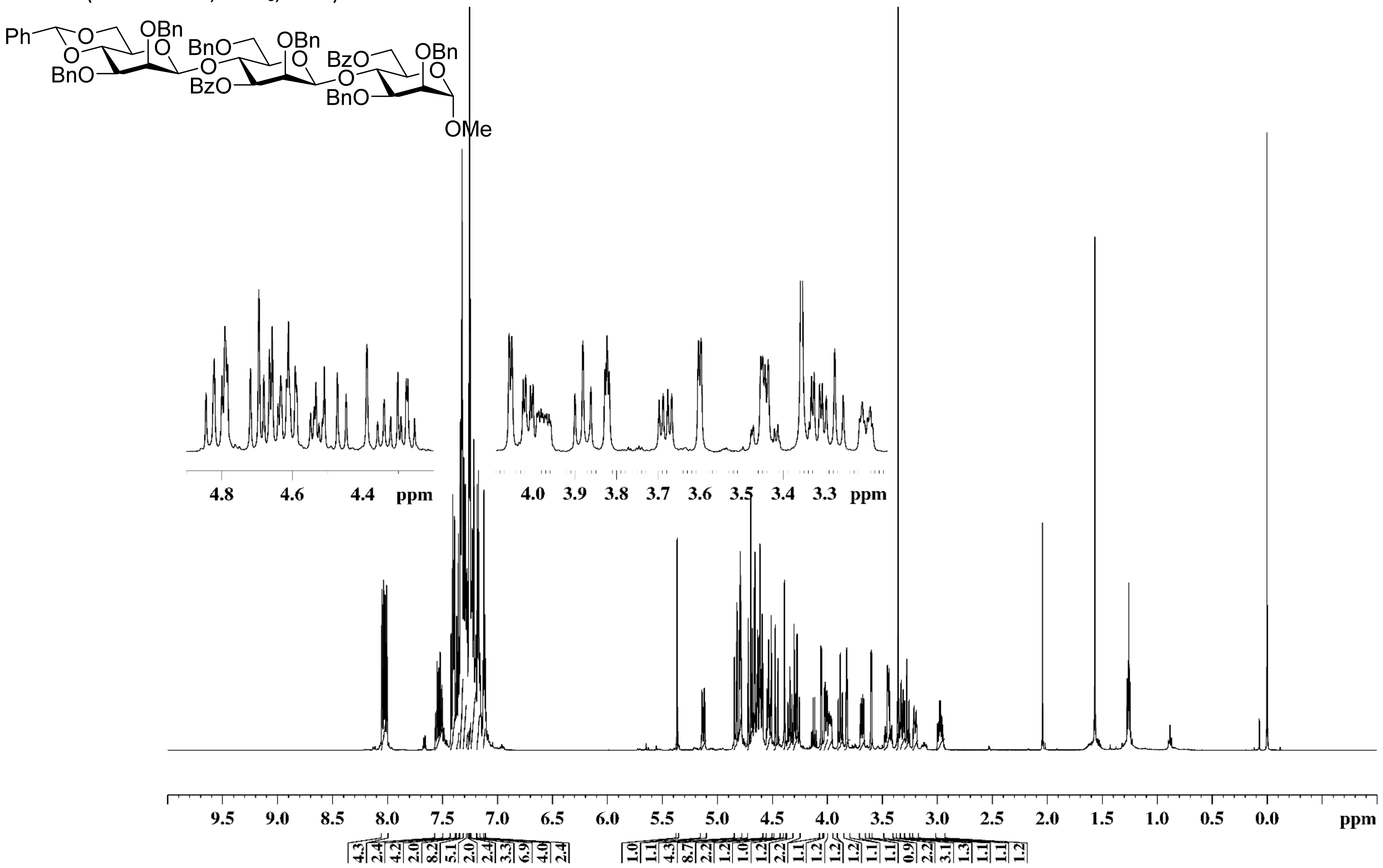

^{13}C NMR (125.8 MHz, $CDCl_3$, 25°C):

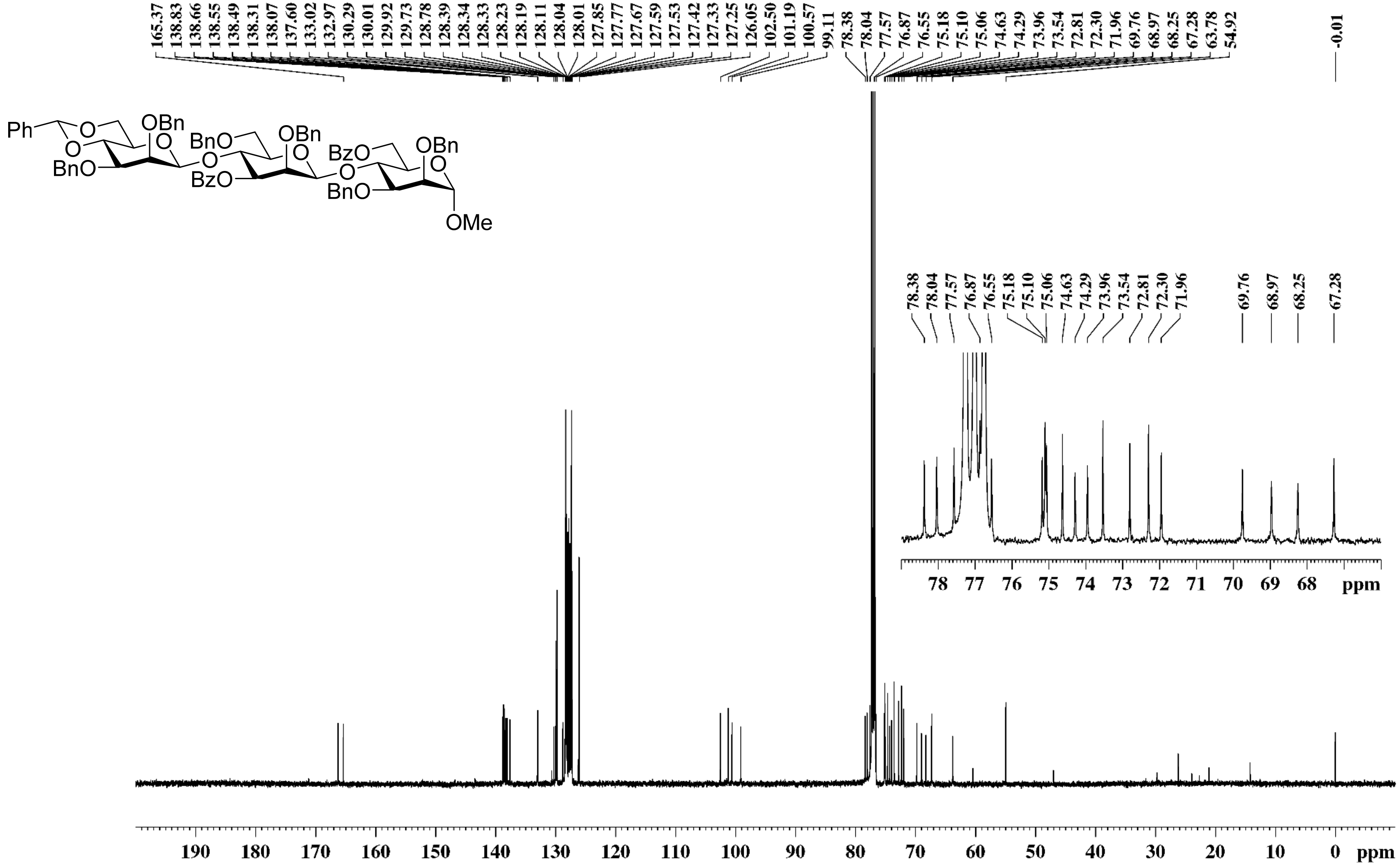

Methyl *O*-(2,3-di-*O*-benzyl-4,6-*O*-benzylidene-β-D-mannopyranosyl)-(1→4)-*O*-(3-*O*-acetyl-2,6-di-*O*-benzyl-β-D-mannopyranosyl)-(1→4)-6-*O*-acetyl-2,3-di-*O*-benzyl-α-D-mannopyranoside (11)

^{1}H NMR (500.20 MHz, CDCl$_3$, 25°C):

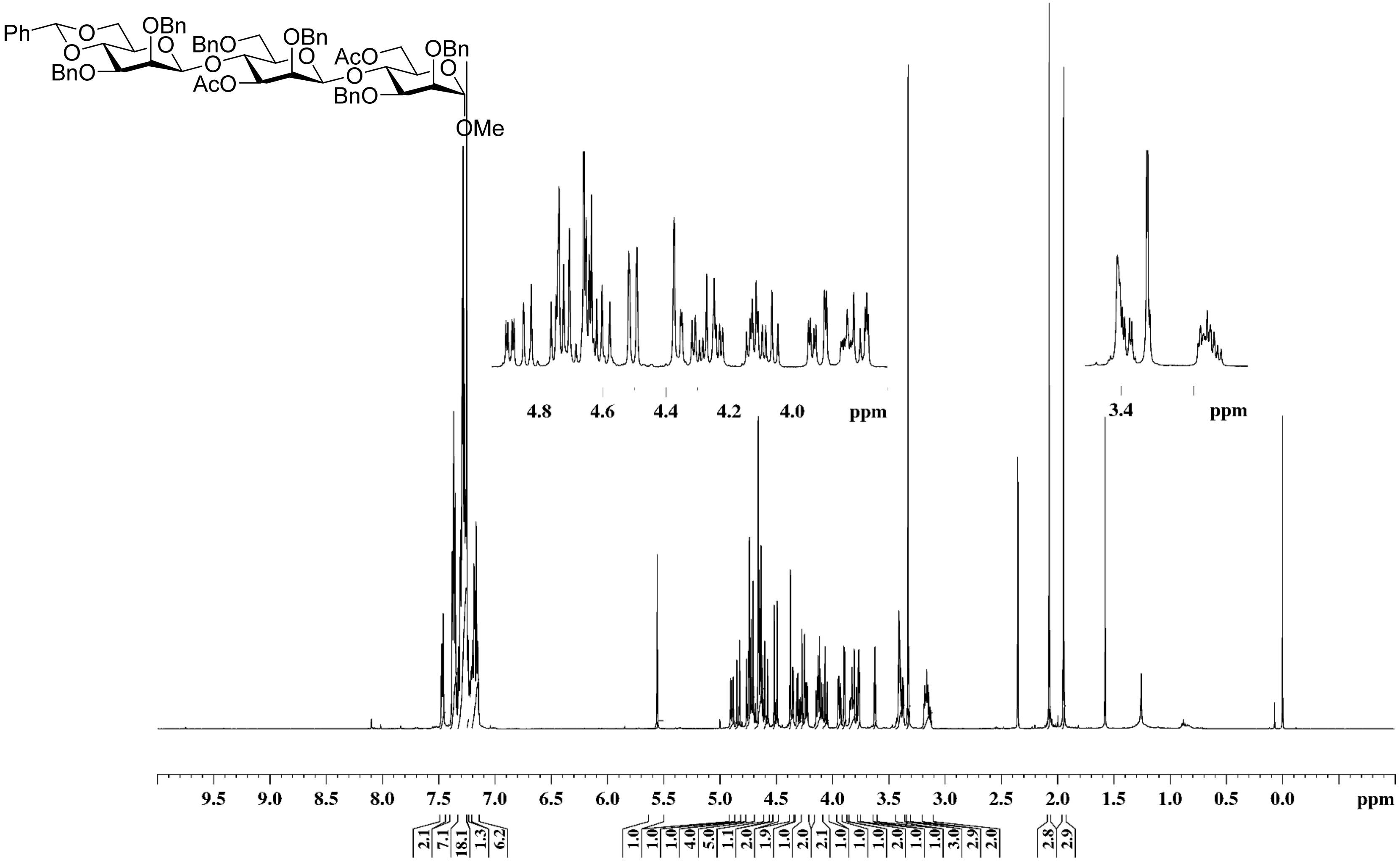

^{13}C NMR (125.8 MHz, $CDCl_3$, 25°C):

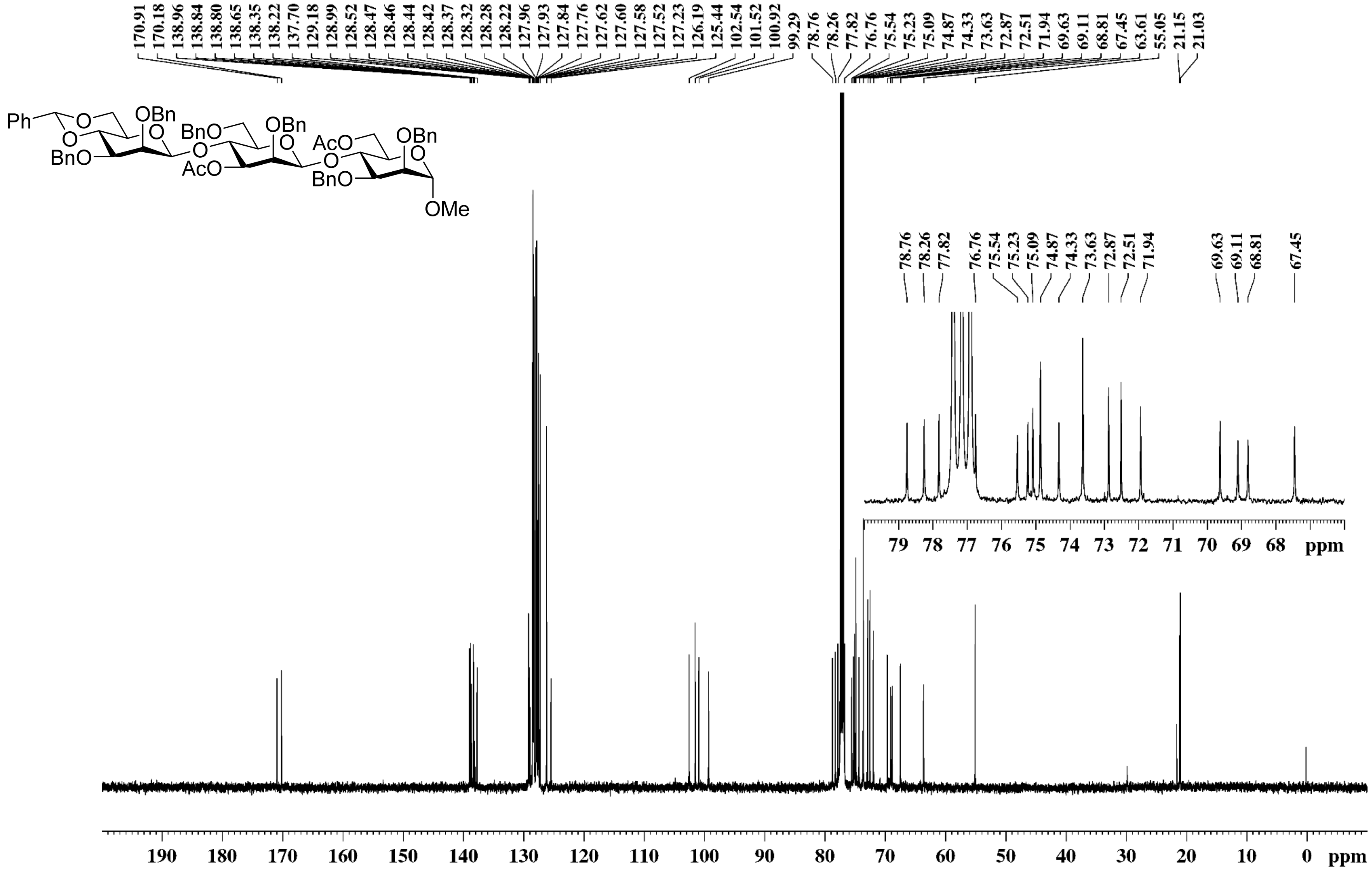

Methyl *O*-(β-D-mannopyranosyl)-(1→4)-*O*-(3-*O*-acetyl-β-D-mannopyranosyl)-(1→4)-6-*O*-acetyl-α-D-mannopyranoside (1b)

^{1}H NMR (500.20 MHz, MeOD, 25°C):

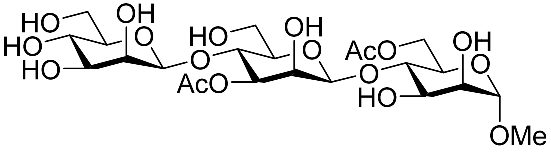

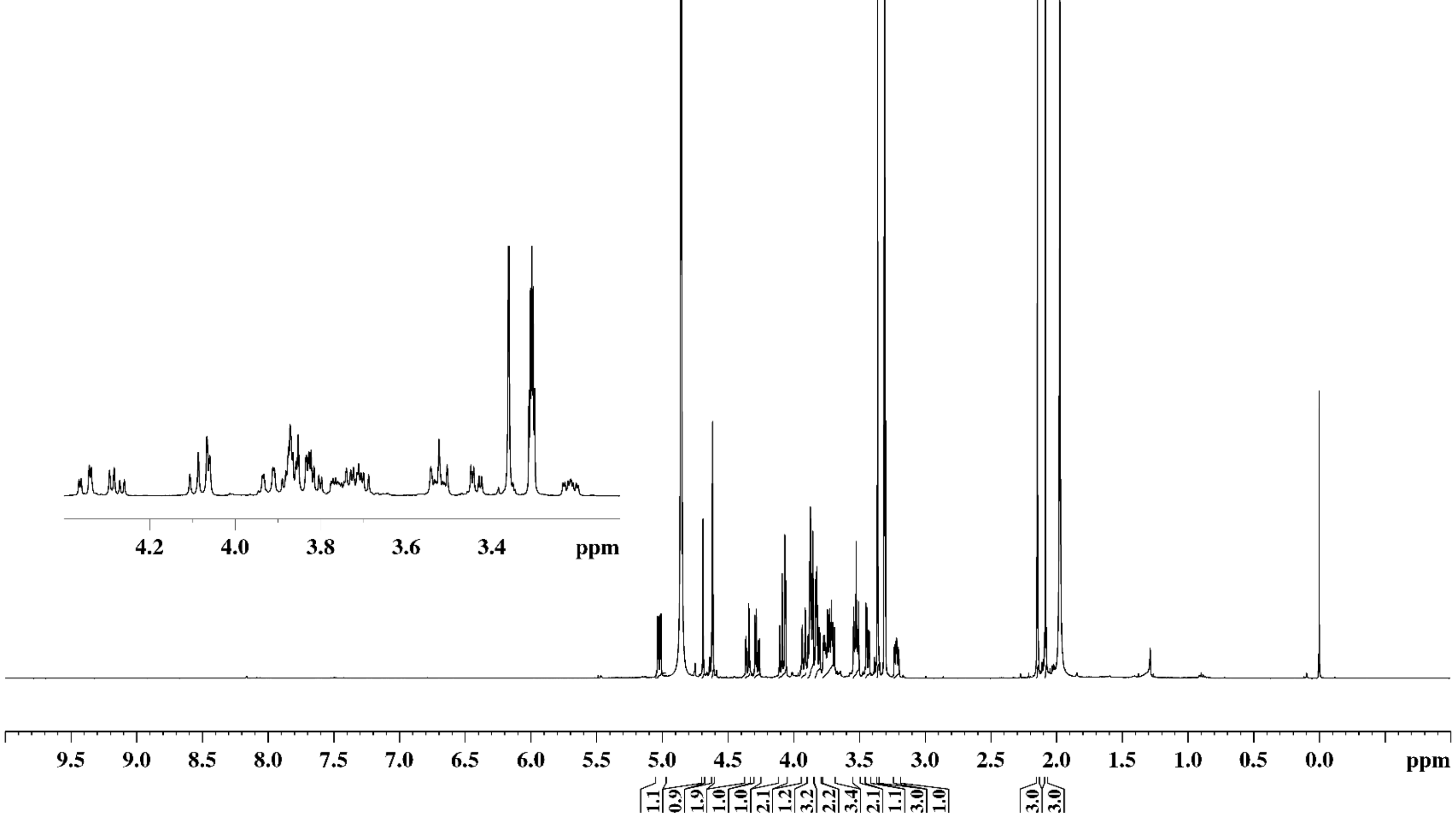

^{13}C NMR (125.8 MHz, MeOD, 25°C):

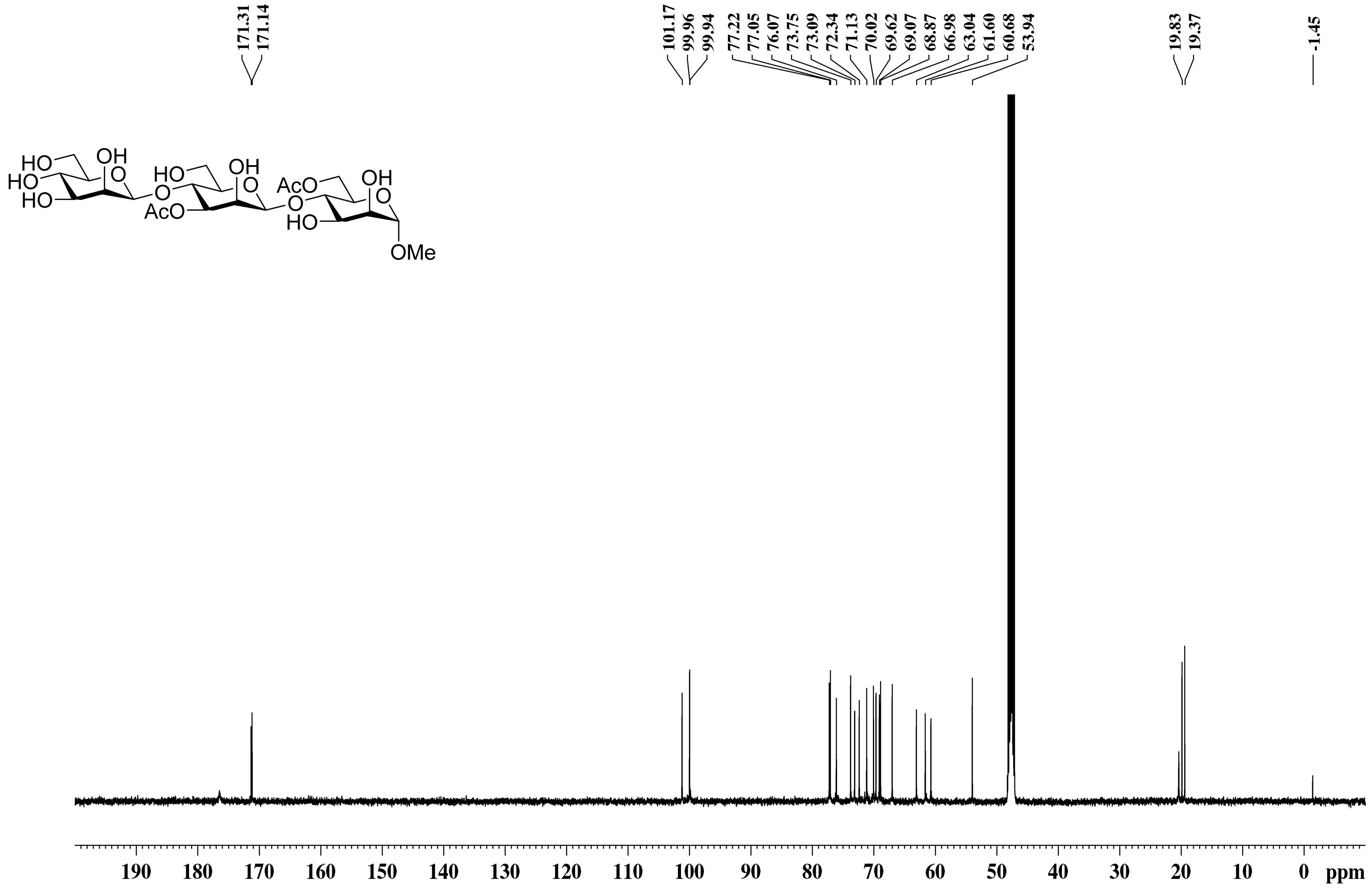

Methyl *O*-(6-*O*-acetyl-2,3-di-*O*-benzyl-β-D-mannopyranosyl)-(1→4)-2,3,6-tri-*O*-benzyl-α-D-mannopyranoside (13)

^{1}H NMR (500.20 MHz, $CDCl_3$, 25°C):

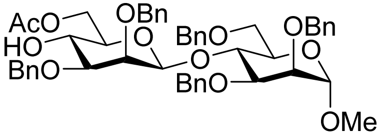

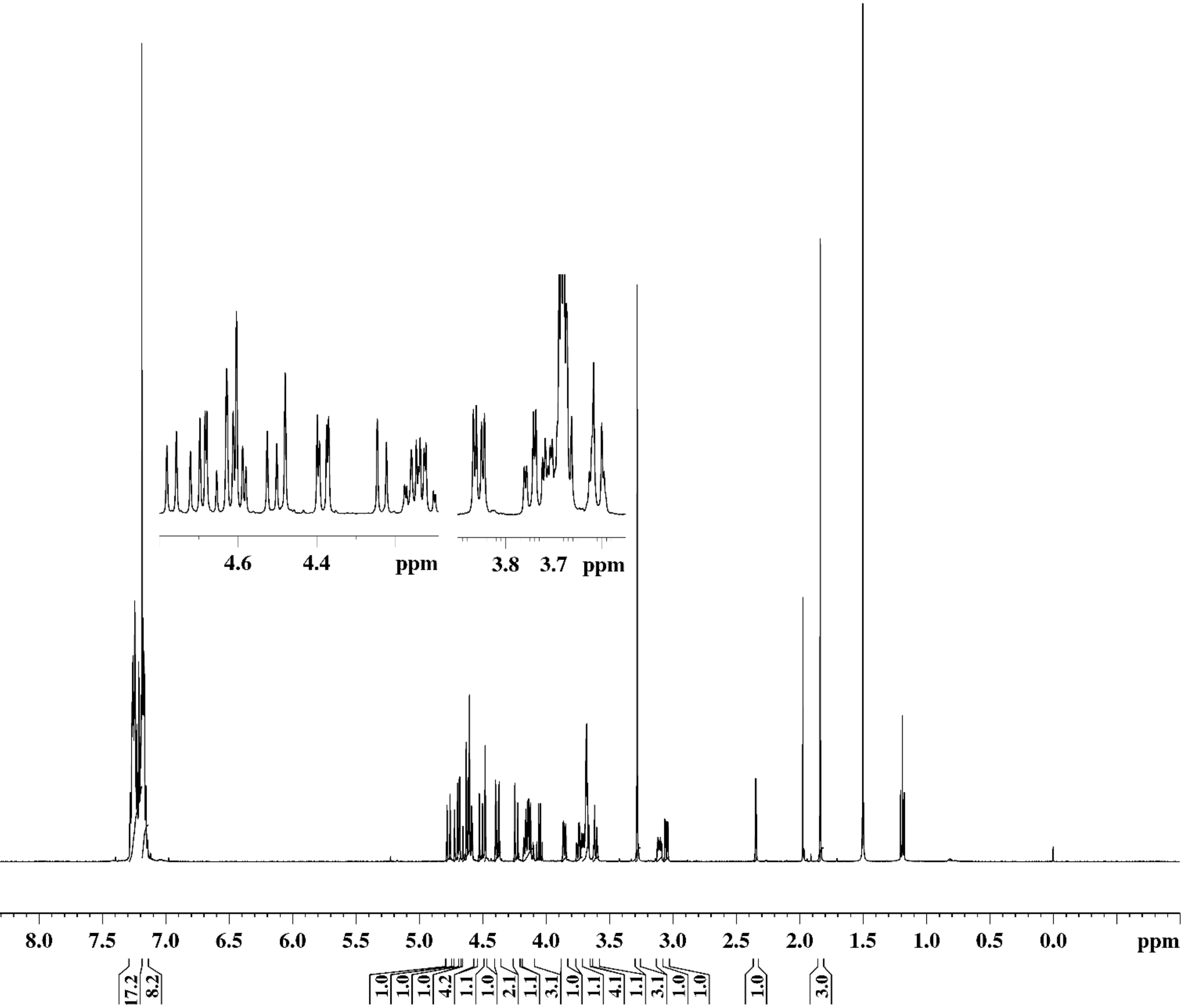

^{13}C NMR (125.8 MHz, $CDCl_3$, 25°C):

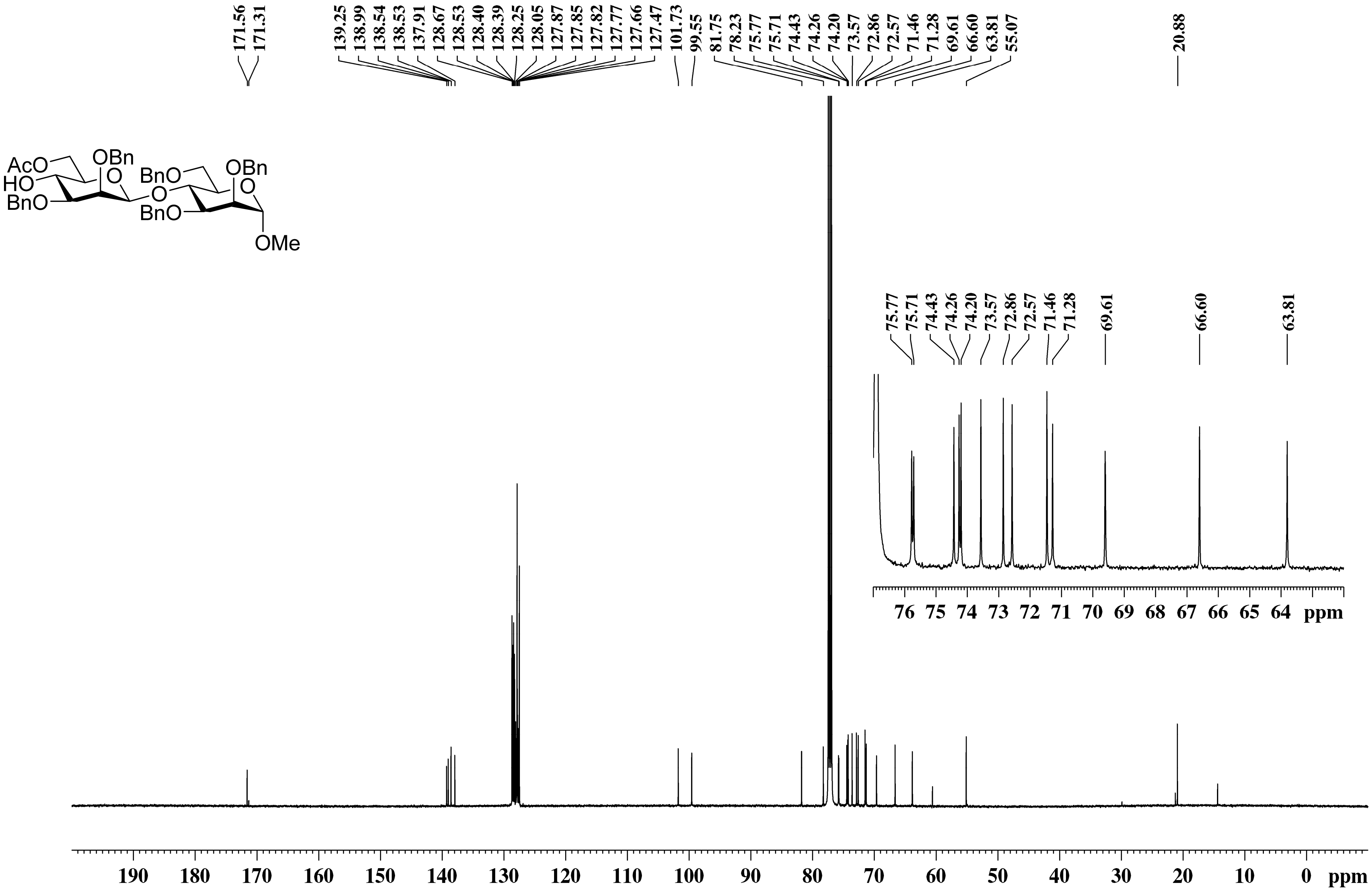

Methyl *O*-(2,3-di-*O*-benzyl-4,6-*O*-benzylidene-β-D-mannopyranosyl)-(1→4)-*O*-(6-*O*-acetyl-2,3-di-*O*-benzyl-β-D-mannopyranosyl)-(1→4)-2,3,6-tri-*O*-benzyl-α-D-mannopyranoside (14)

^{1}H NMR (500.20 MHz, $CDCl_3$, 25°C):

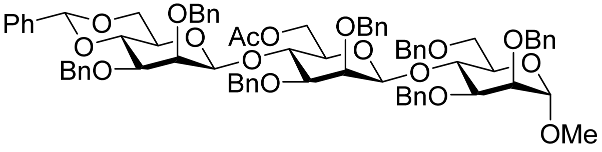

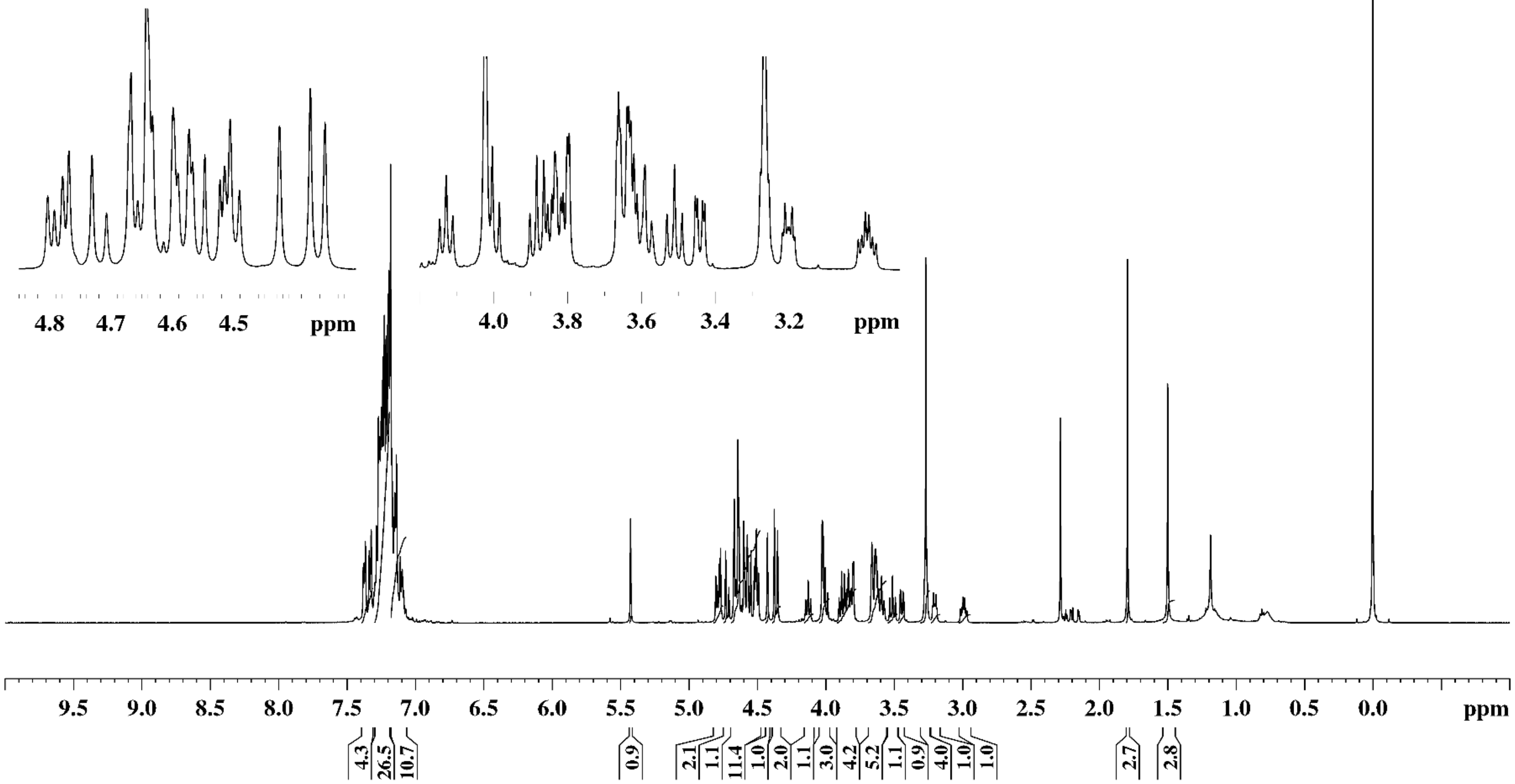

^{13}C NMR (125.8 MHz, $CDCl_3$, 25°C):

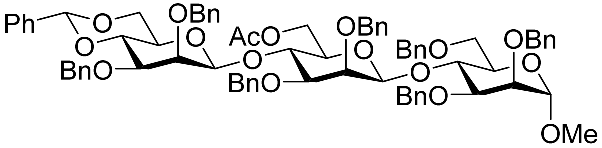

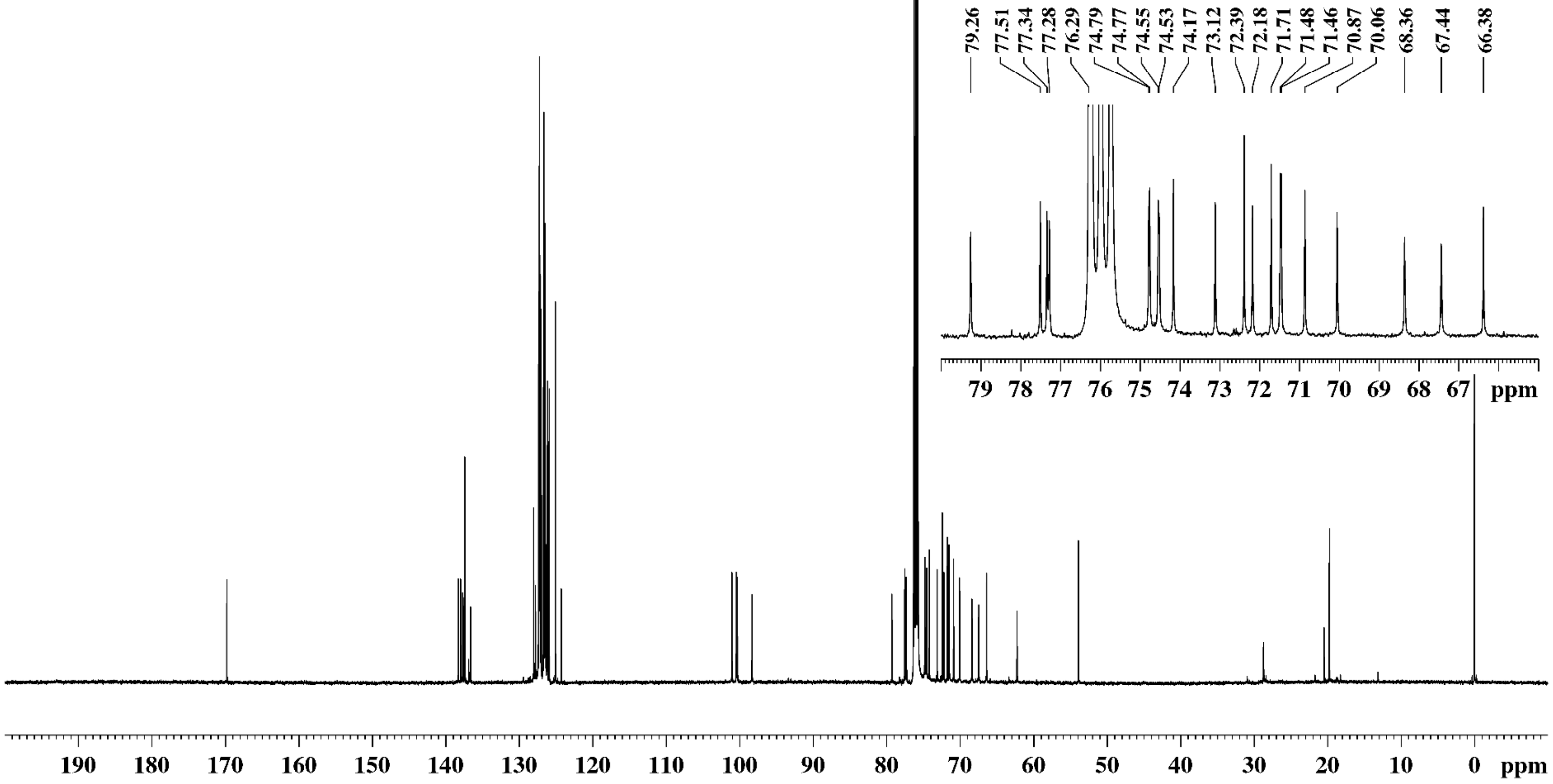

Methyl *O*-(β-D-mannopyranosyl)-(1→4)-*O*-(6-*O*-acetyl-β-D-mannopyranosyl)-(1→4)-α-D-mannopyranoside (2)

^{1}H NMR (500.20 MHz, MeOD, 25°C):

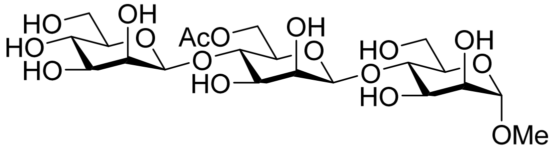

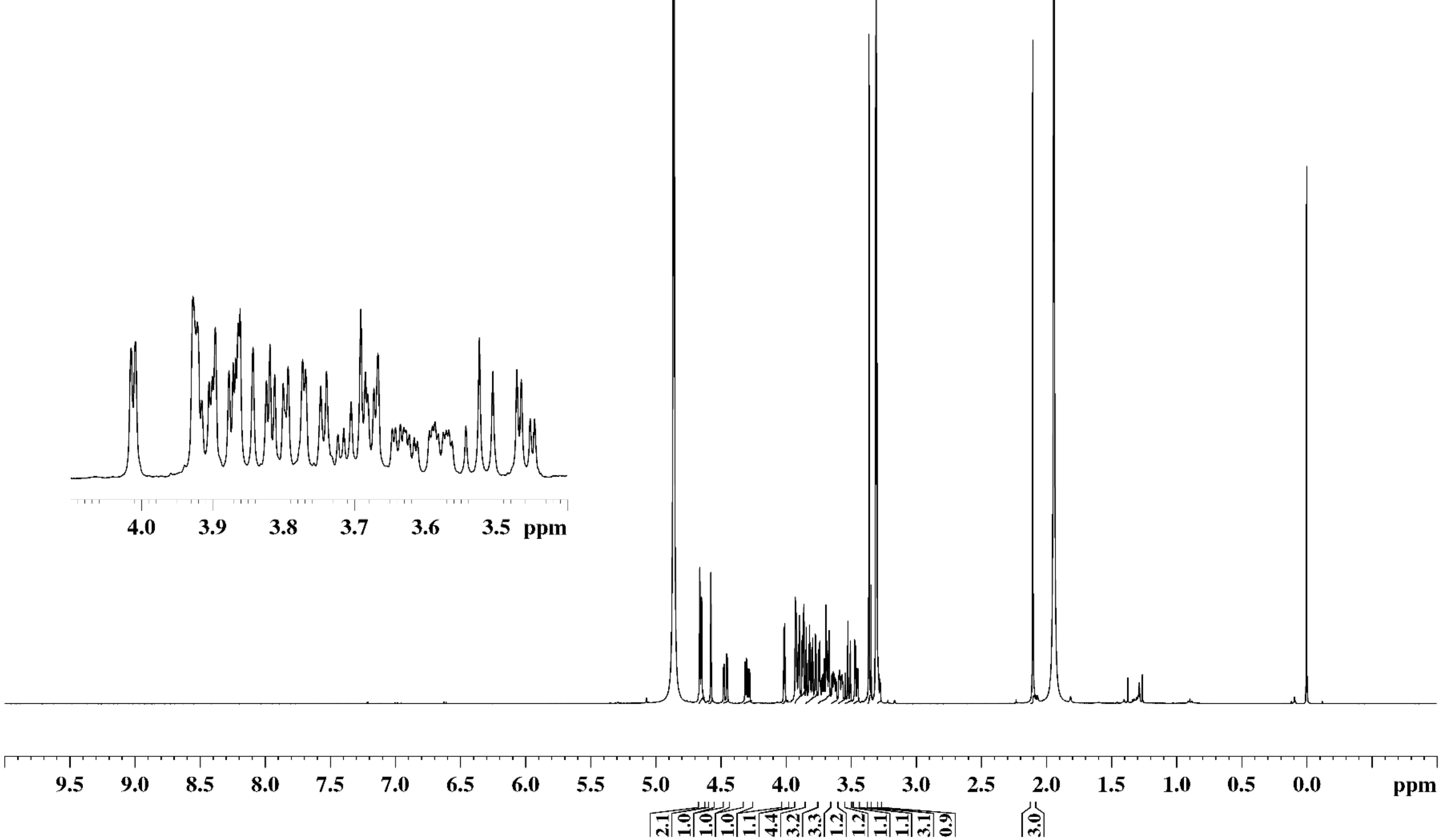

^{13}C NMR (125.8 MHz, MeOD, 25°C):

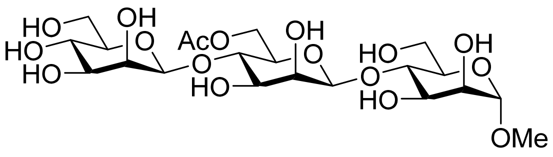

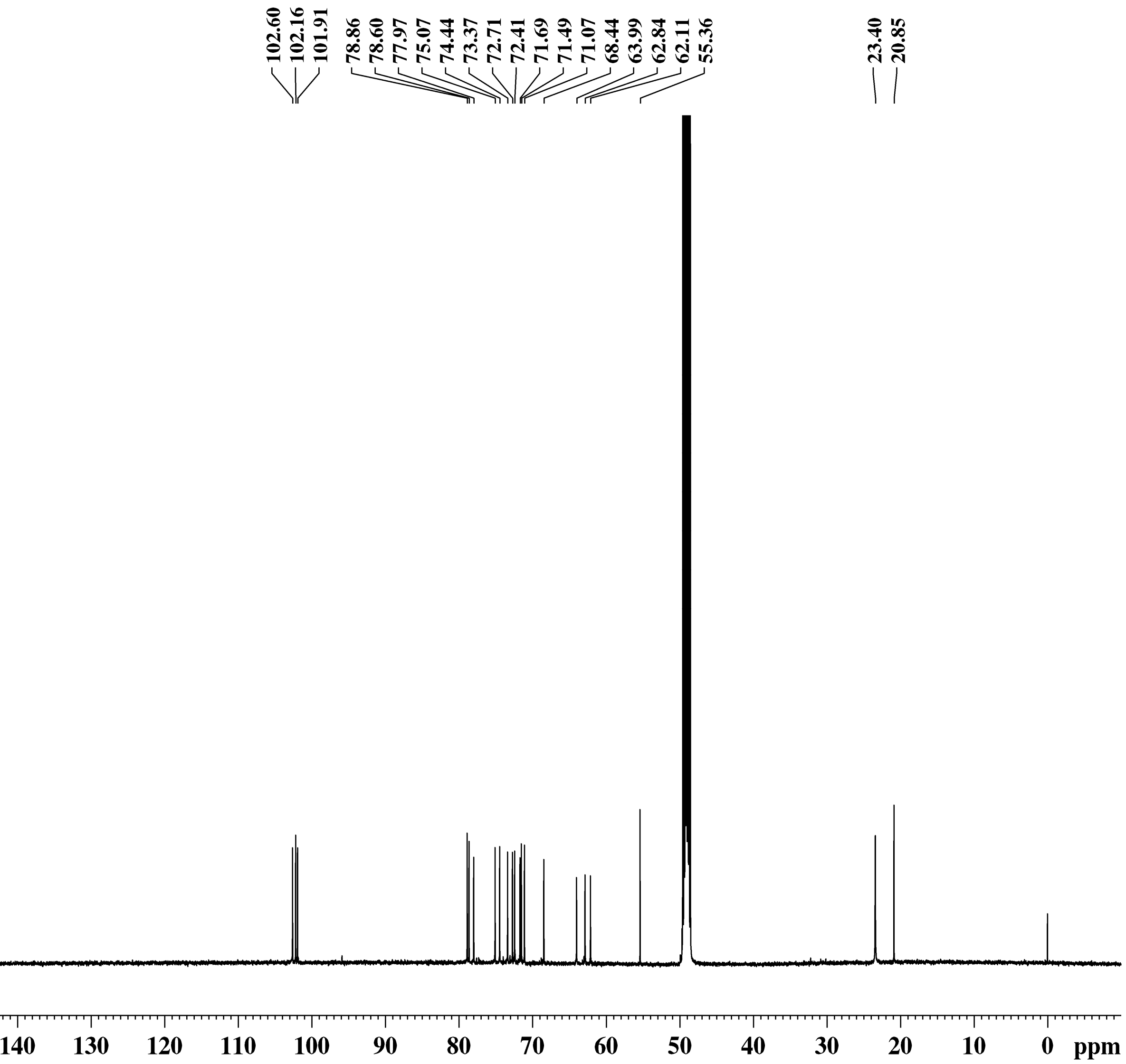

Supplement: Supplementary file 1 — Supporting Information [file CBIC-22-2986-s001.pdf]
